# Supplementary material for: CCR4‐NOT differentially controls host versus virus poly(a)‐tail length and regulates HCMV infection
Source: EMBO Rep. 2023 Oct 17;24(12):e56327. doi: 10.15252/embr.202256327 (PMC10702830; doi:10.15252/embr.202256327)
Supplement: Supplementary file 1 — Expanded View Figures PDF [file EMBR-24-e56327-s003.pdf]

Expanded View Figures

Figure EV1. siRNA mini-screen controls.

A Target depletion for each siRNA was assessed by qRT-PCR. NHDF cells were transfected with 20 nM of either the nonsilencing control or the targeting siRNA as indicated and RNA isolated for analysis at 72 h post-transfection. $n = 2$ biological replicates.

B Cell viability following transfection with each mini-screen siRNA was assessed by quantifying cell number by DAPI nuclear staining and high content imaging at 72 h post-transfection. Each experiment was conducted 3 times with internal duplicates, normalized to control siRNA-treated cells and plotted as the mean ± SEM.

C The impact of Xrn1 depletion on released infectious viral titer was determined by replicating experimental conditions in (Fig 1B) in 12-well plates and establishing TCID50 from culture supernatants on NHDF cells, plotted as the mean ± SEM ($n = 3$ biological replicates). Statistical significance established by ANOVA test with Dunnett multiple comparison correction compared with control siRNA (ns) $P > 0.033$.

D NHDFs were transfected with siRNAs (#1 or #2) targeting each CCR4-NOT nuclease at 20 nM each or control siRNA at 80 nM. Cells were subsequently infected with HCMV (AD169) at low MOI (0.05). Protein lysates were collected at 7 DPI at immunoblotted for viral proteins and a loading control (GAPDH) as indicated.

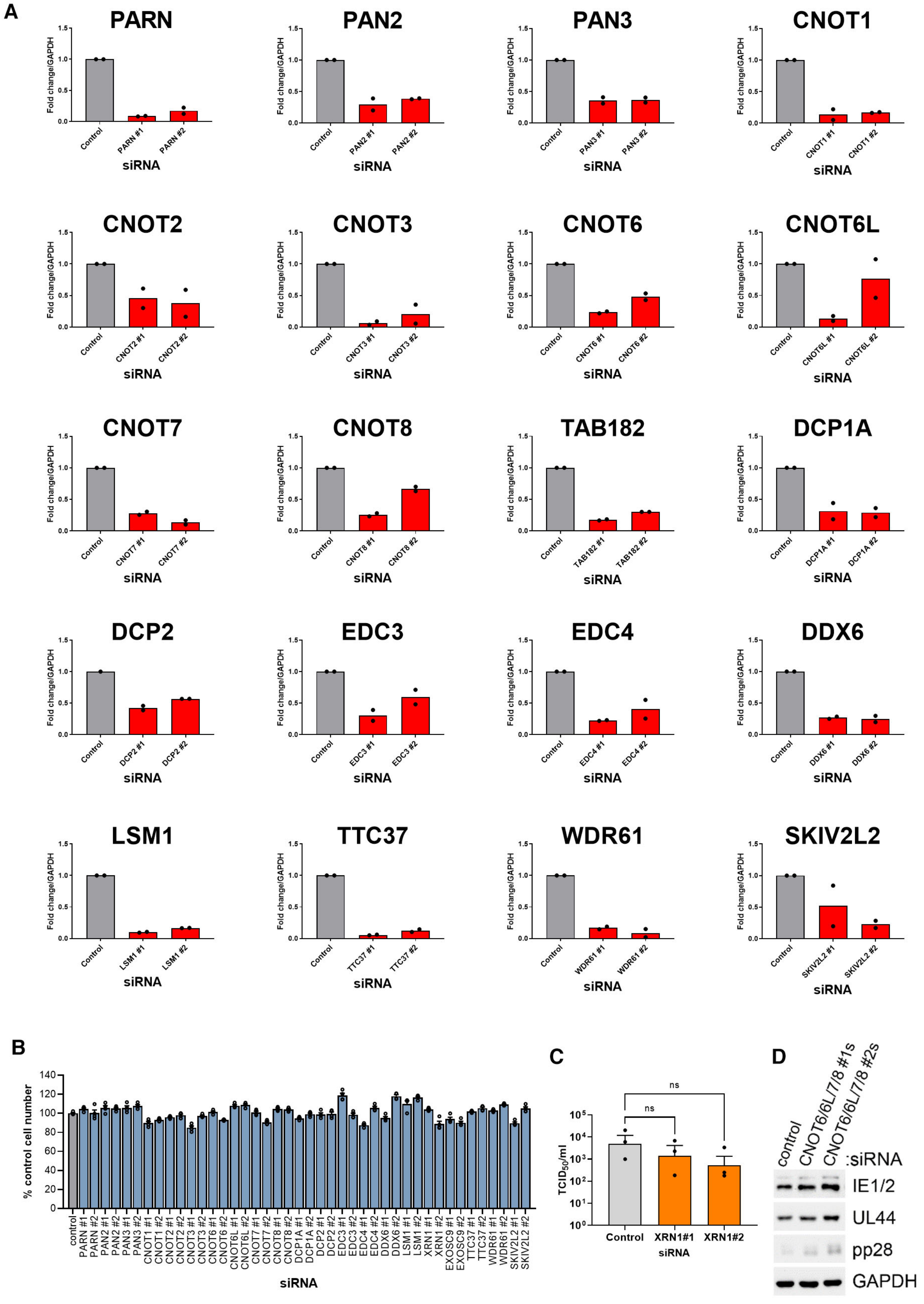

Figure EV1.

 © 2023 The Authors

Figure EV2. CNOT1 and CNOT3 promote viral gene expression late in HCMV infection.

A RT-qPCR analysis for CNOT1 and CNOT3 mRNAs from RNA isolated from uninfected cells treated as in Fig 2A. Mean fold changes relative to GAPDH ± SEM (n = 3 biological replicates) are plotted with statistical significance established by ANOVA test with Dunnett multiple comparison correction compared control siRNA treated samples; (*) $P < 0.033$, (**) $P < 0.002$, (***) $P < 0.001$, no asterisk: not significant.

B RT-qPCR analysis for viral mRNAs from RNA isolated from cells siRNA-transfected and infected with HCMV AD169 as in Fig 2C. Protein names, where different from gene names, indicated in brackets. Mean fold changes relative to GAPDH ± SEM (n = 3 biological replicates) are plotted, normalized to siControl 6 HPI samples.

C Cells stained positively for IE1/2 expression by immunofluorescence were scored at 6HPI following infection (MOI: 3) of control and CNOT1/3 siRNA-transfected NHDFs. Mean % IE1/2 positive cells are plotted ± SEM (n = 3 biological replicates). No significant (ns) differences between control and knockdown cells were identified by ANOVA test with Dunnett multiple comparison correction.

D Immunoblot analysis of lysates from NHDF cells transfected with control or CNOT3 siRNA (#1) and infected with HCMV AD169 at MOI = 3 and analyzed as in Fig 2C.

E Immunoblot analysis of lysates from NHDF cells transfected with control or CNOT1 siRNA (#1) and infected with HCMV TB40/E at MOI = 3 and analyzed as in Fig 2C.

F Titer of infectious released virus was determined by TCID50 on supernatants of cells transfected with the indicated siRNAs and infected with HCMV clinical strain TB40/E at MOI = 0.05 and incubated for 7 days. Mean TCID50/ml of two biological experiments shown.

© 2023 The Authors

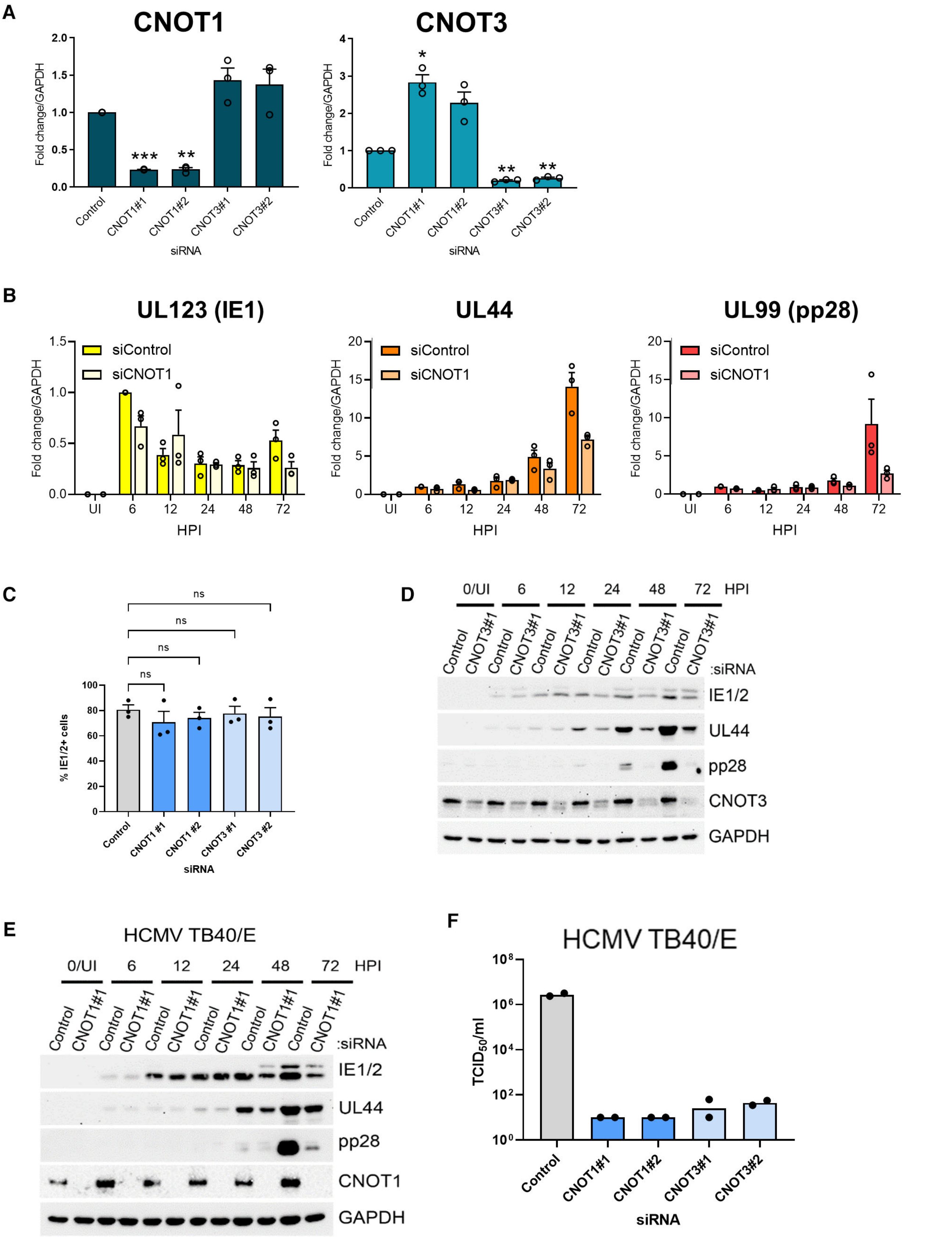

Figure EV2.

 © 2023 The Authors

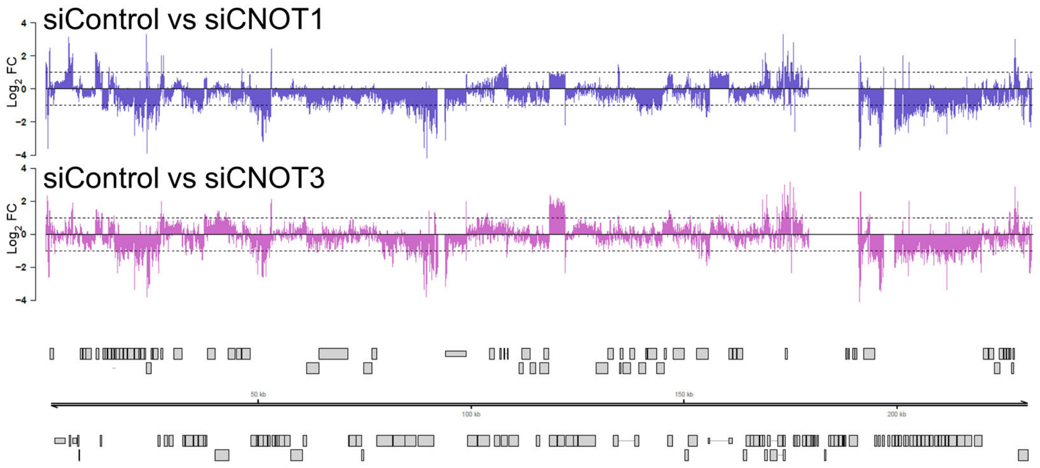

Figure EV3. Comparison of viral transcript genomic distribution in control vs. CNOT1/3 knockdown cells.

The distribution of 50,000, randomly sampled viral reads was compared between infected siControl and siCNOT1 cells (upper panel) and siControl and siCNOT3 cells (lower panel) and $\log_2$ fold change plotted across the viral genome. Canonical HCMV ORFs are shown as gray boxes.

Figure EV4. The impact and functional importance of host transcriptome changes upon CNOT1/3 knockdown.

A NHDFs were pretreated with indicated cytokines for 24 h and subsequently infected with HCMV (AD169) at low MOI (0.05). At 7 DPI-infected cells were identified as in Fig 1A using high content imaging. Mean % infected cells ± SEM, normalized to nontreated (NT) cells (n = 3 biological replicates) is plotted. Statistical significance established by ANOVA test with Dunnett multiple comparison correction compared control siRNA-treated samples; (ns) $P > 0.033$, (***) $P < 0.001$.

B Select differentially regulated transcripts were validated by RT-qPCR analysis from control or CNOT1 siRNA-transfected cells that were mock infected or infected with HCMV AD169 at MOI = 3 and collected at 72 HPI. Mean ± SEM (n = 3 biological replicates) fold change relative to GAPDH is plotted.

C siRNAs against host genes differentially expressed by CNOT1/3 knockdown were validated by RT-qPCR 3 days post-transfection of NHDFs.

Data information: Statistical significance in (B) and (C) was tested by students t-tests; (*) $P < 0.05$, (**) $P < 0.01$, (***) $P < 0.001$, no asterisk: not significant.

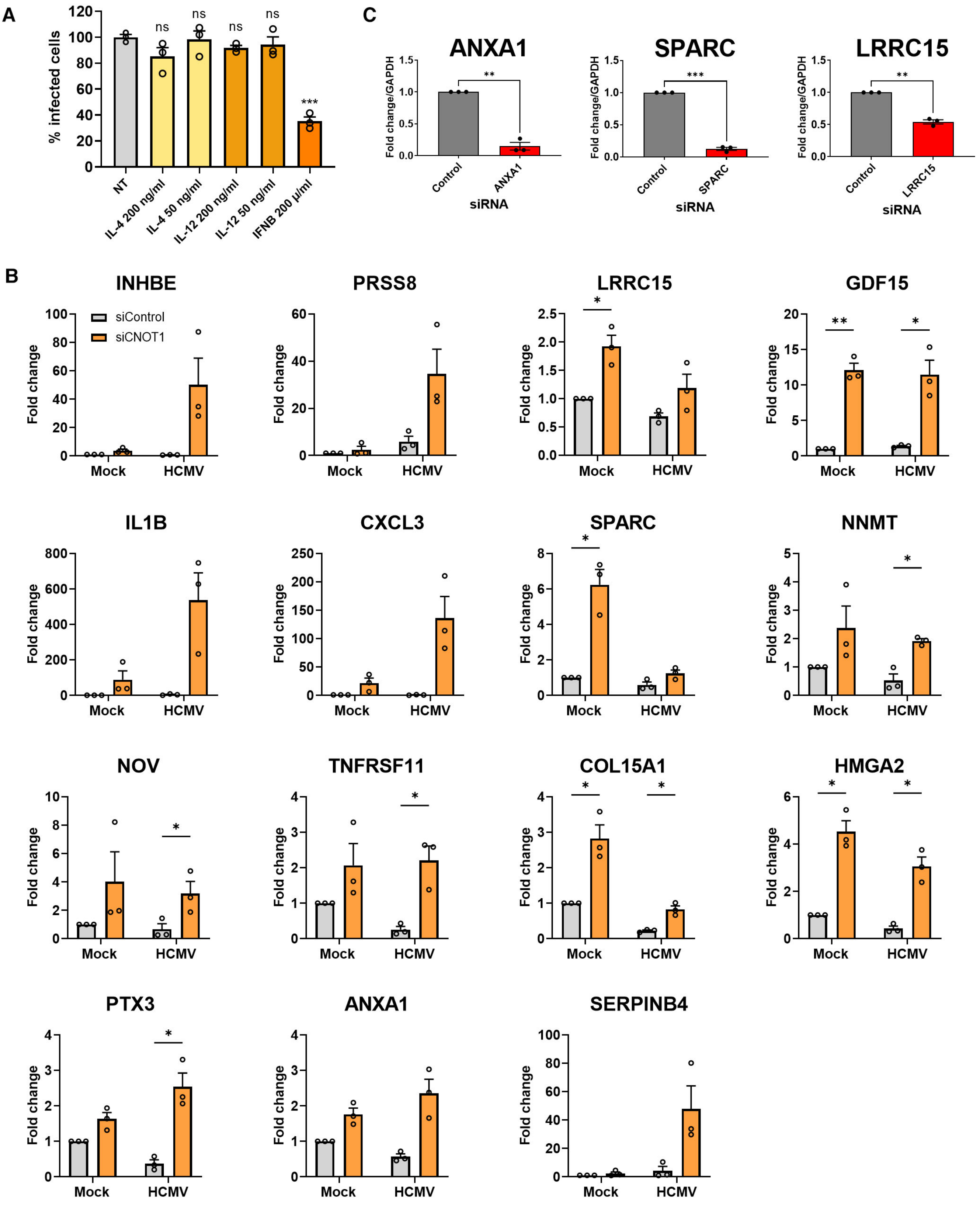

Figure EV4.

© 2023 The Authors

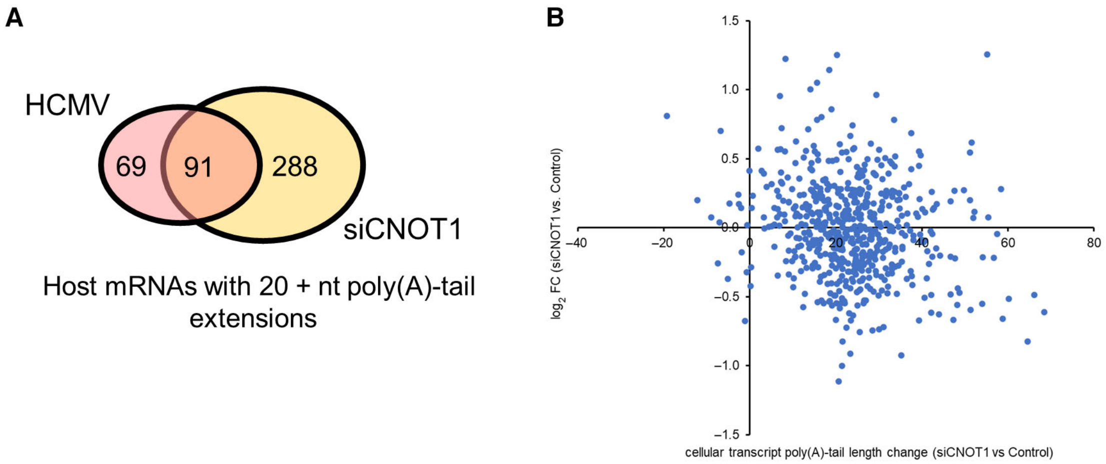

Figure EV5. Comparing poly(A)-tail length changes and RNA abundance changes of cellular transcripts in CNOT1/3-depleted and HCMV-infected cells.

A Venn diagram showing overlap of host genes detected with > 20 reads for which ≥ 20 nt poly(A) tail length additions were found at any time point post-infection (pink) compared to uninfected samples, and in siCNOT1 compared to siControl samples (yellow; average of two biological replicates).

B Expression changes of cellular genes upon CNOT1-depletion (Fig 5) are plotted against poly(A)-tail length changes upon CNOT1-depletion detected by DRS (mean of two biological replicates, Fig 6F).

© 2023 The Authors
