# Supplementary material for: CCR4‐NOT differentially controls host versus virus poly(a)‐tail length and regulates HCMV infection
Source: EMBO Rep. 2023 Oct 17;24(12):e56327. doi: 10.15252/embr.202256327 (PMC10702830; doi:10.15252/embr.202256327)
Supplement: Supplementary file 8 — PDF+ [file EMBR-24-e56327-s014.pdf]

Article

SOURCE DATA · TRANSPARENT PROCESS · OPEN ACCESS

EMBO reports

CCR4-NOT differentially controls host versus virus poly(a)-tail length and regulates HCMV infection

Hannah M Burgess[1,2,*], Rebecca Grande[2], Sofia Riccio[1], Ikshitaa Dinesh[1], Gerlof Sebastiaan Winkler[3], Daniel P Depledge[2,4,5] & Ian Mohr[2,6]

Abstract

Unlike most RNA and DNA viruses that broadly stimulate mRNA decay and interfere with host gene expression, human cytomegalovirus (HCMV) extensively remodels the host translatome without producing an mRNA decay enzyme. By performing a targeted loss-of-function screen in primary human fibroblasts, we here identify the host CCR4-NOT deadenylase complex members CNOT1 and CNOT3 as unexpected pro-viral host factors that selectively regulate HCMV reproduction. We find that the scaffold subunit CNOT1 is specifically required for late viral gene expression and genome-wide host responses in CCR4-NOT-disrupted cells. By profiling poly (A)-tail lengths of individual HCMV and host mRNAs using nanopore direct RNA sequencing, we reveal poly(A)-tails of viral messages to be markedly longer than those of cellular mRNAs and significantly less sensitive to CCR4-NOT disruption. Our data establish that mRNA deadenylation by host CCR4-NOT is critical for productive HCMV replication and define a new mechanism whereby herpesvirus infection subverts cellular mRNA metabolism to remodel the gene expression landscape of the infected cell. Moreover, we expose an unanticipated host factor with potential to become a therapeutic anti-HCMV target.

Keywords CCR4-NOT; deadenylation; HCMV; RNA decay; virus:host interaction

Subject Categories Microbiology, Virology & Host Pathogen Interaction; RNA Biology

DOI 10.15252/embr.202256327 | Received 18 October 2022 | Revised 25 September 2023 | Accepted 28 September 2023

EMBO Reports (2023) e56327

Introduction

Successful reproduction of viruses in host cells depends on effective commandeering of cellular protein synthesis machinery and evading detection by, or resisting the action of, host antiviral immune responses. During lytic infection with the α-herpesvirus herpes simplex 1 (HSV-1), "shut-off" of host protein synthesis occurs, accomplished by multiple mechanisms including the expression of a virally encoded endoribonuclease that accelerates mRNA decay (Kwong & Frenkel, 1987; Rutkowski *et al*, 2015; Hennig *et al*, 2021). This manipulation of infected cell mRNA decay dynamics not only facilitates the elimination of host mRNA competition for ribosome access, but ensures the disposal of mRNAs encoding antiviral products that may be induced and helps steer transitioning through the temporal cascade of gene expression crucial for completion of the herpesviral lifecycle (Pasieka *et al*, 2008). Furthermore, formation from complementary viral transcripts of double-stranded (ds) RNA, a major trigger of antiviral cellular responses, and ribonucleoprotein granules that can potentiate dsRNA sensing in infected cells, are suppressed (Dauber *et al*, 2011, 2019; Burgess & Mohr, 2018). An effective strategy, a similar stimulation of RNA decay by viral proteins is observed in other virus families such as poxviruses, bunyaviruses, and coronaviruses, while host mRNA decay activities may also be annexed to achieve reproduction success (Burgess *et al*, 2022).

The β-herpesvirus human cytomegalovirus (HCMV) provides a model to explore viral co-option of host mRNA decay in the absence of virus-encoded decay factors. A near ubiquitous human pathogen, HCMV is rarely symptomatic in immunocompetent individuals. A latent infection is established in myeloid progenitor cells and maintained for life following an initial infection, with reactivation and a switch to lytic reproduction occurring upon a variety of cellular cues. In the immunocompromised such reactivation can cause severe disease and lead to graft rejection in transplant recipients. Congenital infections can also result in serious neurological consequences to newborns, such as impaired cognitive development and hearing loss, and place HCMV as a leading infectious cause of birth defects (Boeckh & Geballe, 2011; Griffiths *et al*, 2015). However, there is presently no effective HCMV vaccine and therapeutic options are limited and incur significant drawbacks such as long-term toxicity and poor bioavailability (Perera *et al*, 2021; Scarpini *et al*, 2021).

1 Department of Microbial Sciences, University of Surrey, Guildford, UK
2 Department of Microbiology, School of Medicine, New York University, New York, NY, USA
3 School of Pharmacy, University of Nottingham, Nottingham, UK
4 Institute of Virology, Hannover Medical School, Hannover, Germany
5 German Center for Infection Research (DZIF), partner site Hannover-Braunschweig, Hannover, Germany
6 Laura and Isaac Perlmutter Cancer Institute, School of Medicine, New York University, New York, NY, USA
*Corresponding author. Tel: +44 (0)1483684556; E-mail: h.burgess@surrey.ac.uk

© 2023 The Authors. Published under the terms of the CC BY 4.0 license

Infection with HCMV results, rather than shut-off, in an overall stimulation of protein synthesis with viral proteins produced alongside those of the host cell (McKinney *et al*, 2014). To engineer this increase in protein synthetic capacity, multiple layers of translational control are manipulated and the abundance of many translation factors, including cytoplasmic poly(A)-binding protein 1 (PABPC1), as well as ribosomal proteins is upregulated (Walsh *et al*, 2005; McKinney *et al*, 2012; Tirosh *et al*, 2015; Bianco & Mohr, 2019; Thompson *et al*, 2022). Host gene expression is nevertheless distinctly manipulated by HCMV, and significant changes in host protein abundances have been comprehensively characterized in lytically infected fibroblasts (Weekes *et al*, 2014). Changes to RNA abundance accounts for much of this manipulation of host gene expression, with translational regulation acting on specific sets of host genes (McKinney *et al*, 2014; Tirosh *et al*, 2015; Thompson *et al*, 2022).

Poly(A)-tails are major determinants of mRNA stability and translation by providing a platform for the binding of PABPCs (Passmore & Coller, 2022). In mammalian cells, most protein coding mRNAs are bestowed a long poly(A)-tail of around 250 nucleotides (nt) following transcriptional termination in the nucleus. This length is dictated by the cleavage and polyadenylation specificity factor complex (CPSF) and binding to the nascent poly(A) tail of successive molecules of nuclear PABP (PABPN1), which is structurally distinct from its cytoplasmic counterpart (Eckmann *et al*, 2011). As herpesviral transcripts are generated in the nucleus by RNA polymerase II, they are expected to be subject to the same polyadenylation process. Cellular deadenylase complexes PAN2/3 and CCR4-NOT subsequently trim the tail in the cytoplasm and a bulk population of cellular mRNAs with tail lengths between 30 and 100 nt, equivalent to the footprints of 1–3 PABPC molecules, are commonly detected by global poly(A)-tail sequencing approaches (Chang *et al*, 2014; Workman *et al*, 2019; Mattijssen *et al*, 2020). Regarded as the rate limiting step in mRNA turnover, full deadenylation ultimately commits mRNAs to exonucleolytic decay either 3′-5′ by the RNA exosome or 5′-3′ by Xrn1 subsequent to decapping by the Dcp1/2 complex (Passmore & Coller, 2022).

In this study, we leverage high-content imaging to establish the importance of host deadenylation-dependent mRNA turnover factors on HCMV replication and surprisingly identify the deadenylase complex CCR4-NOT as an important regulator of HCMV. Depletion of CCR4-NOT components led to a global reduction in viral RNA expression, viral DNA synthesis, and impaired viral protein production late in infection, and selectively impacted host responses. Remarkably, however, though our results demonstrate that CCR4-NOT shortens poly(A)-tail lengths in infected cells and this function is necessary for efficient lytic HCMV replication, we find this activity preferentially targets host mRNAs, revealing a hitherto undefined layer of viral subversion of host RNA regulation.

Results

Identification of CCR4-NOT components as HCMV regulators in a mRNA decay mini-screen

To determine whether HCMV replication relies on components of the cellular canonical mRNA turnover pathway, we curated a list of 20 genes to target with two distinct siRNAs each in a focused RNAi mini-screen in primary normal human dermal fibroblasts (NHDFs; Fig 1A). The ability of each siRNA to deplete its target mRNA was validated by RT-qPCR (Fig EV1A), and no substantial effects on cell viability were detected for any siRNA treatment (Fig EV1B). siRNA-treated NHDFs in 96-well plates were subsequently infected at low multiplicity with HCMV which was allowed to spread through the cell monolayer for 7 days, permitting at least one round of viral replication. To measure the impact on the virus spread, infected cells were identified by indirect immunofluorescence for immediate early (IE) proteins 1 and 2 and quantified using a high-content imaging platform (Fig 1A). Compared to nonsilencing control siRNA treated cultures, only silencing of CCR4-NOT complex components CNOT1 or CNOT3 resulted in a significant reduction in infected cell number upon transfection with both target-specific siRNAs (Fig 1B). Depletion of CNOT1 led to an 85 and 72% reduction in infection with siRNAs #1 and #2, respectively, while CNOT3 targeting led to reductions of 92 and 80%. A significant inhibitory effect on viral spread was also observed upon transfection with CNOT2 siRNA #1, though siRNA #2 also led to a modest reduction (18%) that did not reach statistical significance.

The CCR4-NOT complex is a large multisubunit complex (Fig 1C) that deadenylates the poly(A)-tails of mRNAs in the cytoplasm. CNOT1 is the large scaffolding protein upon which the CCR4-NOT complex is assembled and facilitates many interactions with RNA-binding proteins (RBPs) that recruit CCR4-NOT to mRNAs. The complex features two nucleases named Caf1 and CCR4 that differ in their ability to degrade free poly(A)-tails and those bound by PABPCs (Yi *et al*, 2018). In humans, Caf1 is provided by either of two homologous proteins, CNOT7 or CNOT8, and CCR4 by either CNOT6 or CNOT6L. Combined knockdown of each nuclease, however, also did not impact viral replication (Fig EV1D). While our knockdowns were each effective (Fig EV1A), residual levels of nuclease enzymes may be sufficient to supply CCR4-NOT with deadenylase activity. By contrast depletion of scaffold subunit CNOT1 is predicted to prevent complex assembly, likely explaining the differing impact on HCMV replication of targeting these different components of the same complex. To establish whether the decrease in spread observed within cell monolayers (Fig 1B) affected release of infectious virus, supernatants from RNAi-treated cultures were subject to TCID50 (50% tissue culture infectious dose) assay. Compared to nonsilencing control siRNA-treated cultures, depletion of CNOT1 or CNOT3 significantly reduced virus replication up to 2,000-fold (Fig 1D) validating our screening approach. By contrast, knockdown of Xrn1 led to a far smaller reduction in viral titer of 3- to 7-fold that did not reach statistical significance (Fig EV1C). Together, these results implicate the CCR4-NOT deadenylase complex in the regulation of HCMV reproduction and spread.

CNOT1 and CNOT3 are required for viral RNA and protein accumulation late in infection

To decipher how CNOT1 and CNOT3 promote viral replication, we examined the abundance of viral proteins and transcripts in a synchronous high MOI (multiplicity of infection) infection. At 72 h post infection (HPI), late in the virus lifecycle, accumulation of representative proteins from each temporal gene class was impaired when CNOT1 or CNOT3 were targeted by either of two independent

 © 2023 The Authors

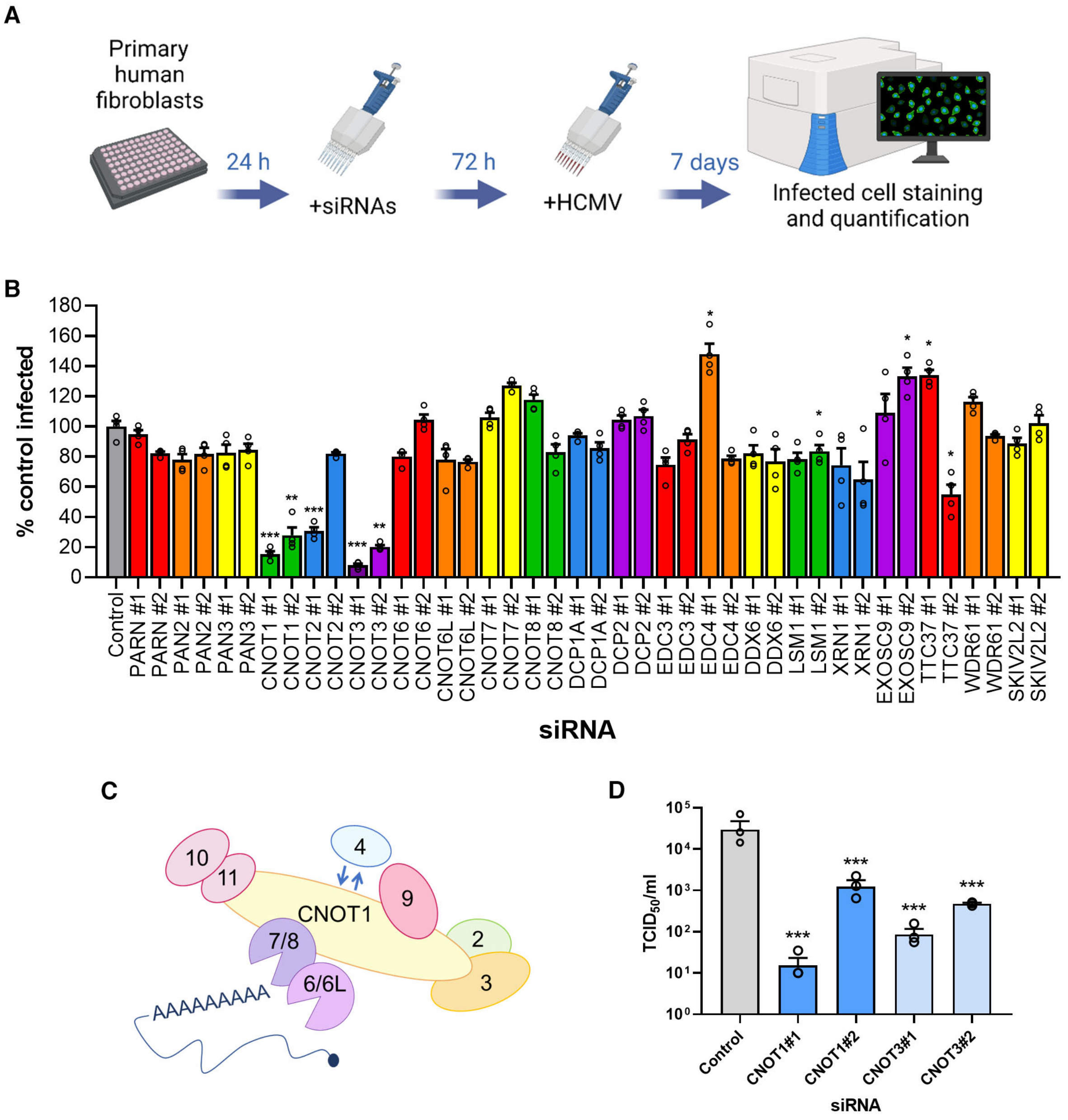

Figure 1. An siRNA mini-screen identifies CCR4-NOT components as regulators of HCMV replication.

A Experimental set up for siRNA screen. Normal human dermal fibroblasts (NHDFs) were transfected at 20 nM with two independent siRNAs targeting each of 20 genes in 96-well plates. Cells were subsequently infected with HCMV (AD169) at low MOI (0.05) and cells fixed 7 days post infection. Cells were identified by DAPI nuclear staining, and HCMV-positive cells were identified by immunostaining for immediate early proteins and quantified in each entire well using high content imaging (Thermofisher CellInsight CX7 LZR).

B Screen results showing mean % infected cells (±SEM) of four biological experiments with technical duplicates, normalized to control siRNA treated cells. Statistical significance was established by ANOVA test with Dunnett multiple comparison correction compared with control siRNA, (*) $P < 0.033$, (**) $P < 0.002$, (***) $P < 0.001$, no asterisk: not significant.

C Schematic of human CCR4-NOT complex.

D The impact of CNOT1 and CNOT3 depletion on released infectious viral titer was determined by replicating experimental conditions in (B) in 12-well plates and establishing TCID50 from culture supernatants on NHDF cells, plotted as the mean ± SEM ($n = 3$ biological replicates). Statistical significance established by ANOVA test with Dunnett multiple comparison correction compared with control siRNA, (***) $P < 0.001$.

Source data are available online for this figure.

siRNAs (Fig 2A). Immediate-early (IE) proteins (IE1/2), master transcriptional activators, were modestly reduced and early protein (UL44), required for viral DNA synthesis, was reduced to a greater extent (Fig 2A). The product of essential late-expressed gene UL99, pp28, however, was scarcely detected in knockdown cells (Fig 2A). RT-qPCR for the mRNAs encoding the same proteins revealed a

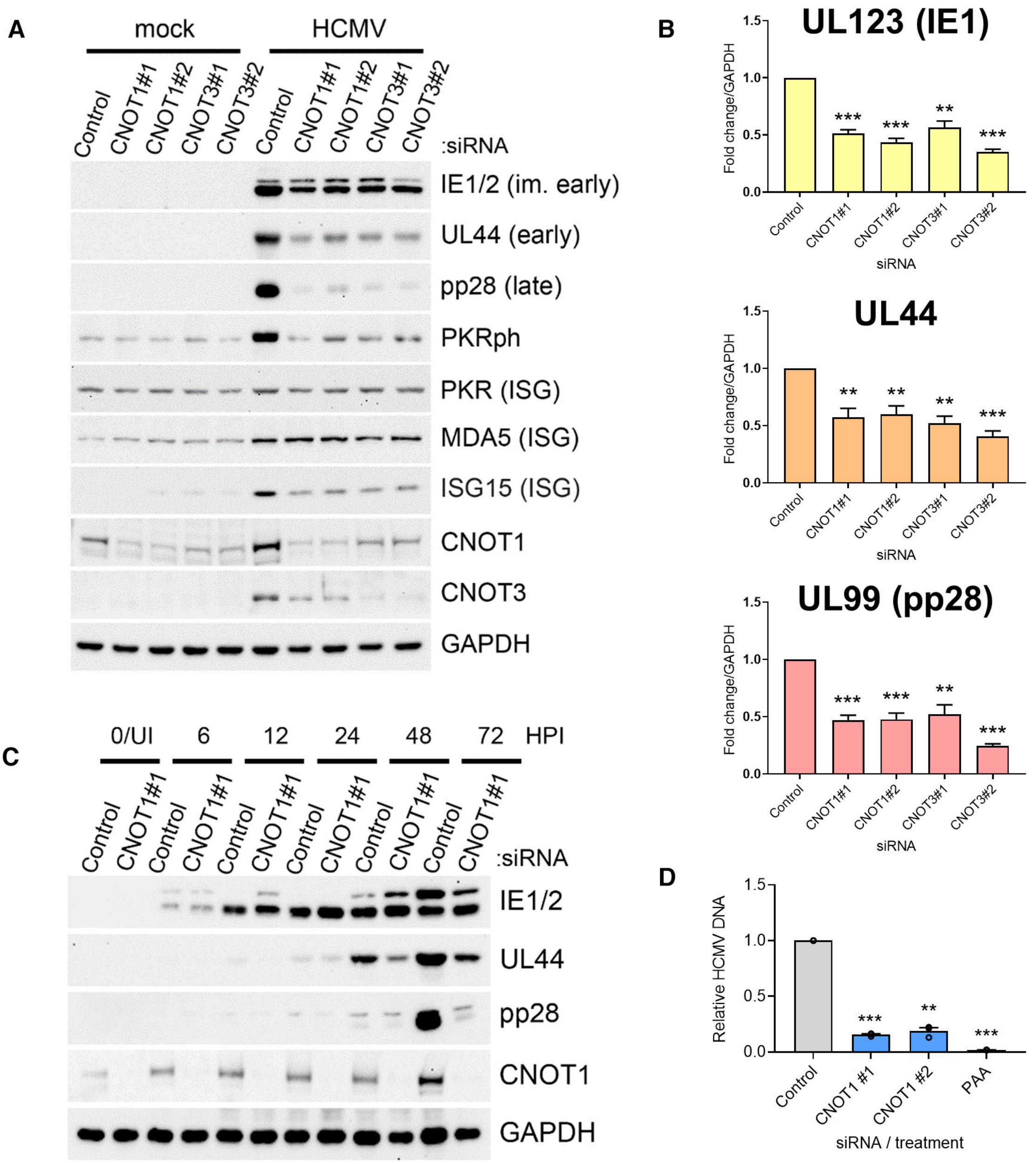

Figure 2. CNOT1 and CNOT3 promote viral gene expression late in HCMV infection.

A Immunoblot analysis of lysates from NHDF cells transfected with the indicated siRNAs and infected with HCMV AD169 at MOI = 3, collected at 72 HPI. Lysates were probed for representative viral proteins from each temporal expression class, select ISGs (MDA5, ISG15, PKR) and activated T446-phosphorylated PKR (PKRph) and GAPDH.

B RT-qPCR analysis for viral mRNAs from RNA isolated from cells treated and infected as in (A). Protein names, where different from gene names, indicated in brackets. Mean ± SEM (n = 6 biological replicates) is plotted with statistical significance established by ANOVA test with Dunnett multiple comparison correction compared with control siRNA; (**) $P < 0.002$, (***) $P < 0.001$.

C Immunoblot analysis of lysates from NHDF cells transfected with control or CNOT1 siRNAs and infected with HCMV AD169 at MOI = 3 collected at the indicated times post infection. Lysates were probed for representative viral proteins indicated, CNOT1 to confirm knockdown and a cellular loading control (GAPDH).

D NHDFs transfected with control or CNOT1-specific siRNAs were infected with HCMV AD169 at MOI = 3, as in (A). At 72 hpi, total DNA was collected and HCMV DNA abundance was quantified by qPCR. Untransfected cells were treated in parallel with viral DNA polymerase inhibitor PAA after virus inoculation as a control. Mean ± SEM (n = 3 biological replicates) is plotted with statistical significance established by ANOVA test with Dunnett multiple comparison correction compared with control siRNA; (**) $P < 0.002$, (***) $P < 0.001$.

Source data are available online for this figure.

 © 2023 The Authors

significant reduction of at least 50% of each transcript in CNOT1 and CNOT3 knockdown cells compared with nonsilencing control siRNA transfected cells (Fig 2B).

Immunoblotting for CNOT1 and CNOT3 confirmed protein depletion by gene-specific siRNAs and also hinted at increased levels of CNOT1 and CNOT3 proteins in HCMV-infected cells (Fig 2A). A reduction in CNOT1 protein levels in CNOT3-specific siRNA treated cells and reduced accumulation of CNOT3 in infected cells treated with CNOT1-specific siRNAs was also noted, implying each knockdown in fact represents a reduction in the level of both proteins to differing degrees. This pattern was not observed at the mRNA level by RT-qPCR (Fig EV2A), suggesting either a mode of translational control or protein stabilization coordinates this coregulation. Cooperative stability of protein complex components is a common mechanism to maintain appropriate subunit stoichiometry and coregulation of CNOT7 and CNOT8 has been reported (Hsu *et al*, 2022; Stoney *et al*, 2022).

A time course of infection demonstrated impaired accumulation of UL44 in CNOT1-depleted cells compared to control cells from 48 HPI (Fig 2C). Again, the largest deficit observed was in levels of the late protein pp28 at 72 HPI and analysis of viral transcript levels confirmed impaired gene expression mostly limited to this late timepoint (Figs 2C and EV2B). By contrast, while only a modest reduction of IE1 and IE2 protein accumulation was observed at 72 HPI in CNOT1-depleted cells (Fig 2C), none was observed at 6HPI, consistent with equivalent viral entry to knockdown cells. This was directly tested by immunofluorescence staining for IE1/2 expression at 6HPI, where no significant differences in IE1/2-positivity were observed in CNOT1- or CNOT3-depleted cells (Fig EV2C). For both UL44 and pp28, deficits in protein levels in CNOT1-depleted cells appeared to exceed deficits in mRNA levels (Figs 2C and EV2B) suggestive of impaired viral translation. Very similar temporal effects on viral protein accumulation were observed over a time course of infection of CNOT3-depleted cells (Fig EV2D).

A failure to fully activate pp28 expression raised the possibility that CNOT1 depletion may interfere with viral DNA synthesis, which is required for expression of late gene products such as pp28. Indeed, viral DNA levels were reduced by more than 80% by each CNOT1-specific siRNA (Fig 2D) consistent with the notion that CCR4-NOT function is required for viral DNA synthesis to be successfully completed.

Our previous work investigating the dependence of another large DNA virus, the prototypical poxvirus vaccinia (VACV), on a cellular mRNA decay enzyme, Xrn1, revealed hyper-activation of cell intrinsic antiviral responses including the dsRNA-activated protein kinase PKR upon Xrn1 depletion (Burgess & Mohr, 2015). However, accumulation of activated, phosphorylated PKR was not detected in CNOT1/3-depleted HCMV-infected samples; indeed, detection of phospho-PKR was reduced compared to control siRNA-transfected HCMV-infected samples (Fig 2A). Likewise, a global upregulation of antiviral interferon (IFN)-stimulated genes (ISGs) was previously found to explain impaired HCMV replication following disruption of the m^6A RNA modification pathway (Rubio *et al*, 2018), but this was not detected in CNOT1/3-depleted cells when representative ISG-encoded proteins (ISG15, MDA5, and PKR) were examined (Fig 2A). Together, this data indicates that CNOT1 and CNOT3 are required for HCMV gene expression and DNA replication and suggest a more nuanced mechanism than those by which other cellular RNA metabolism enzymes regulate DNA virus infections.

CCR4-NOT selectively regulates HCMV infection

We next asked whether regulation of infection by CCR4-NOT complex components was restricted to HCMV or extended to other DNA viruses. CNOT1 or CNOT3 depletion did not measurably impact the accumulation of VACV proteins, detected using a pan-VACV antibody, or the accumulation of IE, early and late HSV-1 proteins ICP4, US3, or gC following a low multiplicity infection (Fig 3A). By contrast, infection with clinical HCMV strain TB40/E was acutely sensitive to CNOT1 and CNOT3 depletion, displaying reduced accumulation of proteins representing each viral temporal gene class in knockdown cells (Fig 3B). Correspondingly, the effect of CNOT1 depletion on viral protein accumulation followed identical kinetics in TB40/E (Fig EV2E) compared to AD169 (Fig 2C) HCMV infection and released viral titers following a low MOI TB40/E infection were reduced by more than 10^4-fold (Fig EV2F). Together, these data indicate that the dependence of virus gene expression and replication upon CCR4-NOT components is not universally shared among DNA or herpes viruses, and the complex is important for lytic infections of both laboratory and clinical strains of HCMV.

Selective regulation of CCR4-NOT complex components upon HCMV infection

Genes associated with RNA processing and translation are among those differentially expressed during HCMV infection of fibroblasts and several upregulated host RBPs have been shown to be required for efficient replication (Perez *et al*, 2011; McKinney *et al*, 2012; Tirosh *et al*, 2015; Batra *et al*, 2016; Song *et al*, 2019). Further, our knockdown experiments hinted at HCMV-induced upregulation of CNOT1 and CNOT3 (Fig 2A). To establish whether CCR4-NOT complex components are altered in their abundance during HCMV infection, we monitored expression of nucleases CNOT6, 6L, 7, and 8 and nonenzymatic modules CNOT1, 2 3, by immunoblot where reliable antibodies could be sourced and RT-qPCR where not (Fig 4A and B). CNOT1 and CNOT3 proteins dramatically increased during infections with both virus strains beginning at 24 HPI (Fig 4A). By contrast, levels of CNOT2 protein only moderately increased. Interestingly, mRNA levels for CNOT1, 2, and 3 increased by a maximum of twofold compared to uninfected cells during infections (Fig 4B), suggestive of a translational modality to CNOT1 and CNOT3 protein upregulation, a feature previously reported for the regulation of CNOT3 expression in response to diet (Morita *et al*, 2011). Caf1 nuclease CNOT7 protein increased in abundance during infections with AD169 or TB40/E strains of HCMV (Fig 4A) and expression of mRNAs encoding CCR4 nucleases CNOT6 and CNOT6L were also consistently upregulated 2–3-fold (Fig 4B). Differences in the timing of upregulation of CNOTs between strains may reflect viral gene expression differences, indeed AD169 lacks entire genomic loci (Wilkinson *et al*, 2015). Greater abundance of scaffold protein CNOT1 and nucleases are likely to increase the functional availability of the entire complex during infection. Incorporation of CNOT3 into the complex can influence nuclease activity (Raisch *et al*, 2019), and an orthologous component in yeast (Not5) interacts with ribosomes to link translational inefficiency with deadenylation

© 2023 The Authors

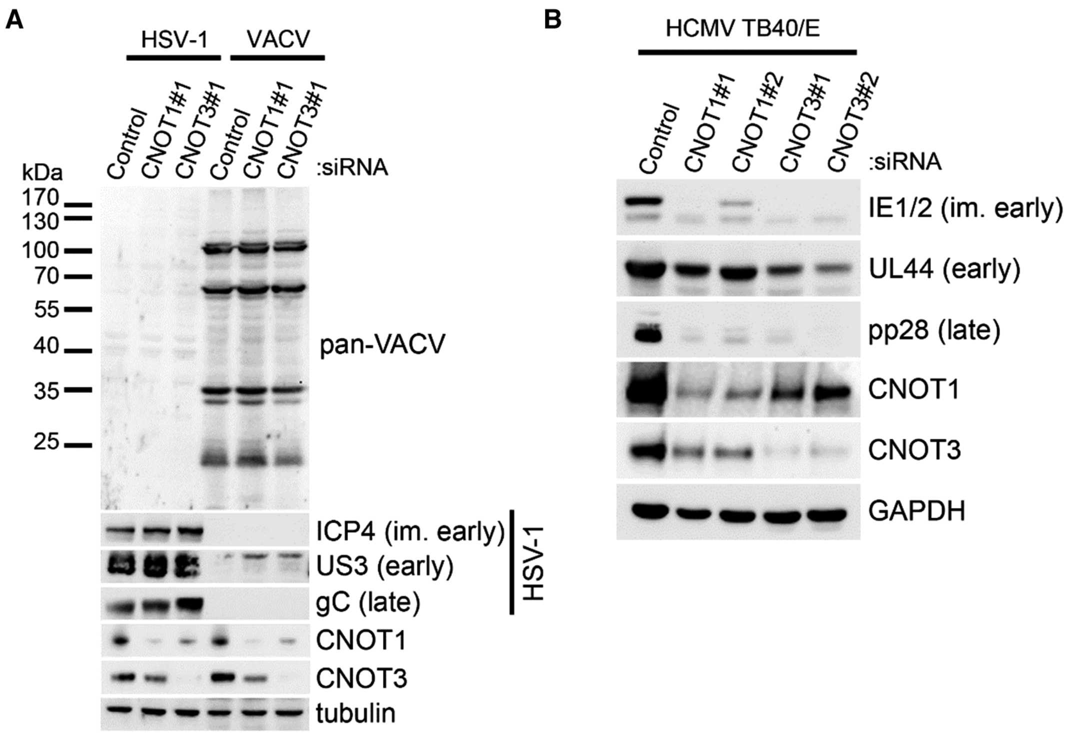

Figure 3. CNOT1/3 dependence is specific to HCMV and consistent between strains.

A Immunoblot analysis of lysates from NHDF cells transfected with the indicated siRNAs and infected with vaccinia virus (VACV) or Herpes Simplex Virus 1 (HSV-1) at MOI = 0.005. Following multicycle infection, lysates were collected at 72 HPI and probed for representative HSV-1 proteins from each temporal expression class (ICP4, US3, gC), for vaccinia proteins using a pan-vaccinia antibody, and for tubulin as a loading control.

B Immunoblot analysis of lysates from NHDF cells transfected with the indicated siRNAs and infected with HCMV clinical strain TB40/E at MOI = 3, collected at 72 HPI. Lysates were probed for representative viral proteins from each temporal expression class and GAPDH.

Source data are available online for this figure.

(Buschauer *et al*, 2020). Thus, changes in the abundance of this non-nuclease component may have additional complex effects on mRNA targeting or the deadenylation activity of CCR4-NOT during infection.

CCR4-NOT broadly regulates viral gene expression and influences host responses

To further understand the mechanism whereby CNOT1 and CNOT3 regulate HCMV productive replication, we performed short-read RNA sequencing (RNA-Seq) of control and CNOT1 and CNOT3-depleted cells at 72 HPI, the timepoint at which we established the most prominent effects on viral gene expression occur (Figs 2C and EV2B). Comparison of significantly (adjusted P value [Padj] < 0.05) host differentially expressed genes (DEGs) following CNOT1 (n = 2,463) or CNOT3 (n = 1,481) depletion demonstrated significant overlap (Fig 5A) and highly correlated gene expression changes among the intersecting gene set, R^2 = 0.847 (Pearson; Fig 5B, Dataset EV1). Nonoverlapping DEGs could represent those that failed to meet the significance threshold in either knockdown condition or indicate CNOT1/3-specific regulation. Surprisingly given the targeting of mRNA decay machinery, a similar proportion of positive and negative gene expression changes were represented within this set, indicating indirect effects of dysregulated host or viral genes may also influence host gene expression in CNOT1/3 knockdown cells.

Consistent with our RT-qPCR data, the proportion of reads aligning to the viral genome were significantly reduced in CNOT1- and CNOT3-depleted samples relative to samples treated with the nonsilencing control (Fig 5C). The reduction in viral reads upon CNOT1 or CNOT3-depletion extended to many genomic regions, inclusive of the UL82/UL83 transcriptional unit which encodes tegument proteins pp71 and pp65, important for viral gene expression and innate immune evasion (Fig 5D). Directly comparing the distribution of viral reads (50,000, randomly sampled) across the viral genome, we observe very few regions with markedly (> 2-fold) different coverage in CNOT1- or CNOT3-depleted cells relative to control siRNA transfected cells (Fig EV3). These data indicate that targeting of either CNOT1 or CNOT3 components of the CCR4-NOT complex limits HCMV replication by a common molecular mechanism that results in a broad downregulation of viral gene expression.

Analysis of cellular pathways associated with the set of genes differentially expressed by either CNOT1 or CNOT3 depletion (n = 3,108) using Reactome (Jassal *et al*, 2020) showed downregulation of genes associated with type I (alpha/beta) IFN signaling (Fig 5E), consistent with the immunoblotting of representative ISG-encoded proteins (Fig 2A), each of whose expression was

 © 2023 The Authors

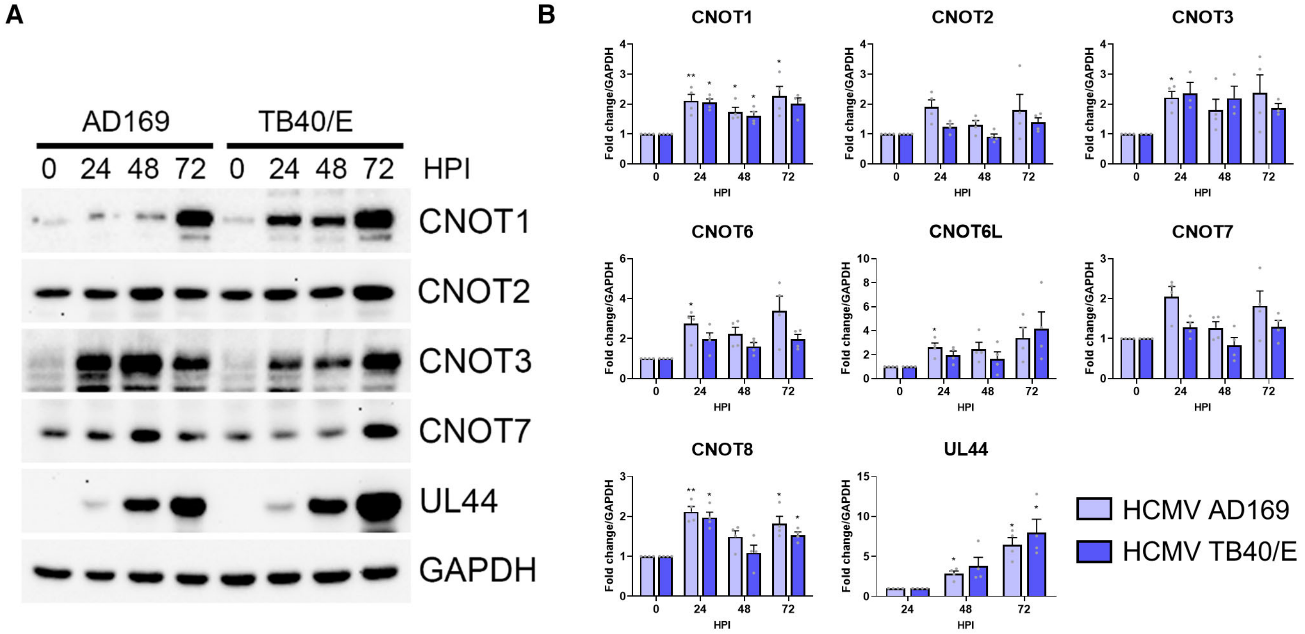

Figure 4. Selective expression changes of CCR4-NOT components upon HCMV infection.

A Immunoblot analysis of lysates from NHDF cells infected with HCMV lab strain AD169 or clinical strain TB40/E at MOI = 3, collected at the indicated hours post infection. Lysates were probed for CCR4-NOT components, viral protein UL44, and GAPDH.

B RT-qPCR analysis for mRNAs encoding CCR4-NOT components from RNA isolated from cells infected as in (A). Mean fold changes relative to GAPDH ± SEM (n = 4 biological replicates) are plotted with statistical significance established by ANOVA test with Dunnett multiple comparison correction compared with 0HPI (or 24HPI for UL44); (*) $P < 0.033$, (**) $P < 0.002$, no asterisk: not significant.

Source data are available online for this figure.

downregulated at the transcript level. Furthermore, genes associated with IFN gamma signaling were also downregulated, excluding suppression of either of these paracrine antiviral pathways as an explanation for HCMV's requirement for CNOT1/3. The major histocompatibility complex (MHC) class I antigen processing and presentation pathway was also represented among downregulated genes. Upregulated pathways were also enriched for cytokines, comprising select proinflammatory interleukin pathways IL-12 (FDR = 3.84e^{-08}), IL-4, and IL-13 (FDR = 1.51e^{-05}) as well as anti-inflammatory mediator IL-10 (FDR = 1.08e^{-06}) (Fig 5E) though the cytokines themselves were not represented within the lists of genes that contributed to these pathways (Table EV1). As IL-12 and IL-4 are generally associated with immune cell modulatory functions (Vignali & Kuchroo, 2012; Keegan *et al*, 2021), their ability to affect

Figure 5. CNOT1 and CNOT3 depletion results in consistent host and viral transcriptome changes.

NHDFs were transfected with nonsilencing control siRNA, siCNOT1 (#1) or siCNOT3 (#1) and infected with HCMV AD169 at MOI = 3. At 72HPI RNA was isolated for Illumina RNA sequencing and transcriptomic analysis.

A Venn diagram showing the number of significantly (Padj < 0.05) differentially regulated genes in the comparisons between control siRNA- and either siCNOT1- or siCNOT3-treated cells.

B Scatter plot depicting the $\log_2$ fold change vs. control for the 836 intersecting genes in the siCNOT1 and siCNOT3 datasets.

C Proportions of sequencing reads aligning to the HCMV strain AD169 reference genome (FJ527563.1) in samples treated with either nonsilencing control, CNOT1 or CNOT3 siRNAs plotted as mean ± SD from three biological replicates. (***) $P < 0.001$ by unpaired two-tailed t-test.

D RNA-Seq coverage plots across the HCMV AD169 genome for nonsilencing control siRNA (siCTRL), siCNOT1, and siCNOT3 datasets. Each plot shows a representative sample of the three biological replicates with depth of coverage plotted on the y-axis and genome position on the x-axis. Canonical HCMV ORFs are shown as gray boxes.

E Pathway analysis of the 836 intersecting genes using the Reactome Pathway Browser (Jassal *et al*, 2020). Upregulated (n = 489) and downregulated (n = 347) were processed separately.

F mRNA decay of DEGs was monitored over 8 h actinomycin D treatment in control, CNOT1 (#1) and CNOT3 (#1) siRNA-treated cells, normalized to GAPDH. Representative experiments of three biological replicates are shown.

G The effect of DEG-depletion by siRNA on released infectious viral titer was determined by TCID50 from culture supernatants following 7d infection with AD169 at low MOI (0.05) of knockdown cells, plotted as the mean ± SEM (n = 3 biological replicates). No significant differences between control and DEG knockdowns were found by ANOVA test with Dunnett multiple comparison correction.

Source data are available online for this figure.

© 2023 The Authors

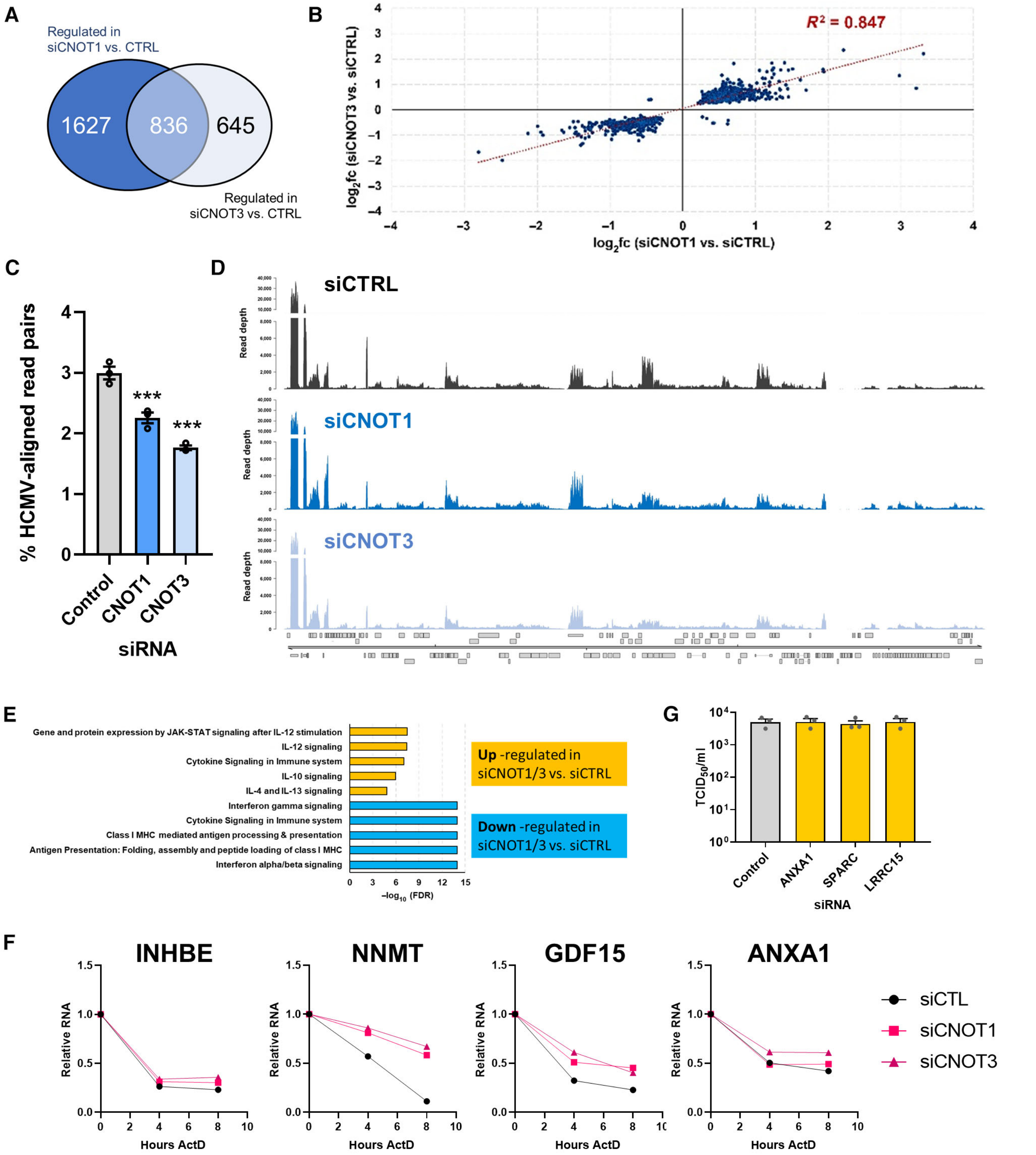

Figure 5.

HCMV productive replication in fibroblasts was evaluated. Pretreatment of NHDFs with up to 200 ng/ml IL-4 or IL-12 did not significantly affect virus spread, in contrast to the marked (65%) reduction in virus-infected cells observed following treatment with human IFN-beta (Fig EV4A). Though this data indicates these pathways are not important in this context, their dysregulation and regulation of MHC could, however, contribute to CCR4-NOT regulation of HCMV in a natural human infection.

We next looked more closely at the individual genes that were strongly (> 2 fold) and significantly (Padj < 0.05) up- (25 genes) or down- (7 genes) regulated in both CNOT1- or CNOT3-depleted cells relative to control siRNA-treated cells (Table EV2) and validated the

© 2023 The Authors

changes in several by RT-qPCR (Fig EV4B). All 15 upregulated genes tested were upregulated in uninfected CNOT1-knockdown cells in addition to HCMV-infected CNOT1-knockdown cells, indicating their dysregulation was not dependent on changes to viral gene expression. To test whether the upregulation of these genes can be explained by mRNA stabilization, we monitored the decay of select transcripts during transcriptional inhibition by actinomycin D (Fig 5F). INHBE, NNMT, GDF15, and ANXA1 each displayed reduced decay in both CNOT1 and CNOT3-knockdown cells compared to control cells, indicating that altered CCR4-NOT-mediated deadenylation directly contributes to host gene dysregulation in knockdown cells.

We cross-referenced the strongly and significantly dysregulated gene set with the results of a recent pooled CRISPR-interference genome-wide screen of host factors for HCMV, which infers pro- or antiviral function of host genes by the positive or negative survival ratio, respectively, of cells in which they are disrupted after infection (Hein & Weissman, 2022; Table EV2). Among the genes upregulated were four with potentially anti-HCMV activity ($\log_2$ survival ratio ≤ -0.2)—serine protease 8 (PRSS8), leucine-rich repeat containing 15 (LRRC15), growth differentiation factor 15 (GDF15), and annexin A1 (ANXA1). Chemokines CCL7, CXCL3, and CXCL6 and inflammatory cytokines IL-1B and IL-6 that were also upregulated in CNOT1/3 knockdown infected cells (Tables EV2 and EV4B) were not, however, implicated as antiviral in Hein and Weissman's screen (Hein & Weissman, 2022). This is perhaps explained by HCMV's manipulation of the activities of IL-1B and IL-6 and the absence of immune cells regulated by these chemokines in their experiment design (Geist & Dai, 1996; Yang *et al*, 2002; Reitsma *et al*, 2013; Harwardt *et al*, 2016), conditions analogous to ours. We tested the ability of dysregulated host genes with the highest antiviral potential (LRRC15, ANXA1) and secreted protein acidic and rich in cysteine (SPARC), another upregulated gene with lower predicted antiviral activity, to regulate HCMV replication using RNAi. Each gene was effectively targeted by its respective siRNA (Fig EV4C), but differences in infectious virus production under these conditions were not detected following any individual knockdown (Fig 5G), suggesting that the contribution of host factor dysregulation to the CNOT1/3 phenotype is likely to be multigenic.

Overall, we find evidence of a dysregulated host response in CNOT1 and CNOT3-depleted HCMV-infected cells, which is not limited to a broad stabilization of transcripts, and that collective dysregulation of many host genes likely contributes to impaired viral replication.

Chemical inhibition of CCR4-NOT nuclease activity limits HCMV spread

In our focused RNAi screen, we did not detect large or consistent reductions in HCMV spread when the nuclease components of CCR4-NOT were targeted, and co-depletion also did not affect viral replication (Fig EV1D). As the stoichiometry of CCR4-NOT nucleases to available CNOT1 scaffolds is unknown in mammalian cells, without full genetic deletions residual nuclease incorporation into CCR4-NOT and activity may remain. To directly test whether targeting CCR4-NOT deadenylation activity can impact HCMV replication, we used a purine-2,6-dione derived inhibitor that selectively blocks the activity of Caf1 (compound 8j, Jadhav *et al*, 2015), treating cells after the initial inoculation period and for the remaining duration of the 7 day multicycle infection. Quantification of infected cell number revealed significant reductions following treatment with Caf1 inhibitor (Caf1i), compared to DMSO control at 100 μM (68%) and 75 μM (33%) with limited effects on cell viability (Fig 6A and B). As in our initial screen, the observed changes in spread resulted in an amplified impact on released virus titer, with an 85-fold reduction observed following 100 μM Caf1i treatment (Fig 6C). This demonstrates that a selective inhibitor of CCR4-NOT nuclease activity can limit HCMV replication. We therefore conclude that the CCR4-NOT complex regulates HCMV infection by its mRNA deadenylation function.

Viral mRNA poly(a)-tails are differentially affected by CNOT1 disruption

As depletion of CNOT1 and CNOT3 limits efficient HCMV replication (Fig 1) and leads to consistent changes in host and viral gene expression with evidence for host mRNA stabilization (Fig 3), we next directly investigated whether knockdowns of these nonenzymatic components impact the deadenylase activity of the CCR4-NOT complex. To measure poly(A)-tail lengths of host and viral mRNAs, we applied direct RNA sequencing (DRS) using the Oxford Nanopore Minion platform. Analysis was performed using nanopolish and revealed a distribution of poly(A)-tail lengths on host mRNAs in uninfected cells similar to that reported in other DRS studies (Workman *et al*, 2019), with a median length, indicated by a red dot, of 86 nt, and a mode of 56 nt (Fig 6D, Dataset EV2). While the modal length did not appreciably change during infection, the distribution shifted upward with the median tail length increasing to 105 nt at 24 HPI before gradually decreasing to near uninfected length (91 nt) by 72 HPI. By contrast, poly(A) tails on viral mRNAs bore a markedly different length distribution, with a median of 127 nt at 24 HPI which more closely reflected the modal length (136 nt) and decreased only slightly during infection (Fig 6D). Longer poly(A)-tails on HCMV transcripts will permit binding of additional PABPC molecules, expected to promote the stability and translation of mRNAs, and is concordant with the upregulation of and dependence on PABPC1 in HCMV infection (Walsh *et al*, 2005; Perez *et al*, 2011; McKinney *et al*, 2012). Consistent with the prevailing model that transcripts are first issued with a ~ 250 nt poly(A)-tail which is trimmed upon cytoplasmic export (Eckmann *et al*, 2011; Passmore & Coller, 2022), HCMV long noncoding RNA 4.9, which is retained in the nucleus and promotes viral DNA replication (Tai-Schmiedel *et al*, 2020), bore a much longer tail than typical protein coding viral transcripts (mean length 226 nt vs. 119 nt of US22A at 24 HPI) (Fig 6E).

We tested the effect of siRNA targeting of CNOT1 on poly(A)-tail lengths at 72 HPI, conducting two independent experiments and measuring the tail lengths of more than 100,000 transcripts in each condition per experiment. The effect of CNOT1 knockdown on host mRNA poly(A)-tails was stark, resulting in an obvious upward shift in overall tail length distribution and an increase in median tail length to 119 nt, a net gain of 35 nt, confirming disruption of CCR4-NOT deadenylase activity (Fig 6F). The modal length of cellular mRNA poly(A)-tails increased remarkably upon CNOT1 knockdown to 131 nt from 55 nt (Fig 6F, Dataset EV2). By contrast the impact

© 2023 The Authors

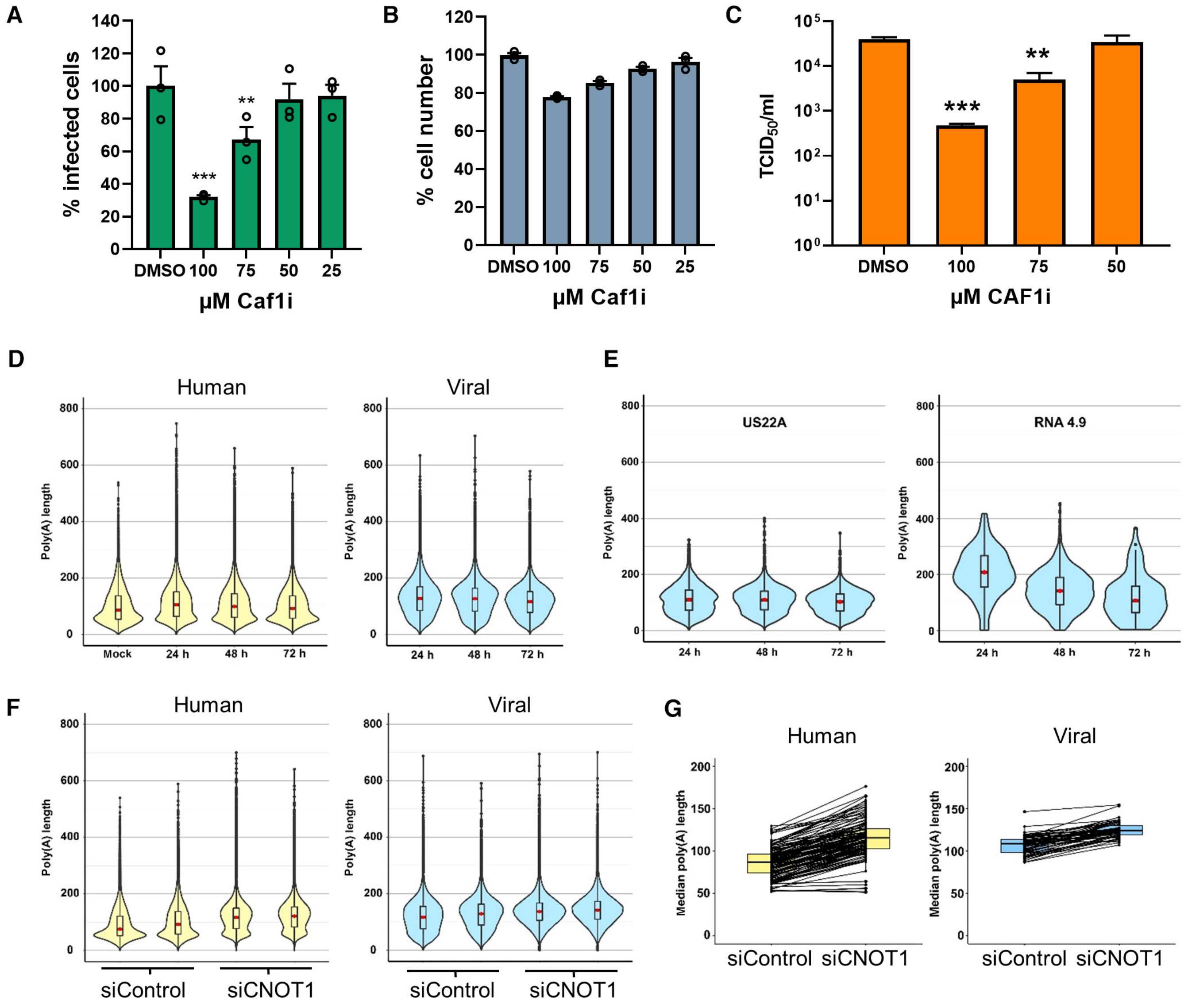

Figure 6. Caf1i (CNOT7/8) inhibitor has anti-viral activity against HCMV and CCR4-NOT disruption selectively impacts host mRNA poly(A)-tails during HCMV infection.

A NHDFs infected with HCMV (AD169) at low MOI (0.05) for 1 h and subsequently incubated for 7 days in the presence of Caf1 inhibitor (Caf1i) or vehicle control (DMSO) equivalent to the highest treatment concentration. Infected cells were identified as in Fig 1A using high content imaging. Mean % infected cells ± SEM, normalized to DMSO-treated cells (n = 3 biological replicates), is plotted. Statistical significance was established by ANOVA test with Dunnett multiple comparison correction compared with DMSO sample, (**) $P < 0.02$, (***) $P < 0.001$, no asterisk: not significant.

B NHDF cell viability following 7 days Caf1i treatment was assessed by quantifying cell number by DAPI nuclear staining and high content imaging. Mean cell number (± SEM) normalized to DMSO-treated cells (n = 3 biological replicates) is shown.

C Infectious viral titers from culture supernatants following Caf1i treatment were determined by TCID50, plotted as the mean ± SEM (n = 6 biological replicates). Statistical significance was established by ANOVA test with Dunnett multiple comparison correction compared with DMSO sample, (**) $P < 0.02$, (***) $P < 0.001$, no asterisk: not significant.

D Poly(A) tail length distributions on host (left, yellow) and viral (right, blue) RNAs from nanopore direct RNA sequencing of mock infected and HCMV infected (24, 48, 72 HPI) NHDFs using Nanopolish.

E Poly(A) tail length distributions of individual HCMV transcripts.

F Poly(A) tail length distributions for host and viral RNAs obtained from HCMV-infected NHDFs (TB40/E MOI = 3, 72 HPI) treated prior to infection with either a non-silencing control siRNA or CNOT1 targeting siRNA (#1). Results from two independent experiments are shown adjacently.

G Median poly(A) tail lengths of each host and viral mRNA in (F).

Source data are available online for this figure.

on viral mRNA poly(A) tails was much less marked, with an increase in median tail length to 138 nt representing a net gain of 16nts, and little change to their modal length. The differential response to CNOT1-depletion was especially evident when the median tail length of every individual measured host and viral mRNA was plotted (Fig 6G).

 © 2023 The Authors

DRS permits identification of the specific mRNAs whose poly(A)-tails are most affected by infection and CNOT1 disruption, albeit within the constraints of read depth limitations and bias toward abundant transcripts imposed on both sample sets. The ability of the poly(A)-tail to promote stability and translation is dependent on PABPC proteins which require a minimum of 12 adenosines to bind (Kühn & Wahle, 2004) and occupy a footprint of ~ 27nts (Baer & Kornberg, 1983). We thus compared host transcripts that gained 20 nt or more in poly(A)-tail length and are consequently likely to experience a functional benefit, following infection (53%, 160/306 transcripts) with CNOT1 depletion (61%, 379/621 transcripts). This revealed a high proportion of overlap (91 transcripts; Fig EV5A), indicating that CNOT1-responsive transcripts are among those whose poly(A) status is changed in infection, consistent with the possibility that altered CNOT1 targeting and/or activity occurs.

Of the genes strongly and significantly upregulated by CNOT1 and CNOT3 knockdown (Table EV2), only two, SPARC and ANXA1, were detected by DRS. These mRNAs gained 55 and 20 nt, respectively, in CNOT1 knockdown cells, consistent with the observed stabilization of ANXA1 mRNA (Fig 5F). When we compared the poly(A)-tail length changes of all cellular transcripts measured by DRS and the expression changes detected by short-read RNA sequencing in CNOT1-depleted HCMV-infected cells (Fig EV5B), we did not, however, identify an overall correlation. As only the most abundant mRNAs are identified by DRS, the large steady state pool of these transcripts likely obscures the impact of stability changes associated with poly(A)-tail length alterations, which may be further obfuscated by transcription rate changes.

Together, these data show that deadenylase activity within the infected cell is measurably impacted by CNOT1 depletion, and though HCMV mRNAs are substrates of CCR4-NOT they unexpectedly appear to be targeted for deadenylation to a much lesser degree than host mRNAs, indicating it is the action of CCR4-NOT upon host mRNAs that is required by the virus. Moreover, the substantial and global extension of host poly(A)-tails in CNOT1-depleted cells may obviate the translational advantage viral mRNAs with long poly(A)-tails usually enjoy and contribute to impaired viral gene expression (Fig 7).

Discussion

In this study, we identify a novel host function on which HCMV infection is dependent—CCR4-NOT-dependent mRNA deadenylation. Depleting scaffolding components of the CCR4-NOT complex selectively reduced accumulation of HCMV RNA and proteins late in infection and impaired viral DNA synthesis, (Fig 2) but did not detectably impair replication of HSV-1, a related herpesvirus

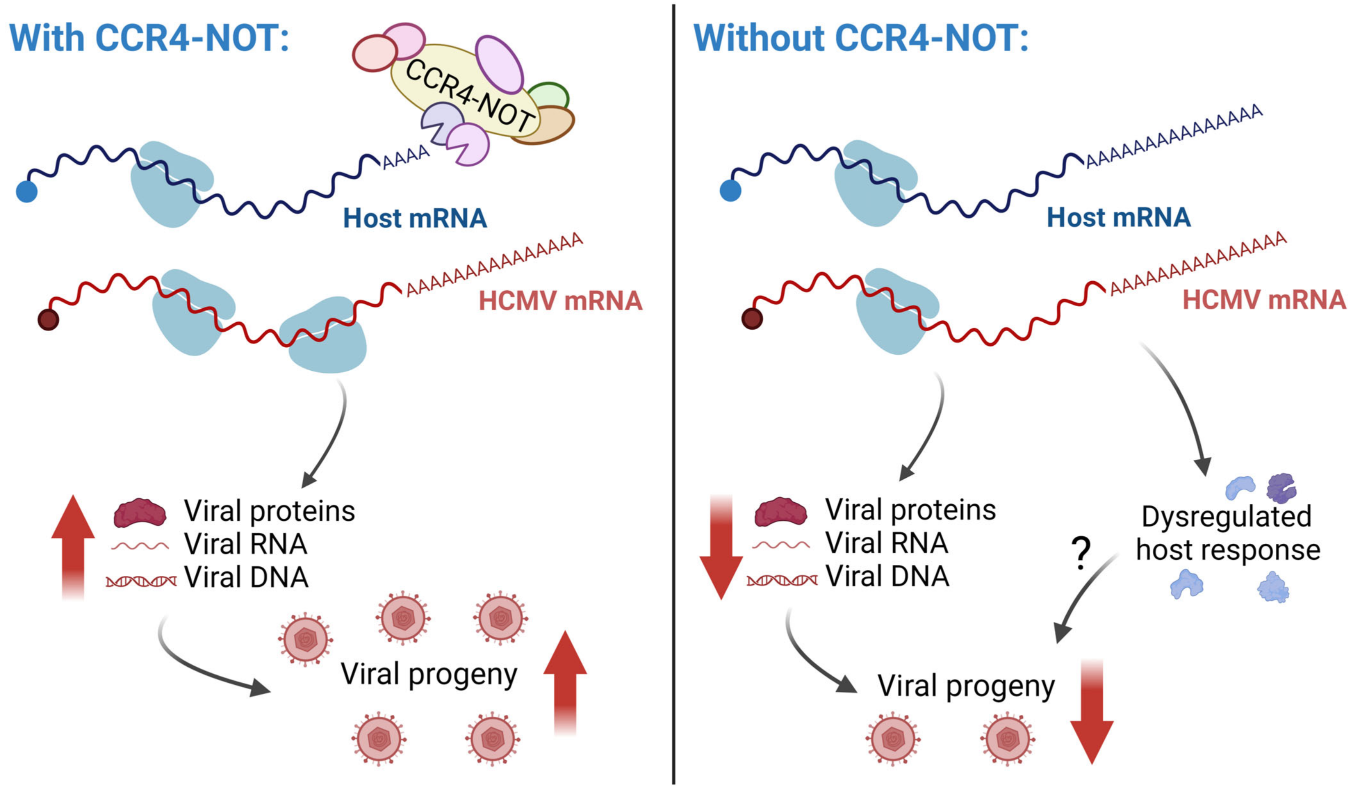

Figure 7. Model for CCR4-NOT regulation of HCMV infection.

In normal cells, CCR4-NOT maintains shorter poly(A)-tail lengths on cellular mRNAs, while HCMV mRNAs bear longer tails and are translated with high efficiency. In CCR4-NOT-depleted cells, cellular mRNA poly(A)-tail lengths are extended and reduced viral gene expression, genome synthesis and progeny release occurs. Dysregulation of host gene expression is also detected, but its importance to altered viral replication is unclear.

© 2023 The Authors

subfamily member, or VACV (Fig 3A). Paradoxically, however, HCMV poly(A)-tails are relatively insensitive to CCR4-NOT, as their lengths are little changed by CCR4-NOT disruption (Fig 6F). The ability of a chemical inhibitor of a CCR4-NOT nuclease to also impact viral replication indicates it is nonetheless the deadenylase function of the complex that is needed for HCMV productive growth (Fig 6A and C).

The selective requirement of HCMV for CCR4-NOT complex subunit CNOT1 (Fig 1B) likely reflects a better impairment of complex function by targeting the complex scaffold than functionally redundant nuclease paralogs. Though CNOT3 interaction with the complex is not necessary for stable nuclease incorporation (Boland *et al*, 2013), we noted a reduction in CNOT1 protein levels when CNOT3 was depleted (Fig 2A). This is in agreement with a previous study, which also found that knockdown of CNOT1 and CNOT3 extended the half-life of a reporter mRNA to a similar degree (Boland *et al*, 2013). In a recent pooled CRISPR-interference genome-wide screen of host factors for HCMV, CNOT1 and CNOT3 were also the only CCR4-NOT components that led to a strong enrichment of their respective targeting cassettes in the surviving cell pool indicative of viral dependence (Hein & Weissman, 2022), further supporting our results that these are the complex members specifically required by HCMV.

During infection of CNOT1-depleted cells, we detected impaired progression though the viral lifecycle, extension of host mRNA poly(A)-tails, and changes to the expression of host factors previously implicated in the regulation of HCMV infection (Fig 7). We tested the ability of single dysregulated host genes, prioritized by the magnitude of their differential expression and their prior characterization as anti-HCMV in a genome-wide screen (Hein & Weissman, 2022), to impact HCMV replication. This failed to identify an individual host gene responsible for the CNOT1/3 phenotype, perhaps highlighting a limitation to the predictive value of such screens or indicating that the contribution of altered host gene expression is through a collective dysregulation of multiple host factors, which could prove intractable to fully discern. Global changes to the poly(A)-RNA and RBP landscape in CCR4-NOT-disrupted cells also likely hamper the ability of HCMV to effectively commandeer the host cell translation apparatus. We found that in normal infection conditions HCMV mRNAs enjoy a significant tail-length advantage over host mRNAs providing a larger platform for binding of PABPC proteins, predicted to promote mRNA stability and translation. Indeed, ribosome profiling analysis has shown HCMV transcripts are more efficiently translated than host transcripts late in infection (Tirosh *et al*, 2015). By contrast, in CNOT1-depleted cells, the poly(A)-tails of host mRNAs are extended to a similar length to those of HCMV mRNAs, eliminating this tail-length advantage. Concordantly, we observe a greater impact on viral protein accumulation relative to their mRNA abundances in CNOT1-depleted cells consistent with impaired viral translation. A failure to compete for ribosome access may therefore explain an impaired accumulation of viral factors necessary to proceed through the HCMV lifecycle (Fig 7)—a hypothesis to be interrogated in future work.

How HCMV mRNA poly(A)-tails are so much longer than those of host mRNAs and resist CCR4-NOT deadenylation is yet to be fully explained. HCMV tails tend not to exceed the typical initial tail length specified by PABPN1 and CPSF interactions (~ 250 nt; Eckmann *et al*, 2011). As this machinery is also used by cellular transcripts whose tails are not dramatically affected by infection, it seems unlikely that viral mRNAs are issued with longer tails at "birth". Two mechanisms have been reported that promote HCMV poly(A)-tail length in the cytoplasm, though each acts in an mRNA-specific manner dictated by discrete RNA sequence motifs (Batra *et al*, 2016; Kim *et al*, 2020). Incorporation of non-A nucleotides into poly(A) tails by the TENT4–ZCCHC14 complex impairs deadenylation by CCR4-NOT and such non-As were identified in approximately 16% of viral RNAs, compared to around 11% of host mRNAs (Lim *et al*, 2018; Kim *et al*, 2020). Collectively HCMV poly(A)-tails were also shown to be sensitive to levels of CPEB1, a cytoplasmic RNA-BP upregulated in infection which recruits cytoplasmic poly(A)-polymerases to extend poly(A)-tails (Batra *et al*, 2016). CPEB1 was also found to bind select viral mRNAs (Batra *et al*, 2016); however, as the poly(A)-tails of many mammalian mRNAs are CPEB1-responsive (Shin *et al*, 2022), it is unclear how its activity could be broadly preferentially targeted to viral mRNAs to fully explain their outsized tails and CCR4-NOT resistance.

An open possibility is differential activity of the CCR4-NOT deadenylation complex toward HCMV mRNAs during infection. We detected alterations to the abundance of select CNOT proteins during infection (Fig 4A), which may alter complex function. HCMV protein UL38 is responsible for the translational upregulation of many cellular proteins during infection through activation of mTORC1 (McKinney *et al*, 2014), and it will be interesting to explore if this mechanism also controls CCR4-NOT. A recent HCMV protein interactome screen also found that the late protein UL72 interacts with the CCR4-NOT complex (Nobre *et al*, 2019) providing an opportunity for viral manipulation of its function. Conservation of the interaction in murine cytomegalovirus infers its importance (Gopal *et al*, 2018). Though viral subversion of poly(A)-tail homeostasis in general is underinvestigated, CCR4-NOT has been shown to be co-opted and relocalized by a plant negative-strand RNA virus to degrade specific viral RNAs and facilitate genome replication (Zhang *et al*, 2020). Adenovirus infection also results in degradation of select CCR4-NOT components favoring replication (Chalabi Hagkarim *et al*, 2018).

Our study adds another layer of understanding to HCMV's complicated relationship with mRNA poly(A)-tail control and viral commandeering of the translation apparatus in the infected cell, introducing a new key player—CCR4-NOT. While its importance is clear, further work is required to understand how CCR4-NOT's roles qualitatively regulating the host cell environment/response and enforcing a favorable translation landscape contribute to HCMV regulation. Future work must also address how poly(A)-tails of HCMV mRNAs resist CCR4-NOT and maintain their long lengths and demonstrate whether they do indeed confer greater stability and translational efficiency. Combining new approaches and sequencing technologies to assess both stability and poly(A)-tail length in concert would elegantly address these possibilities. The extensive interactions between HCMV and its host cell RNA turnover and translation pathways continue to reveal novel facets of cellular control networks, as well as vulnerabilities in the viral lifecycle ripe for therapeutic exploitation.

© 2023 The Authors

Materials and Methods

Reagents and Tools table

Reagent/Resource	Reference or source	Identifier or catalog number
**Experimental models**		
Normal Human Dermal Fibroblasts (NHDFs)	Lonza	CC-2511
**Antibodies**		
IE1/2, mouse monoclonal	Millipore	Mab810R
UL44, mouse monoclonal	Virus Sys	CA006
pp28, mouse monoclonal	Virus Sys	CA004-100
PKR phospho (Thr446), rabbit monoclonal	Abcam	ab32036
PKR, rabbit polyclonal	Proteintech	18244-1
MDA5, rabbit polyclonal	Proteintech	21775-1-AP
ISG15, rabbit polyclonal	Proteintech	15981-1-AP
Mx1, rabbit polyclonal	Proteintech	13750-1-AP
CNOT1, rabbit polyclonal	Proteintech	14276-1-AP
CNOT2, rabbti polyclonal	Novus	NBP2-56034
CNOT3, rabbit polyclonal	Proteintech	11135-1-AP
CNOT7, rabbit monclonal	Abcam	ab195587
VACV, rabbit polyclonal	Virostat	8101
ICP4, mouse monoclonal	Abcam	ab6514
US3	Gift of B. Roizman	
gC, mouse monoclonal	Abcam	ab6509
GAPDH, rabbit monoclonal	Cell Signaling	2118
Tubulin (beta), rabbit monoclonal	Cell Signaling	2128
Anti-mouse AlexaFluor 647	Fisher Scientific	A32787
Anti-mouse HRP	Fisher Scientific	45-000-679
Anti-rabbit HRP	Fisher Scientific	NA934
**Oligonucleotides and sequence-based reagents**		
RT-QPCR PRIMERS:		
GAPDH-F	TCTTTTGCGTCGCCAGCCGA	
GAPDH-R	ACCAGGCGCCCAATACGACC	
PARN-F	CGCAACAATAGTTTTACAGC	
PARN-R	AGAAAGCTCCTTCTTCATTC	
PAN2-F	CTATCATCTCTCAGTCAGGG	
PAN2-R	TTCACATGAAAGATATCGGC	
PAN3-F	TACAGACACCAAATCCTACTG	
PAN3-R	GTGAGAGAAAACTTGAGAGAAG	
CNOT1-F	CAGAGATTTTCCCCAAGAAC	
CNOT1-R	GATCCTGTCCATTAGAATGTC	
CNOT2-F	AGGAAAGTAGCTAAGGAGTTC	
CNOT2-F	TTTTTAGAAGGCTTGCTGAG	
CNOT3-F	GATGAGATCTTCAACCAGTC	
CNOT3-R	ATCATCTTCAGAGTTTTCCG	
CNOT6-F	TTGTCAGGTACTGCAAAAAG	
CNOT6-R	CTTGTCCTATCTGGTTCTTG	

Reagents and Tools table (continued)

Reagent/Resource	Reference or source	Identifier or catalog number
CNOT6L-F	CTATTTGGAGCAGGTATGAAG	
CNOT6L-R	CATCAGAATACTCTGGGTCC	
CNOT7-F	CTCTAACTTGCCTGAAGAAG	
CNOT7-R	TCCTGTAATCCACCTTTGAG	
CNOT8-F	AAGTTGCTTACAGATTCTCG	
CNOT8-R	TCCCTTAAGATTTTTGCAGC	
DCP1A-F	AAAGAATGATTCCAGCTTCC	
DCP1A-R	TTATTCTGCTCCAGTCATAGG	
DCP2-F	GAAAGAATCAAAGTATAGGGG	
DCP2-R	GAAGCCCATTTGTTCTTTTC	
EDC3-F	CAGGTTGAATCCCAAAAATG	
EDC3-R	GCTCATTGGTGATAGATTCC	
EDC4-F	CAACCATAAGAAACAAGACCC	
EDC4-R	CACCACATCTTTAACTCTCG	
DDX6-F	GTACTACGCATATGTAACTGAG	
DDX6-R	GATGTTCCTGCCTCATTTTAG	
LSM1-F	CCCTCCAGCAAGTATCCATT	
LSM1-R	TCGAGGAATGGAAAGACCTC	
EXOSC9-F	ACACTGTATCACCTGAAGAG	
EXOSC9-R	GAAAAAGGCAAAACTGACAC	
TTC37-F	GTTARRGTGAGGACAATCTCTG	
TTC37-R	AAAGTACTAGATCCTGAAGAGG	
WDR61-F	CAAGAAGGAAAACTCTGAGAC	
WDR61-R	GTCCCAAAGACGAATATGAG	
SKIV2L2-F	ACACTACATTTTTCCAGCAG	
SKIV2L2-R	GTCCTTTTGTTCCTCCTTTC	
IE1-F	CAAGTGACCGAGGATTGCAA	
IE1-R	CACCATGTCCACTCGAACCTT	
UL44-F	TTTTCTCACCGAGGAACCTTTC	
UL44-R	CCGCTGTTCCCGACGTAAT	
UL99-F	GTGTCCCATTCCCGACTCG	
UL99-R	TTCACAACGTCCACCCACC	
IL1B-F	CTAAACAGATGAAGTGCTCC	
IL1B-R	GGTCATTCTCCTGGAAGG	
CXCL3-F	CCTCAAGAACATCCAAGTG	
CXCL3-R	CCCCTTGTTCAGTATCTTTTC	
ANXA1-F	GGAACTGAAGAGAGATCTGG	
ANXA1-R	TCTTCATTCACACCAAAGTC	
LRRC15-F	CGTTGCTGTTCCAAGCGTCCAT	
LRRC15-R	GCTCAGTGGTAGAAGAGACGGA	
SPARC-F	AGTGAATACATTAACGGTGC	
SPARC-R	AATGTGTGTTTAAGGCAGAG	
NNMT-F	GTTTGGTTCTAGGCACTCTGCAG	
NNMT-R	AGAGCCGATGTCAATCAGCAGG	
GDF15-F	CGAAGACTCCAGATTCCG	

© 2023 The Authors

Reagents and Tools table (continued)

Reagent/Resource	Reference or source	Identifier or catalog number
GDF15-R	ACTTCTGGCGTGAGTATC	
NOV-F	GGAACCGTCAATGTGAGATGCTG	
NOV-F	GGCTTTGAGTGACTTCTTGGTGC	
TNFRSF11-F	GGTCTCCTGCTAACTCAGAAAGG	
TNFRSF11-R	CAGCAAACCTGAAGAATGCCTCC	
COL15A1-F	GGTGACACTGGTTTACCTGGCT	
COL15A1-R	GCCTTTCCAGAGGAATGTCCTC	
HMGA2-F	GAAGCCACTGGAGAAAAACGGC	
HMGA2-R	GGCAGACTCTTGTGAGGATGTC	
PTX3-F	CGAAATAGACAATGGACTCCATCC	
PTX3-R	CTCATCTGCGAGTTCTCCAGCA	
SERPINB4-F	CATGTTGATAGGTCAGGAAATG	
SERPINB4-R	ATTGATACGTCTTTTCTCCG	
siRNAs:		
control	Negative allstars cat #1027281	Qiagen
PARN #1	GAAAAGAAGGAGCGAUAUA	Dharmacon
PARN #2	CAUGAGAGGGCUUGCCGUA	Dharmacon
PAN2 #1	GACCUUGUUUGCUGGAUUA	Dharmacon
PAN2 #2	UCAAGGGUCUUUAUGAGAA	Dharmacon
PAN3 #1	AAAACAAGGUUGCGAGUAA	Dharmacon
PAN3 #2	CGACUUACUUCUAUACAGA	Dharmacon
CNOT1 #1	CUAUAAAGAGGGAACGAGA	Dharmacon
CNOT1 #2	GGCCAAAUUGUCUCGAAUA	Dharmacon
CNOT2 #1	CAUGAAUGGAGGAGACGUA	Dharmacon
CNOT2 #2	GGCAAGUUUAUACGGGCAA	Dharmacon
CNOT3 #1	GCACUAAGGCACAGUAUCU	Dharmacon
CNOT3 #2	AGACAUGGGUAGCGUCCAA	Dharmacon
CNOT6 #1	GAAAGAACGUGGCUAUAAU	Dharmacon
CNOT6 #2	GAGCACAGGUGGAGUAGAA	Dharmacon
CNOT6L #1	UGACAGCGCUGCACCUAAA	Dharmacon
CNOT6L #2	CCAAUUACACCUUUGAUUU	Dharmacon
CNOT7 #1	CAGCUAGGACUGACAUUUA	Dharmacon
CNOT7 #2	GGAGAAUUCAGGAGCAAUG	Dharmacon
CNOT8 #1	UUUCGUAGUUCCAUAGAUU	Dharmacon
CNOT8 #2	GAGAAUAGCCAGGUUAUCU	Dharmacon
DCP1A #1	Hs_DCP1A_6	Qiagen
DCP1A #2	Hs_DCP1A_7	Qiagen
DCP2 #1	GCUUGAAGGCACAACGUAA	Dharmacon
DCP2 #2	GUAUGGAGGUCUUGAGAAU	Dharmacon
EDC3 #1	CCUGAUAACAAACGCCUUA	Dharmacon
EDC3 #2	GGAGAUUGAUACCUAUGAA	Dharmacon
EDC4 #1	GGAUGGAGAUCGGCAUAAU	Dharmacon
EDC4 #2	GGACCAUGCCACCCAUUAA	Dharmacon
DDX6 #1	AGUAUGACCACCACUAUUA	Dharmacon
DDX6 #2	GAAGGACAAUAUACAAGCA	Dharmacon

© 2023 The Authors

Reagents and Tools table (continued)

Reagent/Resource	Reference or source	Identifier or catalog number
LSM1 #1	CGAGATGGAAGGACACTTATA	Qiagen
LSM1 #2	CAGCCTCATCGAGGACATTGA	Qiagen
XRN1 #1	AGAUGAACUUACCGUAGAAUU	Burgess & Mohr (2015)
XRN1 #2	CAGGTCGTAAATATCAAATAA	Qiagen/Burgess & Mohr, (2015)
EXOSC9 #1	GCCAAGAAUGCUCCCAUAAU	van Dijk *et al* (2007)
EXOSC9 #2	GCAGAAAUUACAGAGCUAA[dT][dT]	Sigma
TTC37 #1	CAGUGAGACUCGACAGUAA	Dharmacon
TTC37 #2	GUGUUAUGGUCGUGCAUUA	Dharmacon
WDR61 #1	AGGAACUCAUGUCGGGAAA	Dharmacon
WDR61 #2	CAAAGAGAAUGUACGGAUU	Dharmacon
SKIV2L2 #1	GCACAUACCUCAGCGGGAA	Dharmacon
SKIV2L2 #2	AGGGAAAAGCAGCGUGUAA	Dharmacon
XRN2 #1	AAGAGUACAGAUGAUCAUGUU	West *et al* (2004)
XRN2 #2	ACACUGUAGUCAGUAUUAAUU	Wagschal *et al* (2012)
TNKS1BP1 #1	GAUUCACUGGGUACCUACA[dT][dT]	Sigma
TNKS1BP1 #2	CACUUUGUGCCUCCUGGGA[dT][dT]	Sigma
ANXA1	GUGUUCAAUACCAUCCUUA[dT][dT]	Sigma
LRRC15	CUGACUACCAUUCAGGUCA[dT][dT]	Sigma
SPARC	CUACAUCGGGCCUUGCAAA[dT][dT]	Sigma
**Chemicals, enzymes and other reagents**		
Human IL-4	R&D Systems	6507-IL-010/CF
Human IL-12	R&D Systems	10018-IL-010
Human IFN-beta	R&D Systems	8499-IF-010/CF
actinomycin D	Fisher	AAJ60148LB0
qScript XLT	Quanta	95132-500
SsoAdvanced SYBR green supermix	Bio-Rad	1725274
**Software**		
SAMtools	Li *et al* (2009)	
BEDtools	Quinlan (2014)	
BBMAP	https://sourceforge.net/projects/bbmap/	
TrimGalore	https://www.bioinformatics.babraham.ac.uk/projects/trim_galore/	
Guppy v4.2.2		
MiniMap2	Li (2018)	
Nanopolish v0.13.3	https://github.com/jts/nanopolish	
**Other**		
NextSeq 550	Illumina	
CellInsight CX7 LZR	Thermofisher	
Dynabeads™ mRNA Purification Kit	Fisher	61006
NEBNext® Ultra™ II RNA Library Prep Kit for Illumina	NEB	E7300S
MinION MkIb	Oxford Nanopore Technologies Ltd	

© 2023 The Authors

Methods and Protocols

Cell infection and virus production

Normal human dermal fibroblasts (NHDFs) were obtained from Lonza, grown in DMEM supplemented with 5% FBS in the presence of penicillin and streptomycin, and routinely screened for mycoplasma contamination. HCMV AD169-GFP was a gift of Dong Yu (Terhune *et al*, 2007), and propagation and titration have been described previously (McKinney *et al*, 2014). TB40/E-GFP was a kind gift of Meaghan Hancock (Umashankar *et al*, 2011) and was similarly propagated. Briefly, virus stocks were concentrated by centrifugation at 20,000 rpm in an SW28 rotor for 75 min at 18°C and resuspended in 1.5% bovine serum albumin in DMEM. Titers of virus stocks were determined using a plaque assay. In experiments, released virus titers were determined by TCID50 assay, as previously described (McKinney *et al*, 2014). Vaccinia virus (Western Reserve strain) and HSV-1 (F strain) were propagated and titrated as previously described (Burgess & Mohr, 2015, 2018).

Biosafety

HCMV experiments were conducted under biosafety level 2 conditions at NYU School of Medicine and University of Surrey according to the specific operating procedures approved by each institution.

Transfection and cell treatments

For gene knockdowns, cells were seeded to multiwell plates and transfected the next day using 3 μl/ml Lipofectamine RNAiMax (Life Technologies) at a final siRNA concentration of 20 nM and infected 3 days post-transfection. siRNAs used are detailed in the reagent table. Knockdown efficiency was established by assaying target mRNA depletion by RT-qPCR. Caf1 inhibitor (8j) (Jadhav *et al*, 2015) was reconstituted in DMSO as a 10 mM stock solution, stored at −20°C, and diluted in appropriate infection media prior to cell treatment and following virus inoculum removal, i.e., 1.5 HPI. Human IL-4, IL-12, and IFN-beta (R&D Systems) were reconstituted in PBS and diluted in growth media at the indicated concentrations prior to cell treatment. For mRNA decay assays cells were treated with actinomycin D at 10 μg/ml for the indicated times prior to RNA isolation and RT-PCR analysis as described below.

Immunoblotting

Antibodies used for immunoblotting are listed in the reagent table. Immunoblots were visualized using an iBright CL1000 system (Life Technologies).

RT-qPCR analysis

Total RNA was isolated from infected cells using TRIzol. For each sample, 500 ng of RNA was subject to cDNA synthesis using qScript XLT (Quanta). Quantitative PCR (qPCR) reactions were conducted using Bio-Rad SsoAdvanced SYBR green supermix and a Bio-Rad CFX96 real-time system. Primer sequences are detailed in the reagent table. For each biological replicate, technical duplicates were conducted. mRNA levels relative to GAPDH were calculated using the $\Delta\Delta C_T$ method (Livak & Schmittgen, 2001), and statistical analyses were performed using GraphPad Prism.

CellInsight CX7 LZR high-content screening platform

To monitor virus spread and cell titer in drug treatment and gene knockdown conditions NHDFs were seeded in black-walled clear-bottom 96-well plates and knockdowns and infections conducted as above. Cells were fixed in 4% paraformaldehyde for 30 min and permeabilized in 0.5% triton X-100 in PBS for 15 min before blocking in immunofluorescence blocking buffer (4% FBS in PBS) for 1 h. Infected cells were detected by incubation with anti-IE1/2 antibody (1:250) overnight at 4°C followed by incubation with anti-mouse AlexaFluor 647 secondary (Invitrogen, A32787) and DAPI for 2 h at room temperature. Plates were imaged using a CellInsight CX7 LZR high-content screening platform by collecting 9 images at 4× magnification to cover the entire well. HCS Navigator software was used to quantify cell number by DAPI staining and the percentage infected cells, indicated by AlexaFluor 647 positivity.

Illumina RNA sequencing and analysis

RNA-Seq libraries were prepared from poly(A)-selected RNA using the NEBNext® Ultra™ II RNA Library Prep Kit for Illumina (New England Biolabs) and sequenced using a NextSeq 550. Sequence reads (Dataset EV3) were trimmed using TrimGalore (https://www.bioinformatics.babraham.ac.uk/projects/trim_galore/; --paired --length 30 –quality 30) and aligned against a hybrid genome comprising HG38 and the HCMV TB40/BAC4 genome (EF999921.1) using BBMAP (ambiguous = random sam = 1.3 minid = 0.9 maxindel = 200 k intronlen = 20; https://sourceforge.net/projects/bbmap/). Post-alignment processing was performed using SAMtools (Li *et al*, 2009) and BEDtools (Quinlan, 2014) to generate Bedgraph files.

Nanopore direct RNA sequencing

For each biological sample, between 750 and 850 ng of poly(A) RNA was isolated from up to 30 μg of total RNA using the Dynabeads™ mRNA Purification Kit (Invitrogen, 61006). Isolated poly(A) RNA was subsequently spiked with 0.5 μl of a synthetic Enolase 2 (ENO2) calibration RNA (Oxford Nanopore Technologies Ltd.), and dRNA-Seq libraries prepared according to the SQK-RNA002 protocol developed by Oxford Nanopore Technologies Ltd. (ONT). Sequencing was performed on a MinION MkIb using R9.4.1 (rev D) flow cells (ONT) for 24–48 h (one library per flowcell) and yielded up to 1,500,000 reads per dataset (Dataset EV4). Raw fast5 datasets were then basecalled using Guppy v4.2.2 (-c rna_r9.4.1_70bps_hac.cfg --calib_detect --trim_strategy rna --reverse_sequence true) with only reads passing filter used for subsequent analyses. For the analysis of viral RNAs, sequence reads were aligned against the HCMV TB40/BAC4 genome (EF999921.1) using MiniMap2 (Li, 2018; -ax splice -k14 -uf --secondary = no), with subsequent parsing through SAMtools (Li *et al*, 2009) and BEDtools (Quinlan, 2014). For the analysis of human RNAs, MiniMap2 (-ax map-ont -L -p 0.99 -uf --secondary = no) was used to align the sequence reads against a Homo Sapiens transcriptome database comprising protein coding and long noncoding transcripts derived from Gencode 37. Subsequent filtering of alignments (SAMtools, BEDtools) retained only unique alignments with a mapping (Q) score ≥ 10. Poly(A) tail lengths were estimated for all dRNA-Seq reads using Nanopolish v0.13.3 (https://github.com/jts/nanopolish) with default parameters. For the paired plots (Fig 6G), only human and viral RNAs supported by at least 50 DRS reads were used.

© 2023 The Authors

Data visualization

Bedgraph and Nanopolish output were processed using R and ggplot2 (Wickham, 2009). Schematics were created using BioRender.com

Data availability

All sequencing datasets associated with this study are available as raw fastq files (Illumina) and raw fast5 files (Nanopore) via the European Nucleotide Archive under the accession PRJEB45677. Bedgraph and nanopolish poly(A) datasets used in our analyses are available at https://github.com/DepledgeLab/CCR4-NOT-regulates-HCMV.

Expanded View for this article is available online.

Acknowledgements

We thank Dr Angus Wilson and Dr David Levy for helpful discussions. This work was supported by NIH grants AI166638 and AI151436 (to HMB), GM056927, AI073898 (to IM), and AI152543 (to IM and DPD). RG was supported in part by T32 AI100853 and T32 AI007180. DPD is funded by the German Centre for Infection Research (DZIF TTU 07.708).

Author contributions

Hannah M Burgess: Conceptualization; data curation; formal analysis; supervision; funding acquisition; validation; investigation; visualization; methodology; writing – original draft; project administration; writing – review and editing. **Rebecca Grande:** Investigation. **Sofia Riccio:** Investigation. **Ikshitaa Dinesh:** Investigation. **Gerlof Sebastiaan Winkler:** Resources; writing – review and editing. **Daniel P Depledge:** Data curation; formal analysis; investigation; writing – review and editing. **Ian Mohr:** Resources; supervision; funding acquisition; writing – review and editing.

Disclosure and competing interests statement

The authors declare that they have no conflict of interest.

References

Baer BW, Kornberg RD (1983) The protein responsible for the repeating structure of cytoplasmic poly(A)-ribonucleoprotein. *J Cell Biol* 96: 717 – 721

Batra R, Stark TJ, Clark E, Belzile JP, Wheeler EC, Yee BA, Huang H, Gelboin-Burkhart C, Huelga SC, Aigner S *et al* (2016) RNA-binding protein CPEB1 remodels host and viral RNA landscapes. *Nat Struct Mol Biol* 23: 1101 – 1110

Bianco C, Mohr I (2019) Ribosome biogenesis restricts innate immune responses to virus infection and DNA. *Elife* 8: e49551

Boeckh M, Geballe AP (2011) Cytomegalovirus: pathogen, paradigm, and puzzle. *J Clin Invest* 121: 1673 – 1680

Boland A, Chen Y, Raisch T, Jonas S, Kuzuoğlu-Öztürk D, Wohlbold L, Weichenrieder O, Izaurralde E (2013) Structure and assembly of the NOT module of the human CCR4-NOT complex. *Nat Struct Mol Biol* 20: 1289 – 1297

Burgess HM, Mohr I (2015) Cellular 5′-3’ mRNA exonuclease Xrn1 controls double-stranded RNA accumulation and anti-viral responses. *Cell Host Microbe* 17: 332 – 344

Burgess HM, Mohr I (2018) Defining the role of stress granules in innate immune suppression by the herpes simplex virus 1 endoribonuclease VHS. *J Virol* 92: e00829-18

Burgess HM, Vink EI, Mohr I (2022) Minding the message: tactics controlling RNA decay, modification, and translation in virus-infected cells. *Genes Dev* 36: 108 – 132

Buschauer R, Matsuo Y, Sugiyama T, Chen YH, Alhusaini N, Sweet T, Ikeuchi K, Cheng J, Matsuki Y, Nobuta R *et al* (2020) The Ccr4-Not complex monitors the translating ribosome for codon optimality. *Science* 368: eaay6912

Chalabi Hagkarim N, Ryan EL, Byrd PJ, Hollingworth R, Shimwell NJ, Agathanggelou A, Vavasseur M, Kolbe V, Speiseder T, Dobner T *et al* (2018) Degradation of a novel DNA damage response protein, tankyrase 1 binding protein 1, following adenovirus infection. *J Virol* 92: e02034-17

Chang H, Lim J, Ha M, Kim VN (2014) TAIL-seq: genome-wide determination of poly(A) tail length and 3′ end modifications. *Mol Cell* 53: 1044 – 1052

Dauber B, Pelletier J, Smiley JR (2011) The herpes simplex virus 1 vhs protein enhances translation of viral true late mRNAs and virus production in a cell type-dependent manner. *J Virol* 85: 5363 – 5373

Dauber B, Saffran HA, Smiley JR (2019) The herpes simplex virus host shutoff (vhs) RNase limits accumulation of double stranded RNA in infected cells: evidence for accelerated decay of duplex RNA. *PLoS Pathog* 15: e1008111

van Dijk EL, Schilders G, Pruijn GJM (2007) Human cell growth requires a functional cytoplasmic exosome, which is involved in various mRNA decay pathways. *RNA* 13: 1027 – 1035

Eckmann CR, Rammelt C, Wahle E (2011) Control of poly(A) tail length. *Wiley Interdiscip Rev RNA* 2: 348 – 361

Geist LJ, Dai LY (1996) Cytomegalovirus modulates interleukin-6 gene expression. *Transplantation* 62: 653 – 658

Gopal S, Perez E Jr, Xia AY, Knowlton JJ, Cerqueira F, Dermody TS, Upton JW (2018) Murine cytomegalovirus M72 promotes acute virus replication in vivo and is a substrate of the TRiC/CCT complex. *Virology* 522: 92 – 105

Griffiths P, Baraniak I, Reeves M (2015) The pathogenesis of human cytomegalovirus. *J Pathol* 235: 288 – 297

Harwardt T, Lukas S, Zenger M, Reitberger T, Danzer D, Übner T, Munday DC, Nevels M, Paulus C (2016) Human cytomegalovirus immediate-early 1 protein rewires upstream STAT3 to downstream STAT1 signaling switching an IL6-type to an IFNγ-like response. *PLoS Pathog* 12: e1005748

Hein MY, Weissman JS (2022) Functional single-cell genomics of human cytomegalovirus infection. *Nat Biotechnol* 40: 391 – 401

Hennig T, Djakovic L, Dölken L, Whisnant AW (2021) A review of the multipronged attack of herpes simplex virus 1 on the host transcriptional machinery. *Viruses* 13: 1836

Hsu KL, Yen HS, Yeang CH (2022) Cooperative stability renders protein complex formation more robust and controllable. *Sci Rep* 12: 10490

Jadhav GP, Kaur I, Maryati M, Airhihen B, Fischer PM, Winkler GS (2015) Discovery, synthesis and biochemical profiling of purine-2,6-dione derivatives as inhibitors of the human poly(A)-selective ribonuclease Caf1. *Bioorg Med Chem Lett* 25: 4219 – 4224

Jassal B, Matthews L, Viteri G, Gong C, Lorente P, Fabregat A, Sidiropoulos K, Cook J, Gillespie M, Haw R *et al* (2020) The reactome pathway knowledgebase. *Nucleic Acids Res* 48: D498 – D503

Keegan AD, Leonard WJ, Zhu J (2021) Recent advances in understanding the role of IL-4 signaling. *Fac Rev* 10: 71

© 2023 The Authors

Kim D, Lee YS, Jung SJ, Yeo J, Seo JJ, Lee YY, Lim J, Chang H, Song J, Yang J *et al* (2020) Viral hijacking of the TENT4-ZCCHC14 complex protects viral RNAs via mixed tailing. *Nat Struct Mol Biol* 27: 581 – 588

Kühn U, Wahle E (2004) Structure and function of poly(A) binding proteins. *Biochim Biophys Acta* 1678: 67 – 84

Kwong AD, Frenkel N (1987) Herpes simplex virus-infected cells contain a function(s) that destabilizes both host and viral mRNAs. *Proc Natl Acad Sci USA* 84: 1926 – 1930

Li H (2018) Minimap2: pairwise alignment for nucleotide sequences. *Bioinformatics* 34: 3094 – 3100

Li H, Handsaker B, Wysoker A, Fennell T, Ruan J, Homer N, Marth G, Abecasis G, Durbin R (2009) The Sequence Alignment/Map format and SAMtools. *Bioinformatics* 25: 2078 – 2079

Lim J, Kim D, Lee YS, Ha M, Lee M, Yeo J, Chang H, Song J, Ahn K, Kim VN (2018) Mixed tailing by TENT4A and TENT4B shields mRNA from rapid deadenylation. *Science* 361: 701 – 704

Livak KJ, Schmittgen TD (2001) Analysis of relative gene expression data using real-time quantitative PCR and the 2(-Delta Delta C(T)) Method. *Methods* 25: 402 – 408

Mattijssen S, Iben JR, Li T, Coon SL, Maraia RJ (2020) Single molecule poly(A) tail-seq shows LARP4 opposes deadenylation throughout mRNA lifespan with most impact on short tails. *Elife* 9: e59186

McKinney C, Perez C, Mohr I (2012) Poly(A) binding protein abundance regulates eukaryotic translation initiation factor 4F assembly in human cytomegalovirus-infected cells. *Proc Natl Acad Sci USA* 109: 5627 – 5632

McKinney C, Zavadil J, Bianco C, Shiflett L, Brown S, Mohr I (2014) Global reprogramming of the cellular translational landscape facilitates cytomegalovirus replication. *Cell Rep* 6: 9 – 17

Morita M, Oike Y, Nagashima T, Kadomatsu T, Tabata M, Suzuki T, Nakamura T, Yoshida N, Okada M, Yamamoto T (2011) Obesity resistance and increased hepatic expression of catabolism-related mRNAs in Cnot3+/− mice. *EMBO J* 30: 4678 – 4691

Nobre LV, Nightingale K, Ravenhill BJ, Antrobus R, Soday L, Nichols J, Davies JA, Seirafian S, Wang EC, Davison AJ *et al* (2019) Human cytomegalovirus interactome analysis identifies degradation hubs, domain associations and viral protein functions. *Elife* 8: e49894

Pasieka TJ, Lu B, Crosby SD, Wylie KM, Morrison LA, Alexander DE, Menachery VD, Leib DA (2008) Herpes simplex virus virion host shutoff attenuates establishment of the antiviral state. *J Virol* 82: 5527 – 5535

Passmore LA, Coller J (2022) Roles of mRNA poly(A) tails in regulation of eukaryotic gene expression. *Nat Rev Mol Cell Biol* 23: 93 – 106

Perera MR, Wills MR, Sinclair JH (2021) HCMV antivirals and strategies to target the latent reservoir. *Viruses* 13: 817

Perez C, McKinney C, Chulunbaatar U, Mohr I (2011) Translational control of the abundance of cytoplasmic poly(A) binding protein in human cytomegalovirus-infected cells. *J Virol* 85: 156 – 164

Quinlan AR (2014) BEDTools: the Swiss-Army Tool for genome feature analysis. *Curr Protoc Bioinformatics* 47: 11.12.11 – 11.12.34

Raisch T, Chang CT, Levdansky Y, Muthukumar S, Raunser S, Valkov E (2019) Reconstitution of recombinant human CCR4-NOT reveals molecular insights into regulated deadenylation. *Nat Commun* 10: 3173

Reitsma JM, Sato H, Nevels M, Terhune SS, Paulus C (2013) Human cytomegalovirus IE1 protein disrupts interleukin-6 signaling by sequestering STAT3 in the nucleus. *J Virol* 87: 10763 – 10776

Rubio RM, Depledge DP, Bianco C, Thompson L, Mohr I (2018) RNA m(6) A modification enzymes shape innate responses to DNA by regulating interferon beta. *Genes Dev* 32: 1472 – 1484

Rutkowski AJ, Erhard F, L'Hernault A, Bonfert T, Schilhabel M, Crump C, Rosenstiel P, Efstathiou S, Zimmer R, Friedel CC *et al* (2015) Widespread disruption of host transcription termination in HSV-1 infection. *Nat Commun* 6: 7126

Scarpini S, Morigi F, Betti L, Dondi A, Biagi C, Lanari M (2021) Development of a vaccine against human cytomegalovirus: advances, barriers, and implications for the clinical practice. *Vaccines (Basel)* 9: 551

Shin J, Paek KY, Chikhaoui L, Jung S, Ponny S, Suzuki Y, Padmanabhan K, Richter JD (2022) Oppositional poly(A) tail length regulation by FMRP and CPEB1. *RNA* 28: 756 – 765

Song J, Lee S, Cho DY, Lee S, Kim H, Yu N, Lee S, Ahn K (2019) Human cytomegalovirus induces and exploits Roquin to counteract the IRF1-mediated antiviral state. *Proc Natl Acad Sci USA* 116: 18619 – 18628

Stoney PN, Yanagiya A, Nishijima S, Yamamoto T (2022) CNOT7 outcompetes its paralog CNOT8 for integration into the CCR4-NOT complex. *J Mol Biol* 434: 167523

Tai-Schmiedel J, Karniely S, Lau B, Ezra A, Eliyahu E, Nachshon A, Kerr K, Suárez N, Schwartz M, Davison AJ *et al* (2020) Human cytomegalovirus long noncoding RNA4.9 regulates viral DNA replication. *PLoS Pathog* 16: e1008390

Terhune S, Torigoi E, Moorman N, Silva M, Qian Z, Shenk T, Yu D (2007) Human cytomegalovirus UL38 protein blocks apoptosis. *J Virol* 81: 3109 – 3123

Thompson L, Depledge DP, Burgess HM, Mohr I (2022) An eIF3d-dependent switch regulates HCMV replication by remodeling the infected cell translation landscape to mimic chronic ER stress. *Cell Rep* 39: 110767

Tirosh O, Cohen Y, Shitrit A, Shani O, Le-Trilling VTK, Trilling M, Friedlander G, Tanenbaum M, Stern-Ginossar N (2015) The transcription and translation landscapes during human cytomegalovirus infection reveal novel host-pathogen interactions. *PLoS Pathog* 11: e1005288

Umashankar M, Petrucelli A, Cicchini L, Caposio P, Kreklywich CN, Rak M, Bughio F, Goldman DC, Hamlin KL, Nelson JA *et al* (2011) A novel human cytomegalovirus locus modulates cell type-specific outcomes of infection. *PLoS Pathog* 7: e1002444

Vignali DA, Kuchroo VK (2012) IL-12 family cytokines: immunological playmakers. *Nat Immunol* 13: 722 – 728

Wagschal A, Rousset E, Basavarajaiah P, Contreras X, Harwig A, Laurent-Chabalier S, Nakamura M, Chen X, Zhang K, Meziane O *et al* (2012) Microprocessor, Setx, Xrn2, and Rrp6 co-operate to induce premature termination of transcription by RNAPII. *Cell* 150: 1147 – 1157

Walsh D, Perez C, Notary J, Mohr I (2005) Regulation of the translation initiation factor eIF4F by multiple mechanisms in human cytomegalovirus-infected cells. *J Virol* 79: 8057 – 8064

Weekes MP, Tomasec P, Huttlin EL, Fielding CA, Nusinow D, Stanton RJ, Wang ECY, Aicheler R, Murrell I, Wilkinson GWG *et al* (2014) Quantitative temporal viromics: an approach to investigate host-pathogen interaction. *Cell* 157: 1460 – 1472

West S, Gromak N, Proudfoot NJ (2004) Human 5′ → 3′ exonuclease Xrn2 promotes transcription termination at co-transcriptional cleavage sites. *Nature* 432: 522 – 525

Wickham H (2009) *ggplot2: elegant graphics for data analysis*. New York, NY: Springer

Wilkinson GW, Davison AJ, Tomasec P, Fielding CA, Aicheler R, Murrell I, Seirafian S, Wang EC, Weekes M, Lehner PJ *et al* (2015) Human cytomegalovirus: taking the strain. *Med Microbiol Immunol* 204: 273 – 284

Workman RE, Tang AD, Tang PS, Jain M, Tyson JR, Razaghi R, Zuzarte PC, Gilpatrick T, Payne A, Quick J *et al* (2019) Nanopore native RNA

© 2023 The Authors

sequencing of a human poly(A) transcriptome. *Nat Methods* 16: 1297 – 1305

Yang Z, Wara-aswapati N, Yoshida Y, Walker N, Galson DL, Listman J, Auron PE (2002) Dual regulatory role of human cytomegalovirus immediate-early protein in IL1B transcription is dependent upon Spi-1/PU.1. *Biochem Biophys Res Commun* 294: 854 – 863

Yi H, Park J, Ha M, Lim J, Chang H, Kim VN (2018) PABP cooperates with the CCR4-NOT complex to promote mRNA deadenylation and block precocious decay. *Mol Cell* 70: 1081 – 1088.e5

Zhang ZJ, Gao Q, Fang XD, Ding ZH, Gao DM, Xu WY, Cao Q, Qiao JH, Yang YZ, Han C *et al* (2020) CCR4, a RNA decay factor, is hijacked by a plant cytorhabdovirus phosphoprotein to facilitate virus replication. *eLife* 9: e53753

License: This is an open access article under the terms of the Creative Commons Attribution License, which permits use, distribution and reproduction in any medium, provided the original work is properly cited.

 © 2023 The Authors

Expanded View Figures

Figure EV1. siRNA mini-screen controls.

A Target depletion for each siRNA was assessed by qRT-PCR. NHDF cells were transfected with 20 nM of either the nonsilencing control or the targeting siRNA as indicated and RNA isolated for analysis at 72 h post-transfection. $n = 2$ biological replicates.

B Cell viability following transfection with each mini-screen siRNA was assessed by quantifying cell number by DAPI nuclear staining and high content imaging at 72 h post-transfection. Each experiment was conducted 3 times with internal duplicates, normalized to control siRNA-treated cells and plotted as the mean ± SEM.

C The impact of Xrn1 depletion on released infectious viral titer was determined by replicating experimental conditions in (Fig 1B) in 12-well plates and establishing TCID50 from culture supernatants on NHDF cells, plotted as the mean ± SEM ($n = 3$ biological replicates). Statistical significance established by ANOVA test with Dunnett multiple comparison correction compared with control siRNA (ns) $P > 0.033$.

D NHDFs were transfected with siRNAs (#1 or #2) targeting each CCR4-NOT nuclease at 20 nM each or control siRNA at 80 nM. Cells were subsequently infected with HCMV (AD169) at low MOI (0.05). Protein lysates were collected at 7 DPI at immunoblotted for viral proteins and a loading control (GAPDH) as indicated.

© 2023 The Authors

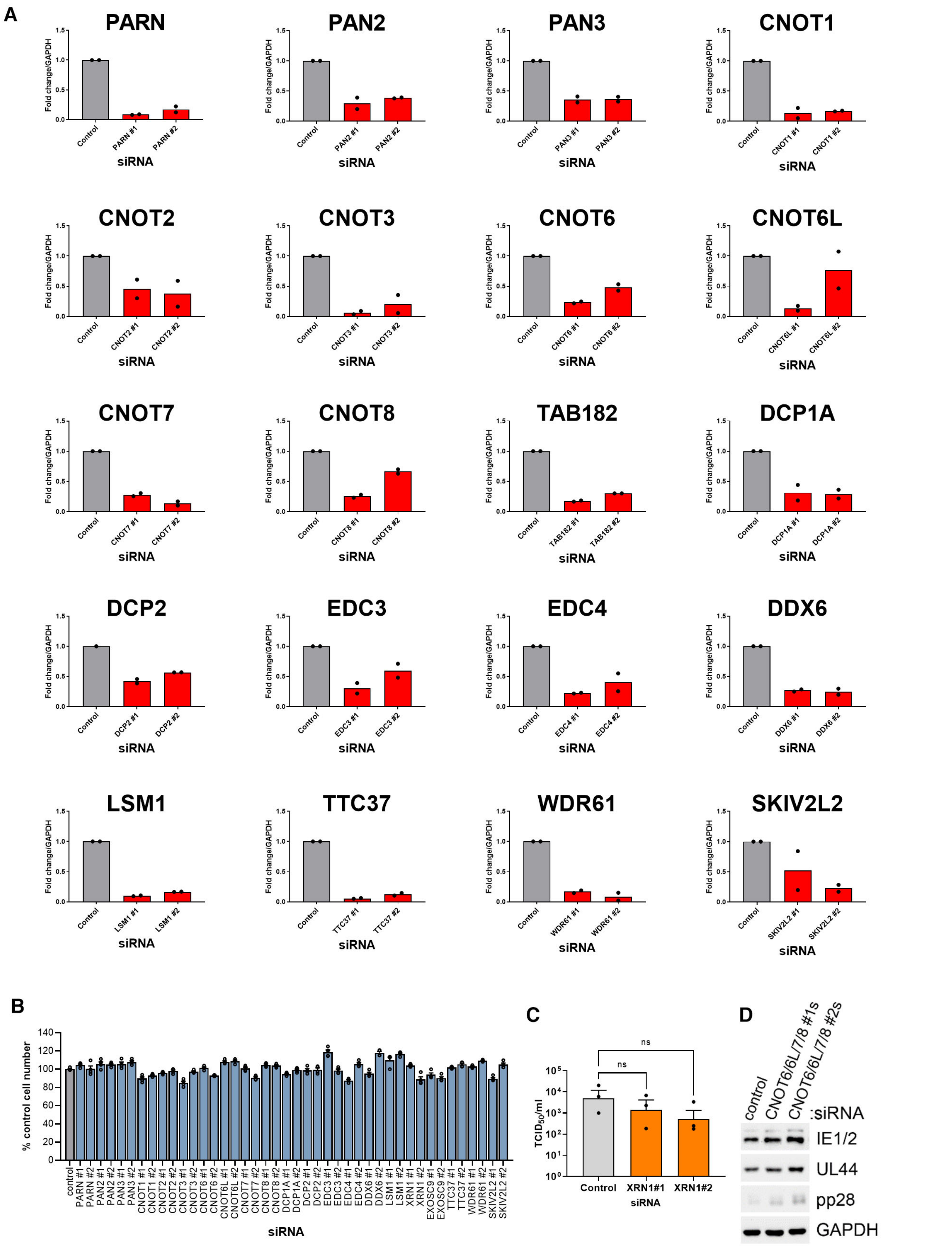

Figure EV1.

 © 2023 The Authors

Figure EV2. CNOT1 and CNOT3 promote viral gene expression late in HCMV infection.

A RT-qPCR analysis for CNOT1 and CNOT3 mRNAs from RNA isolated from uninfected cells treated as in Fig 2A. Mean fold changes relative to GAPDH ± SEM (n = 3 biological replicates) are plotted with statistical significance established by ANOVA test with Dunnett multiple comparison correction compared control siRNA treated samples; (*) $P < 0.033$, (**) $P < 0.002$, (***) $P < 0.001$, no asterisk: not significant.

B RT-qPCR analysis for viral mRNAs from RNA isolated from cells siRNA-transfected and infected with HCMV AD169 as in Fig 2C. Protein names, where different from gene names, indicated in brackets. Mean fold changes relative to GAPDH ± SEM (n = 3 biological replicates) are plotted, normalized to siControl 6 HPI samples.

C Cells stained positively for IE1/2 expression by immunofluorescence were scored at 6HPI following infection (MOI: 3) of control and CNOT1/3 siRNA-transfected NHDFs. Mean % IE1/2 positive cells are plotted ± SEM (n = 3 biological replicates). No significant (ns) differences between control and knockdown cells were identified by ANOVA test with Dunnett multiple comparison correction.

D Immunoblot analysis of lysates from NHDF cells transfected with control or CNOT3 siRNA (#1) and infected with HCMV AD169 at MOI = 3 and analyzed as in Fig 2C.

E Immunoblot analysis of lysates from NHDF cells transfected with control or CNOT1 siRNA (#1) and infected with HCMV TB40/E at MOI = 3 and analyzed as in Fig 2C.

F Titer of infectious released virus was determined by TCID50 on supernatants of cells transfected with the indicated siRNAs and infected with HCMV clinical strain TB40/E at MOI = 0.05 and incubated for 7 days. Mean TCID50/ml of two biological experiments shown.

© 2023 The Authors

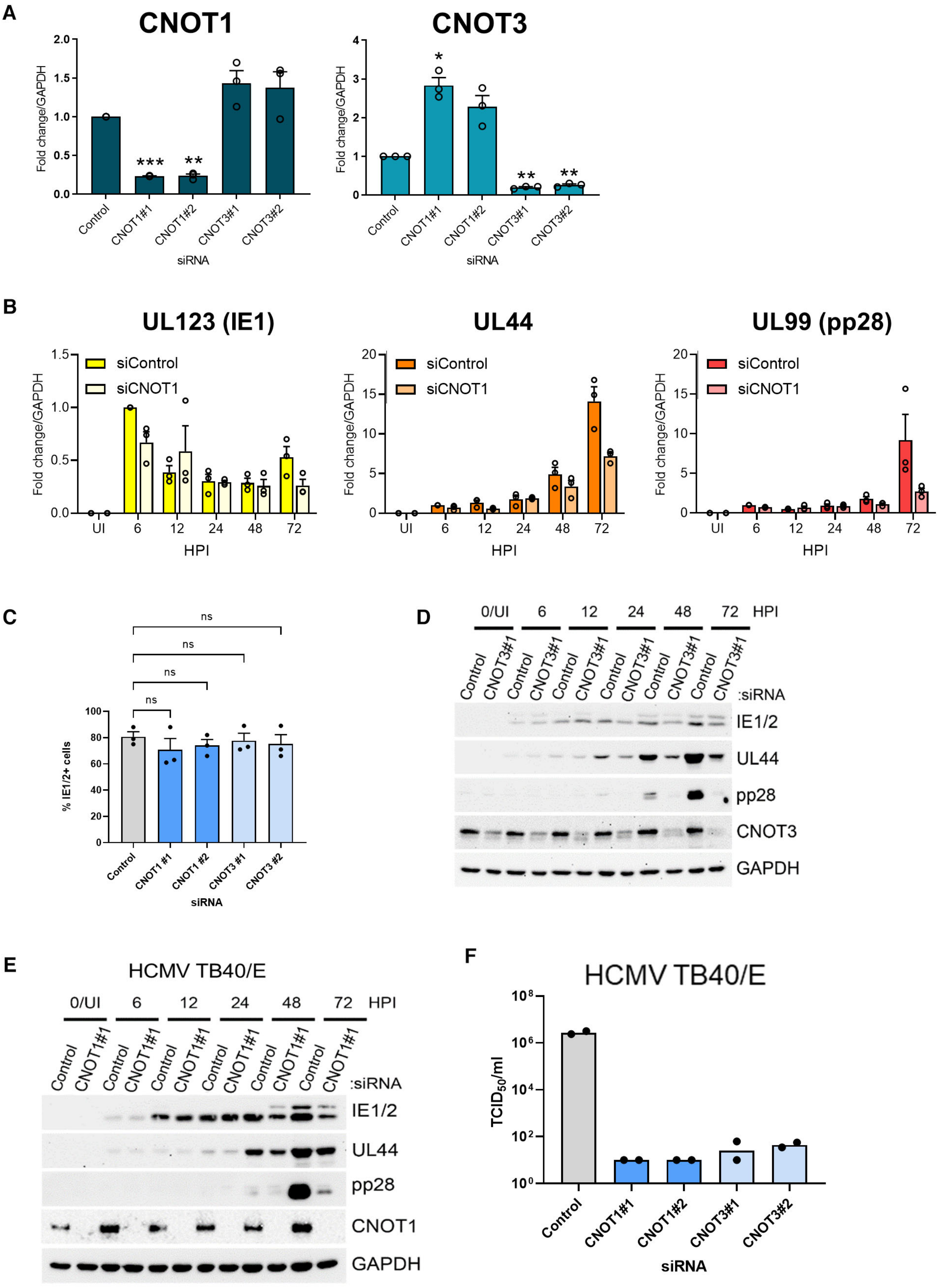

Figure EV2.

© 2023 The Authors

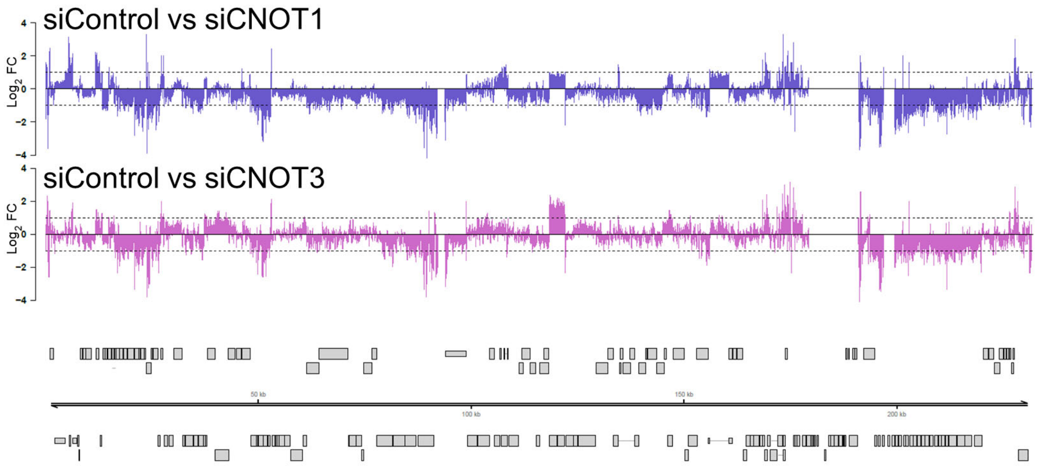

Figure EV3. Comparison of viral transcript genomic distribution in control vs. CNOT1/3 knockdown cells.

The distribution of 50,000, randomly sampled viral reads was compared between infected siControl and siCNOT1 cells (upper panel) and siControl and siCNOT3 cells (lower panel) and $\log_2$ fold change plotted across the viral genome. Canonical HCMV ORFs are shown as gray boxes.

Figure EV4. The impact and functional importance of host transcriptome changes upon CNOT1/3 knockdown.

A NHDFs were pretreated with indicated cytokines for 24 h and subsequently infected with HCMV (AD169) at low MOI (0.05). At 7 DPI-infected cells were identified as in Fig 1A using high content imaging. Mean % infected cells ± SEM, normalized to nontreated (NT) cells (n = 3 biological replicates) is plotted. Statistical significance established by ANOVA test with Dunnett multiple comparison correction compared control siRNA-treated samples; (ns) $P > 0.033$, (***) $P < 0.001$.

B Select differentially regulated transcripts were validated by RT-qPCR analysis from control or CNOT1 siRNA-transfected cells that were mock infected or infected with HCMV AD169 at MOI = 3 and collected at 72 HPI. Mean ± SEM (n = 3 biological replicates) fold change relative to GAPDH is plotted.

C siRNAs against host genes differentially expressed by CNOT1/3 knockdown were validated by RT-qPCR 3 days post-transfection of NHDFs.

Data information: Statistical significance in (B) and (C) was tested by students t-tests; (*) $P < 0.05$, (**) $P < 0.01$, (***) $P < 0.001$, no asterisk: not significant.

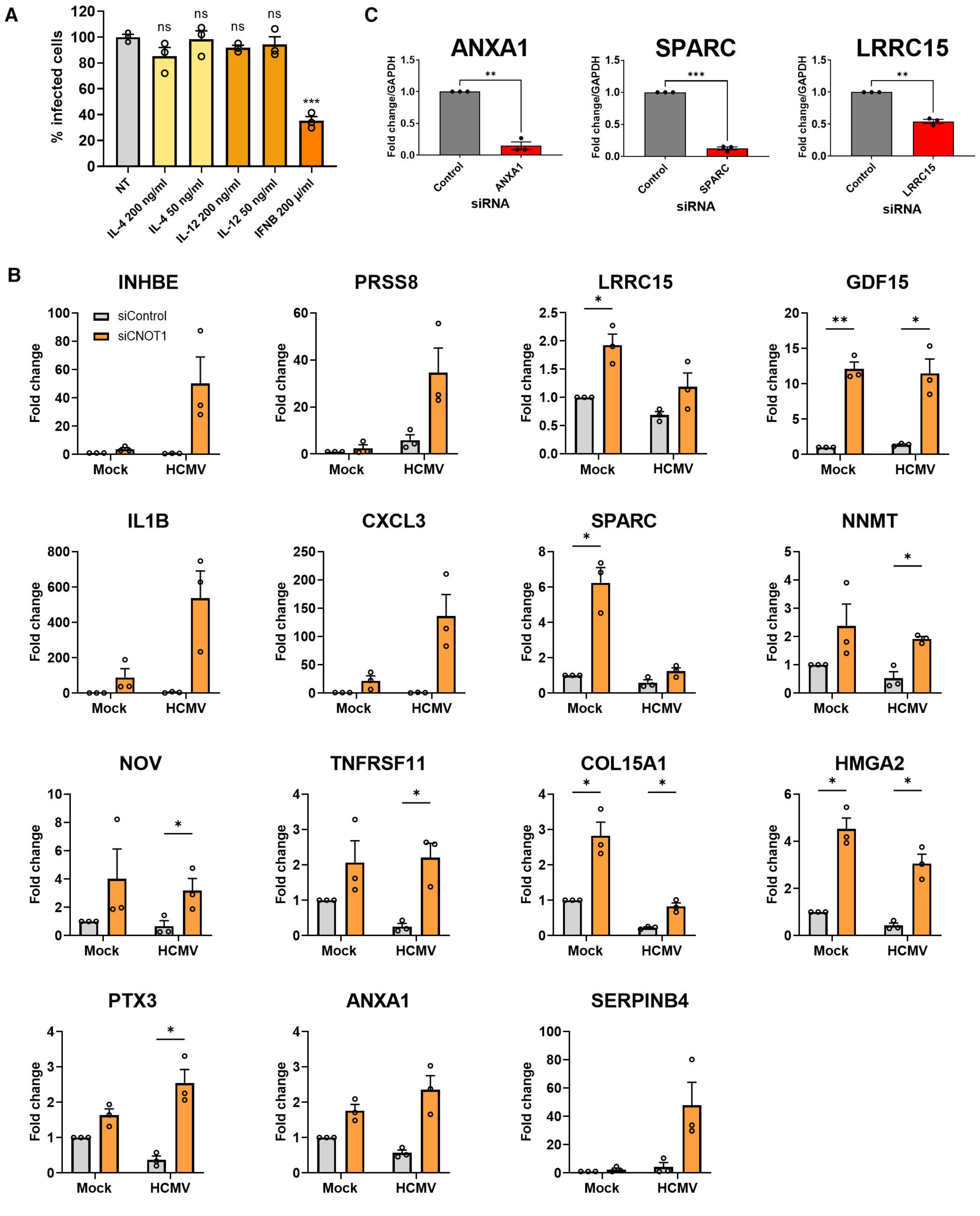

Figure EV4.

© 2023 The Authors

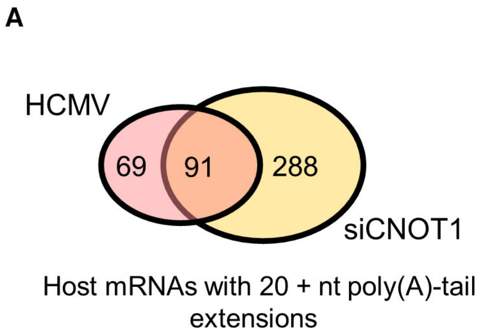

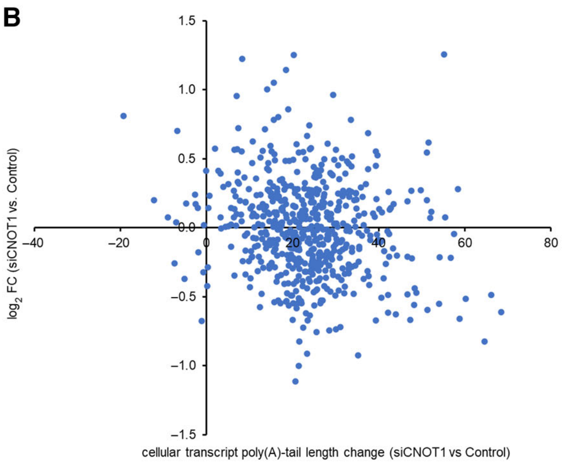

Figure EV5. Comparing poly(A)-tail length changes and RNA abundance changes of cellular transcripts in CNOT1/3-depleted and HCMV-infected cells.

A Venn diagram showing overlap of host genes detected with > 20 reads for which ≥ 20 nt poly(A) tail length additions were found at any time point post-infection (pink) compared to uninfected samples, and in siCNOT1 compared to siControl samples (yellow; average of two biological replicates).

B Expression changes of cellular genes upon CNOT1-depletion (Fig 5) are plotted against poly(A)-tail length changes upon CNOT1-depletion detected by DRS (mean of two biological replicates, Fig 6F).
