# Supplementary figures and images for: CCR4‐NOT differentially controls host versus virus poly(a)‐tail length and regulates HCMV infection
Source: EMBO Rep. 2023 Oct 17;24(12):e56327. doi: 10.15252/embr.202256327 (PMC10702830; doi:10.15252/embr.202256327)

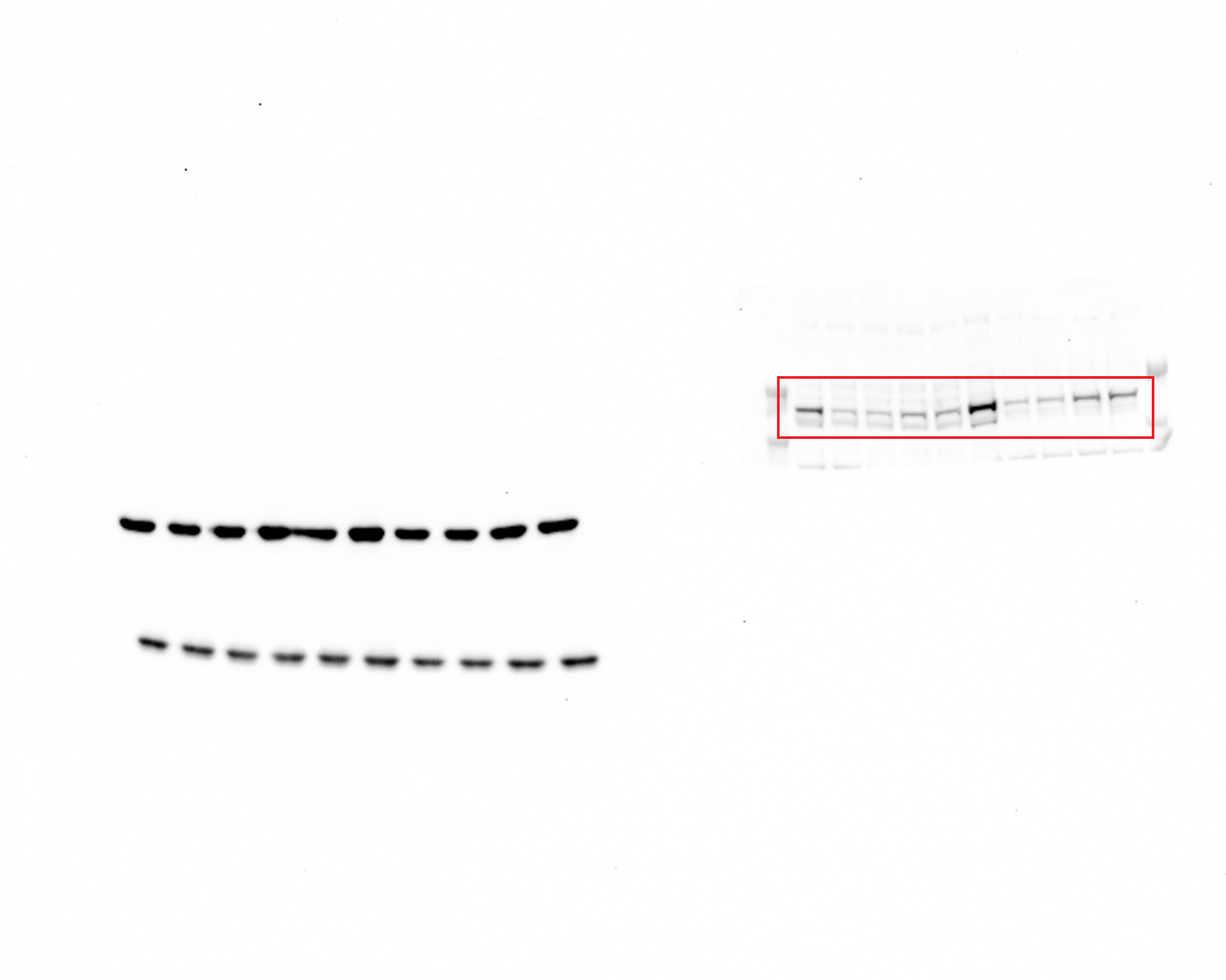

Supplement: Supplementary file 10 — Source Data for Figure 2 [file EMBR-24-e56327-s006.zip › Fig 2/Fig 2a/CNOT1.tif]

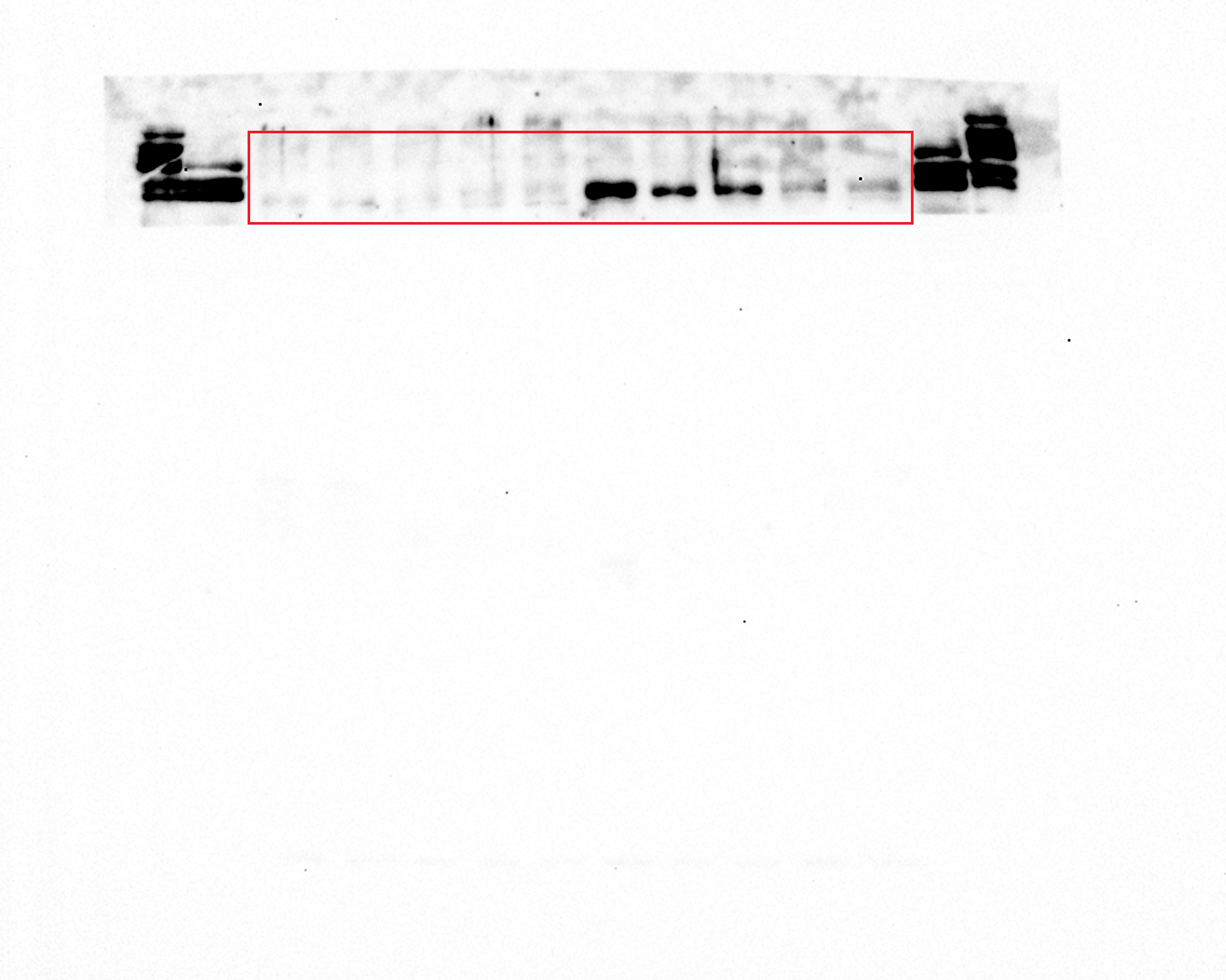

Supplement: Supplementary file 10 — Source Data for Figure 2 [file EMBR-24-e56327-s006.zip › Fig 2/Fig 2a/CNOT3.tif]

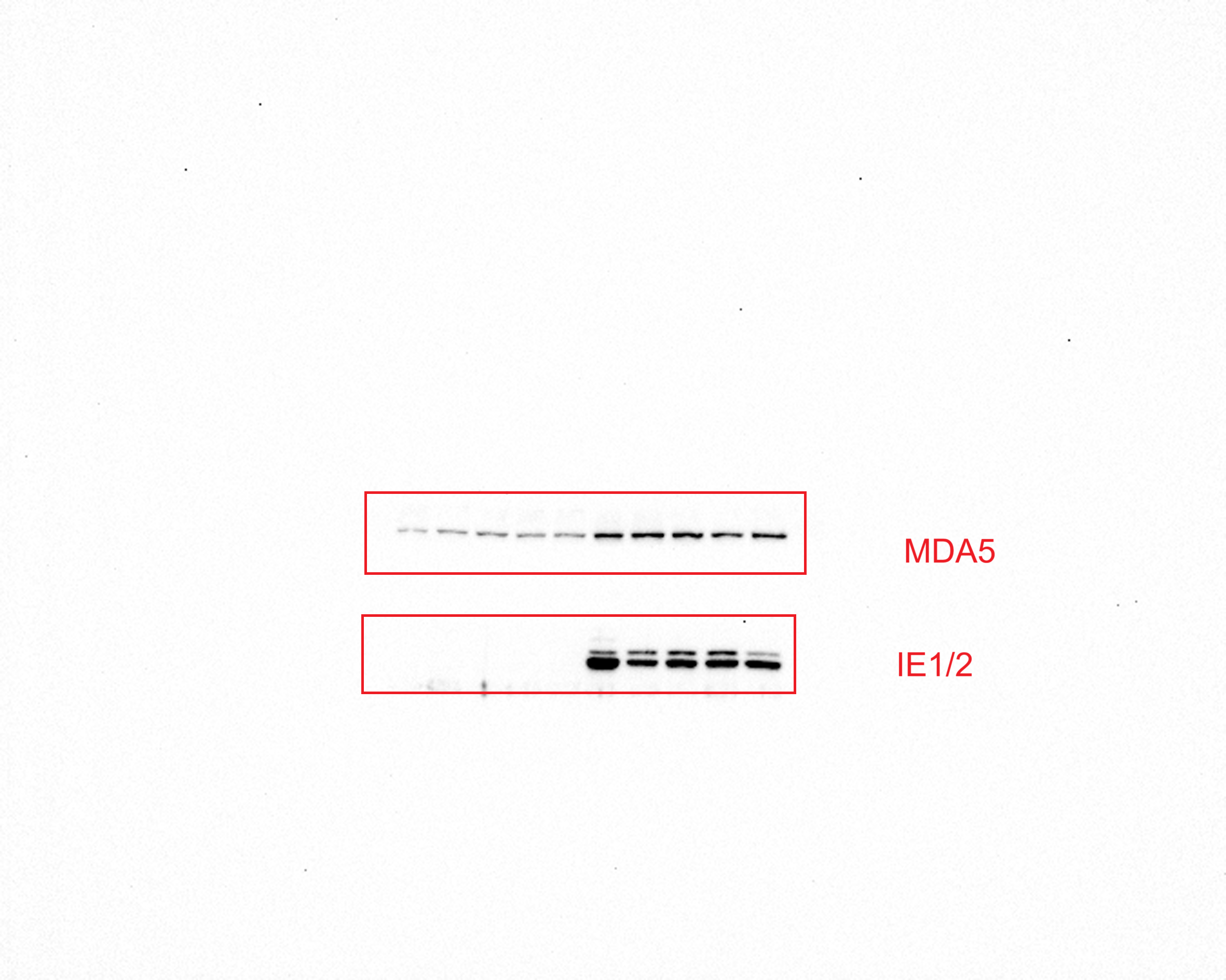

Supplement: Supplementary file 10 — Source Data for Figure 2 [file EMBR-24-e56327-s006.zip › Fig 2/Fig 2a/IE12 MDA5.tif]

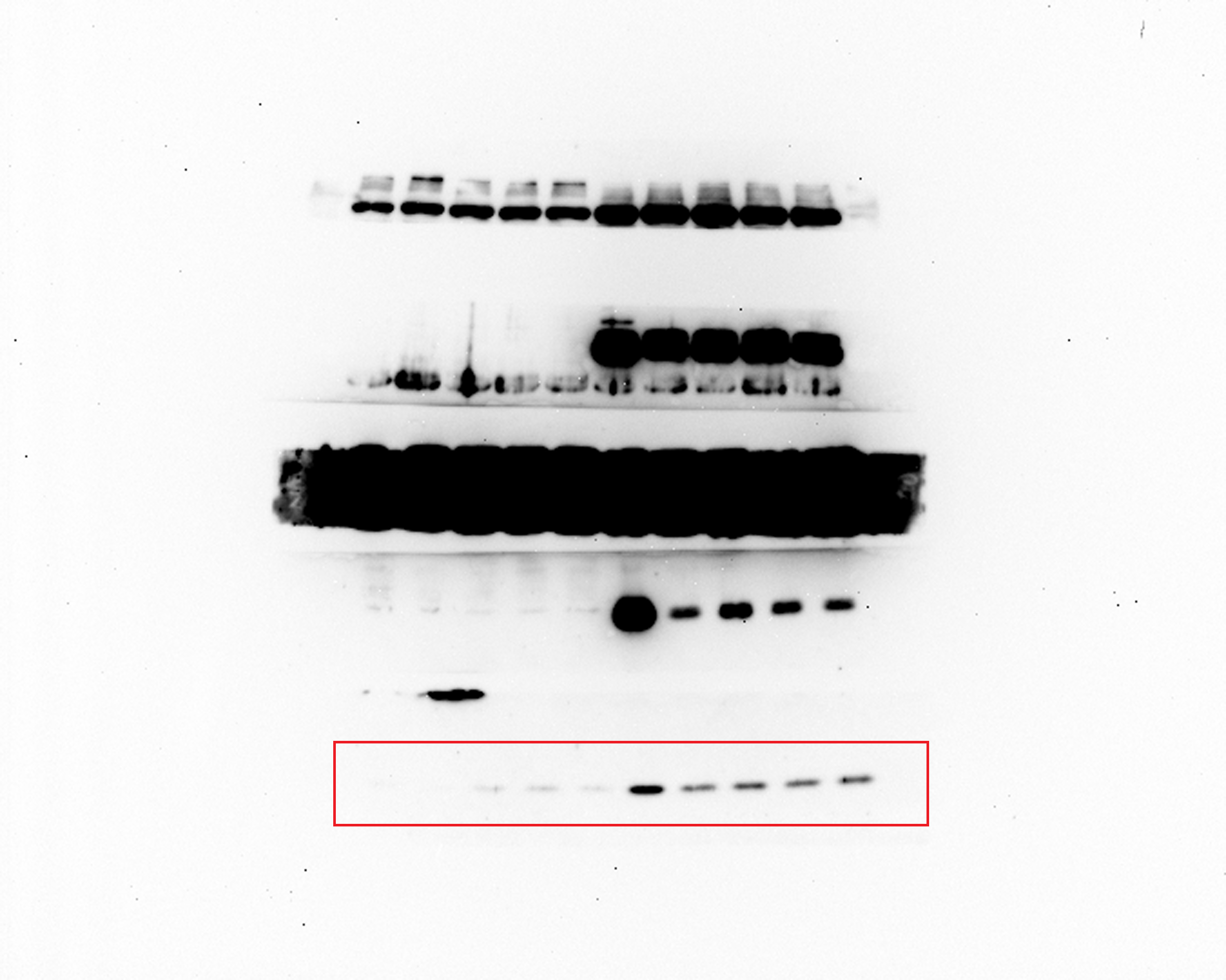

Supplement: Supplementary file 10 — Source Data for Figure 2 [file EMBR-24-e56327-s006.zip › Fig 2/Fig 2a/ISG15.tif]

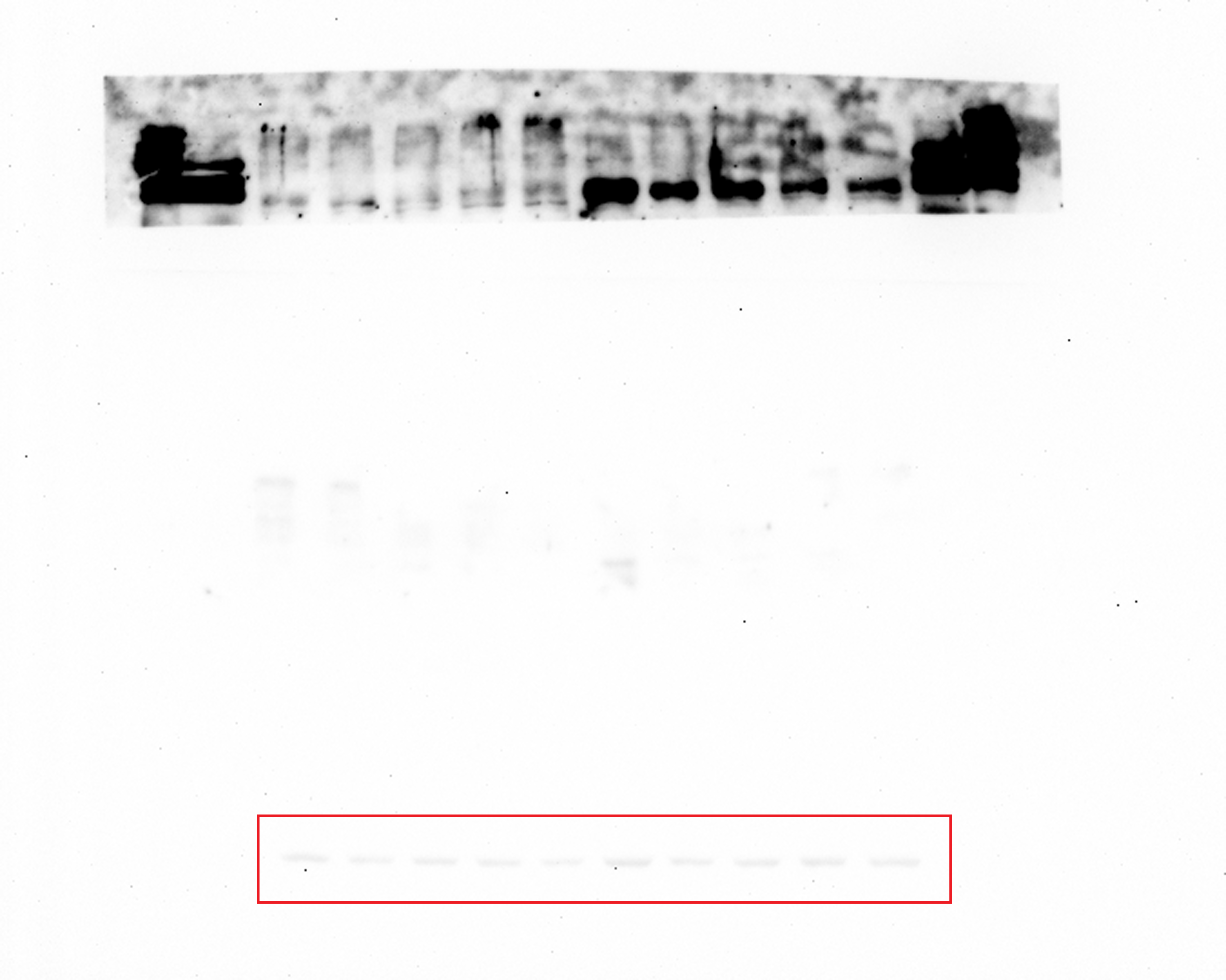

Supplement: Supplementary file 10 — Source Data for Figure 2 [file EMBR-24-e56327-s006.zip › Fig 2/Fig 2a/PKR.tif]

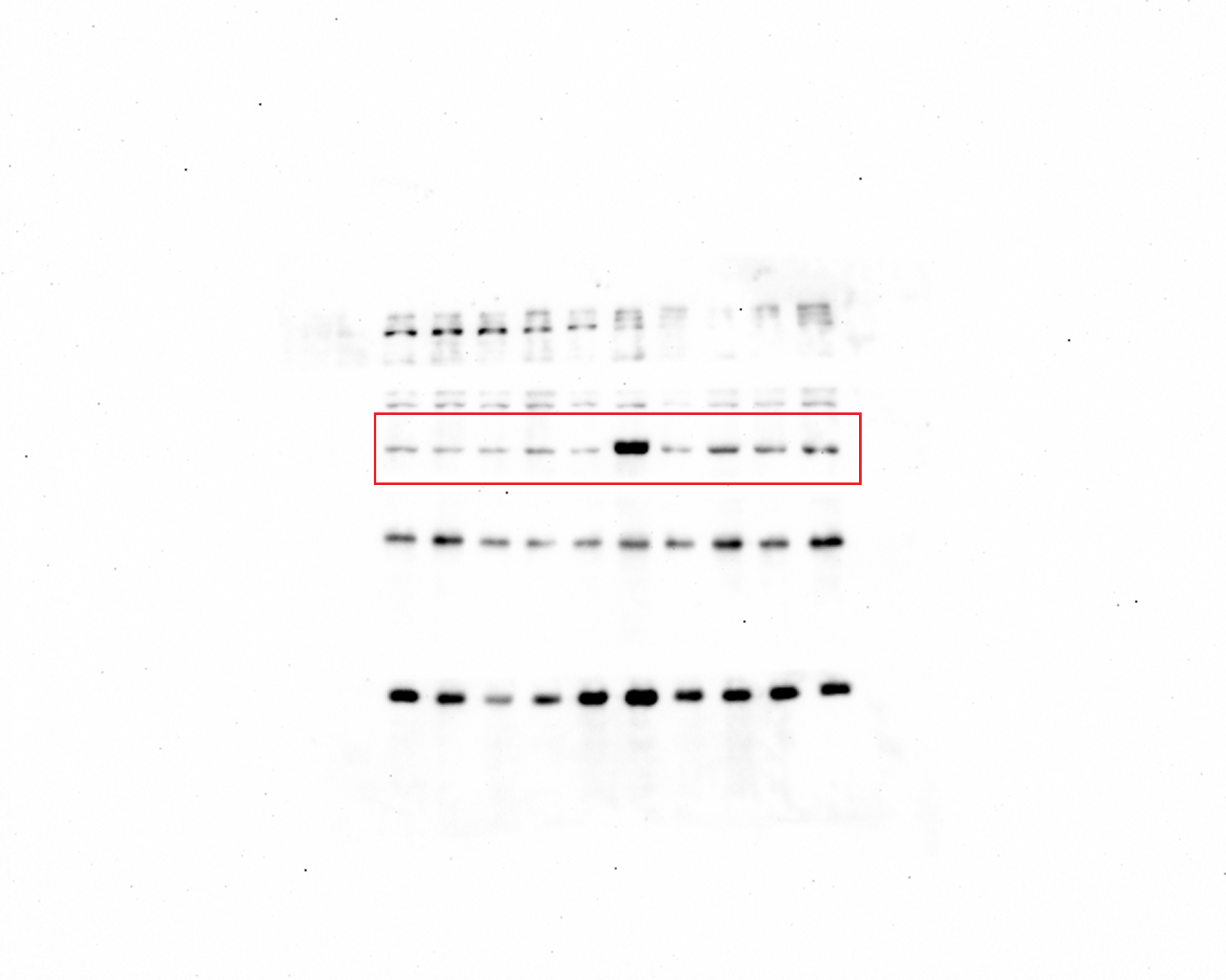

Supplement: Supplementary file 10 — Source Data for Figure 2 [file EMBR-24-e56327-s006.zip › Fig 2/Fig 2a/PKRph.tif]

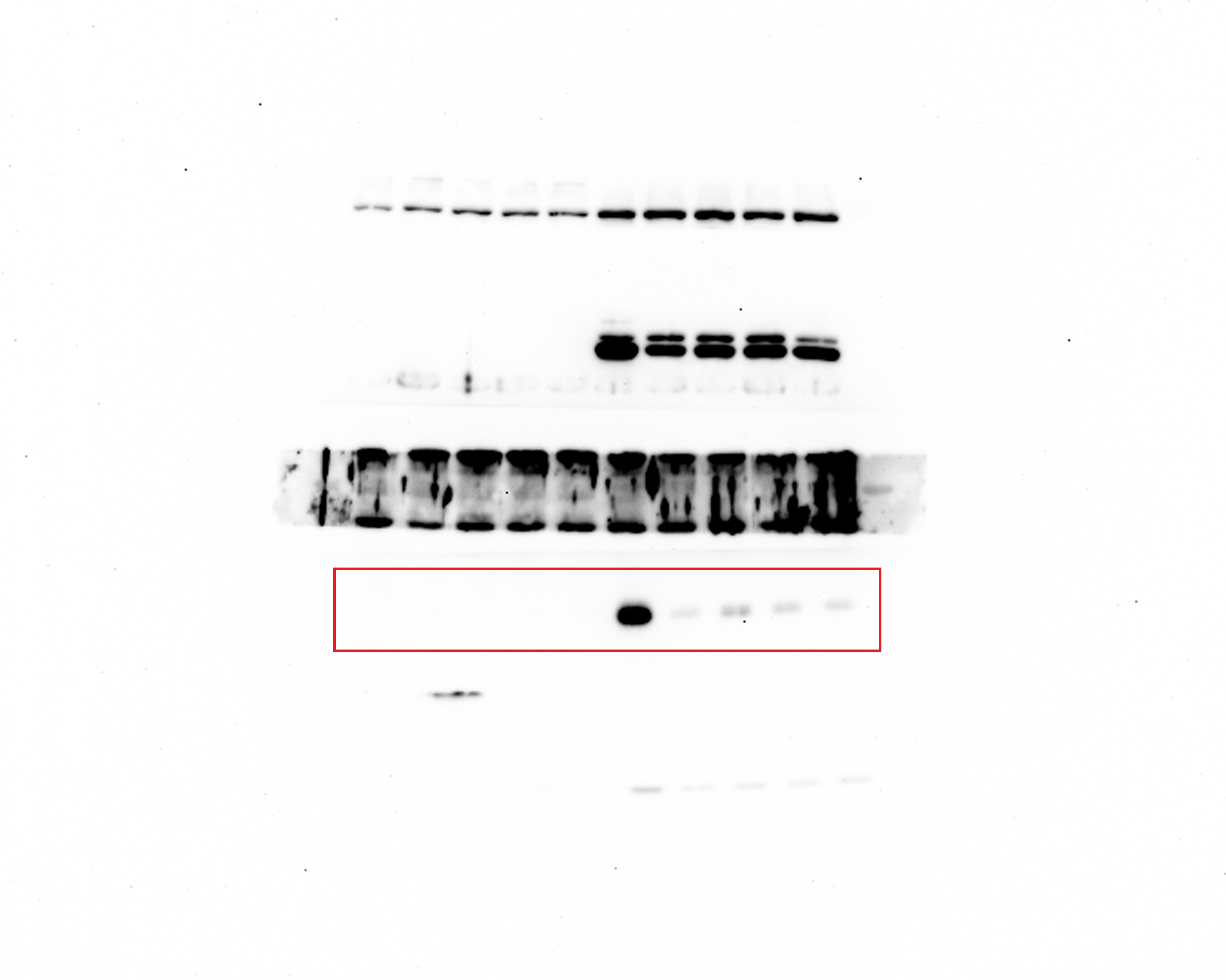

Supplement: Supplementary file 10 — Source Data for Figure 2 [file EMBR-24-e56327-s006.zip › Fig 2/Fig 2a/pp28.tif]

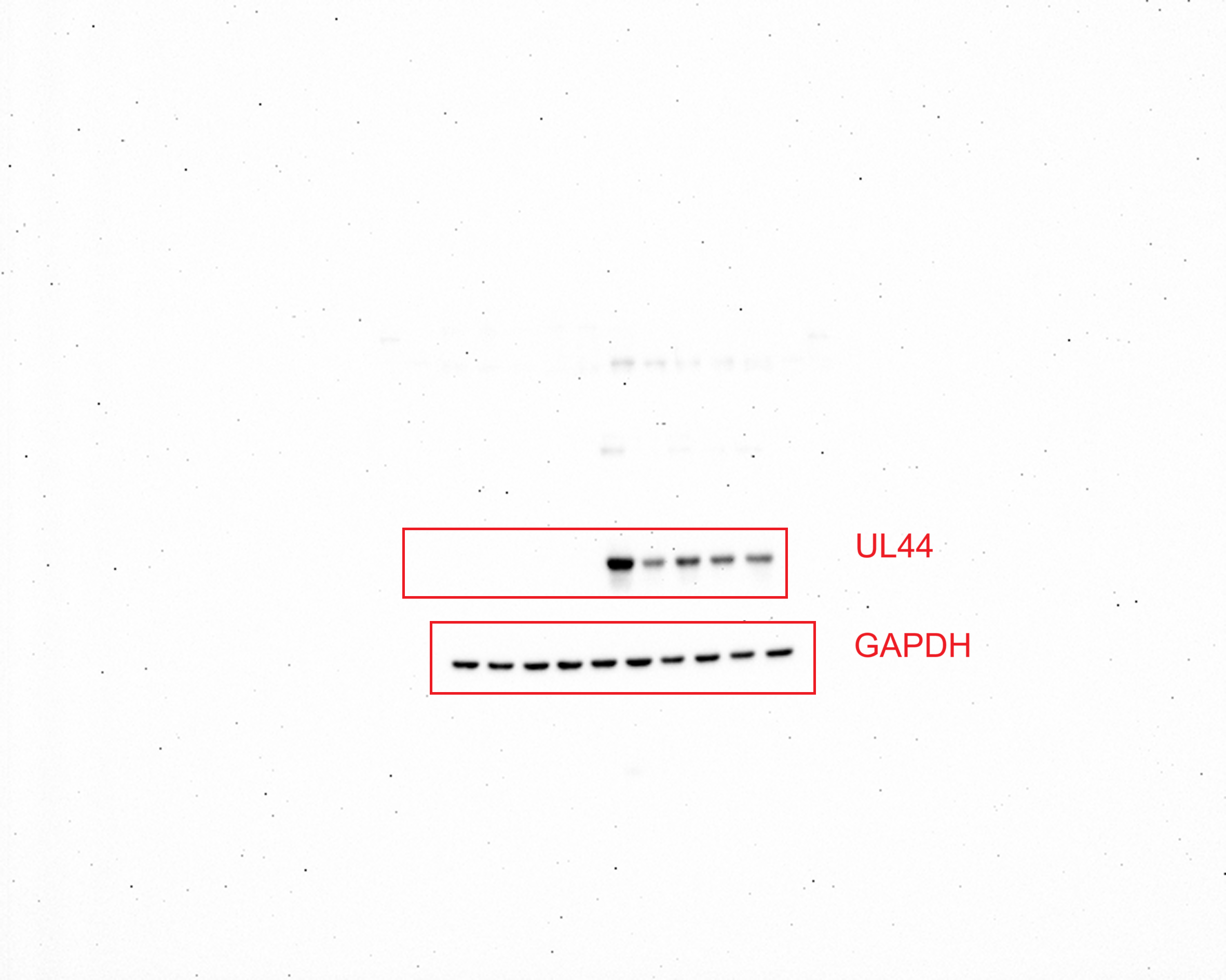

Supplement: Supplementary file 10 — Source Data for Figure 2 [file EMBR-24-e56327-s006.zip › Fig 2/Fig 2a/UL44 GAPDH.tif]

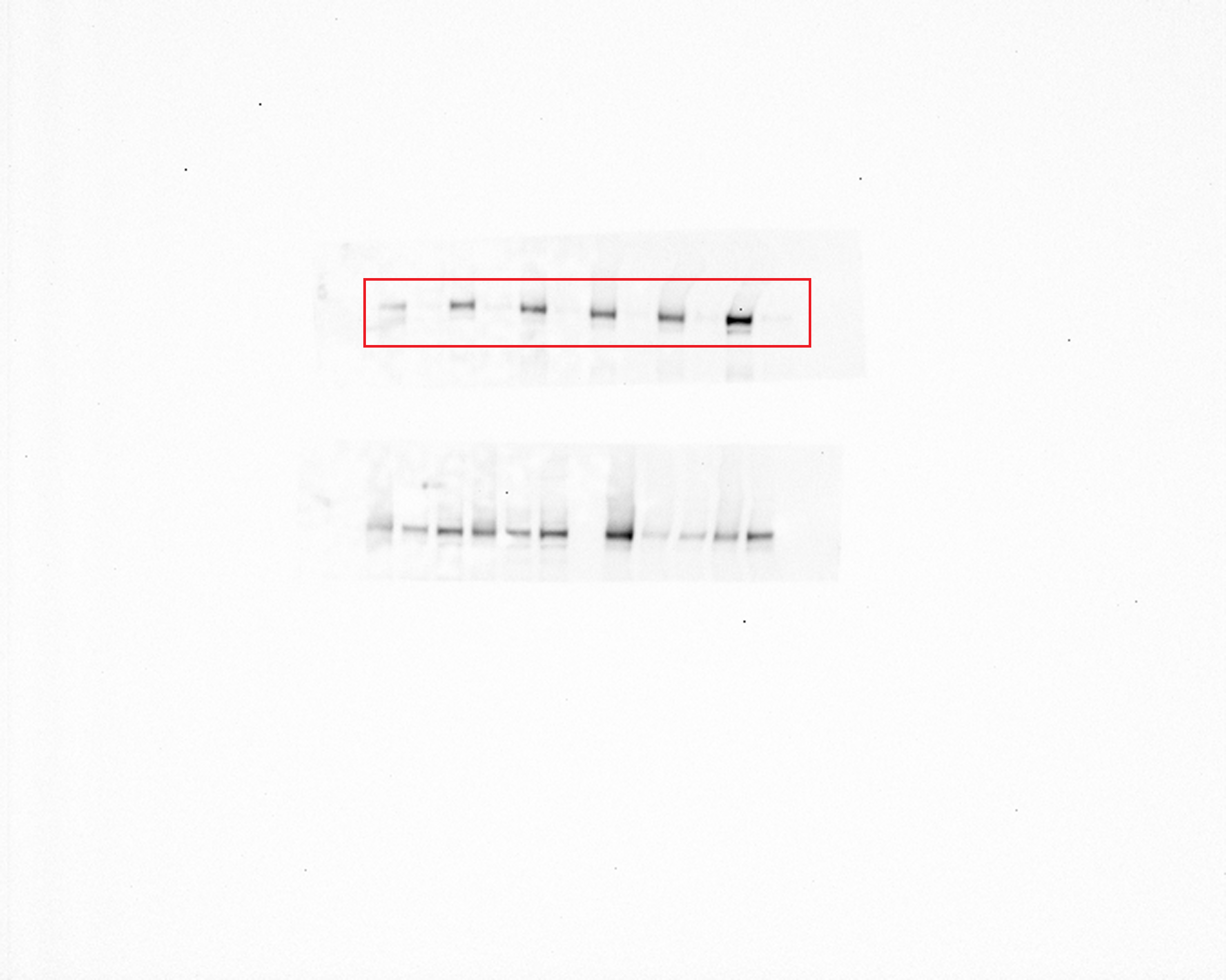

Supplement: Supplementary file 10 — Source Data for Figure 2 [file EMBR-24-e56327-s006.zip › Fig 2/Fig 2c/CNOT1.tif]

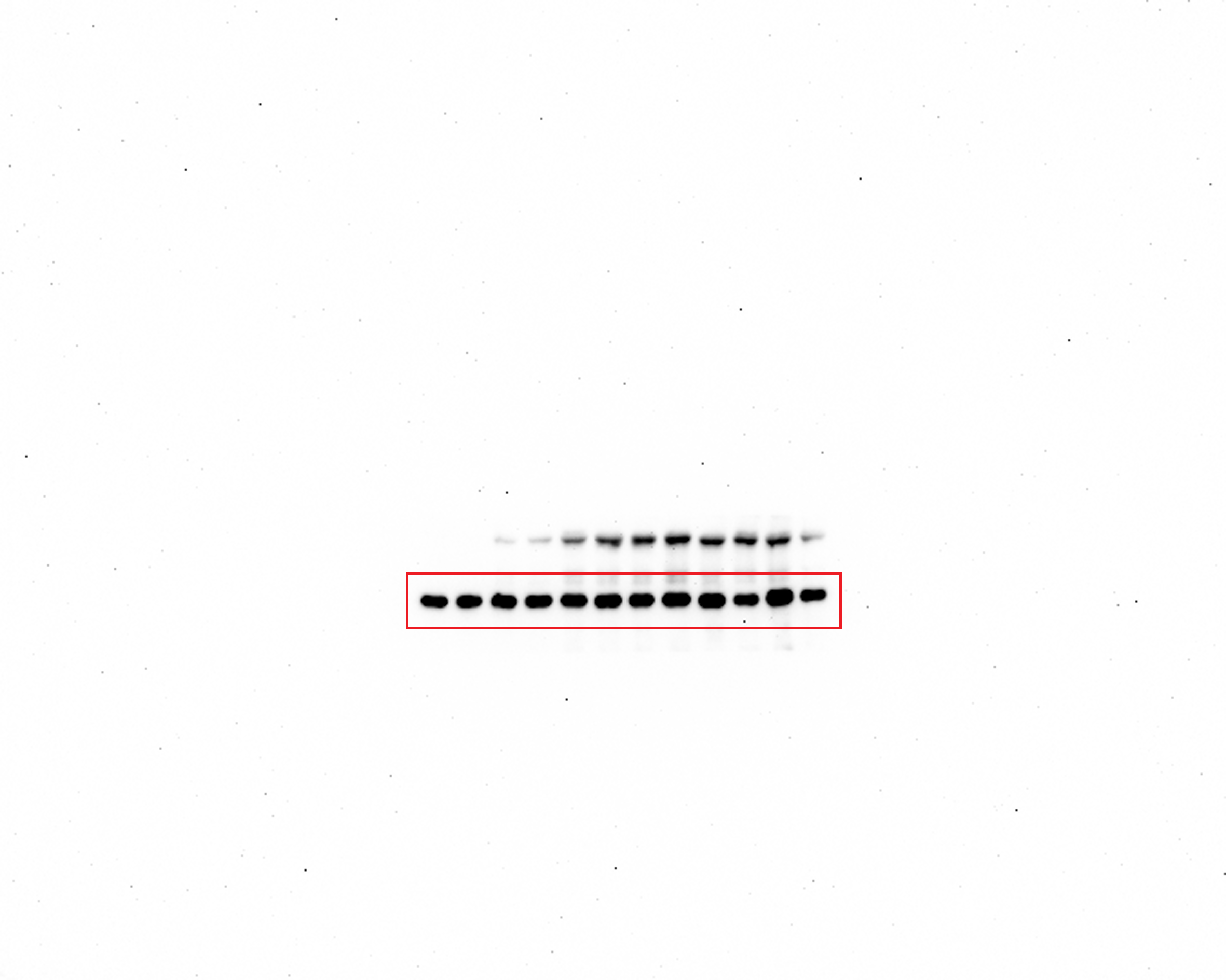

Supplement: Supplementary file 10 — Source Data for Figure 2 [file EMBR-24-e56327-s006.zip › Fig 2/Fig 2c/GAPDH.tif]

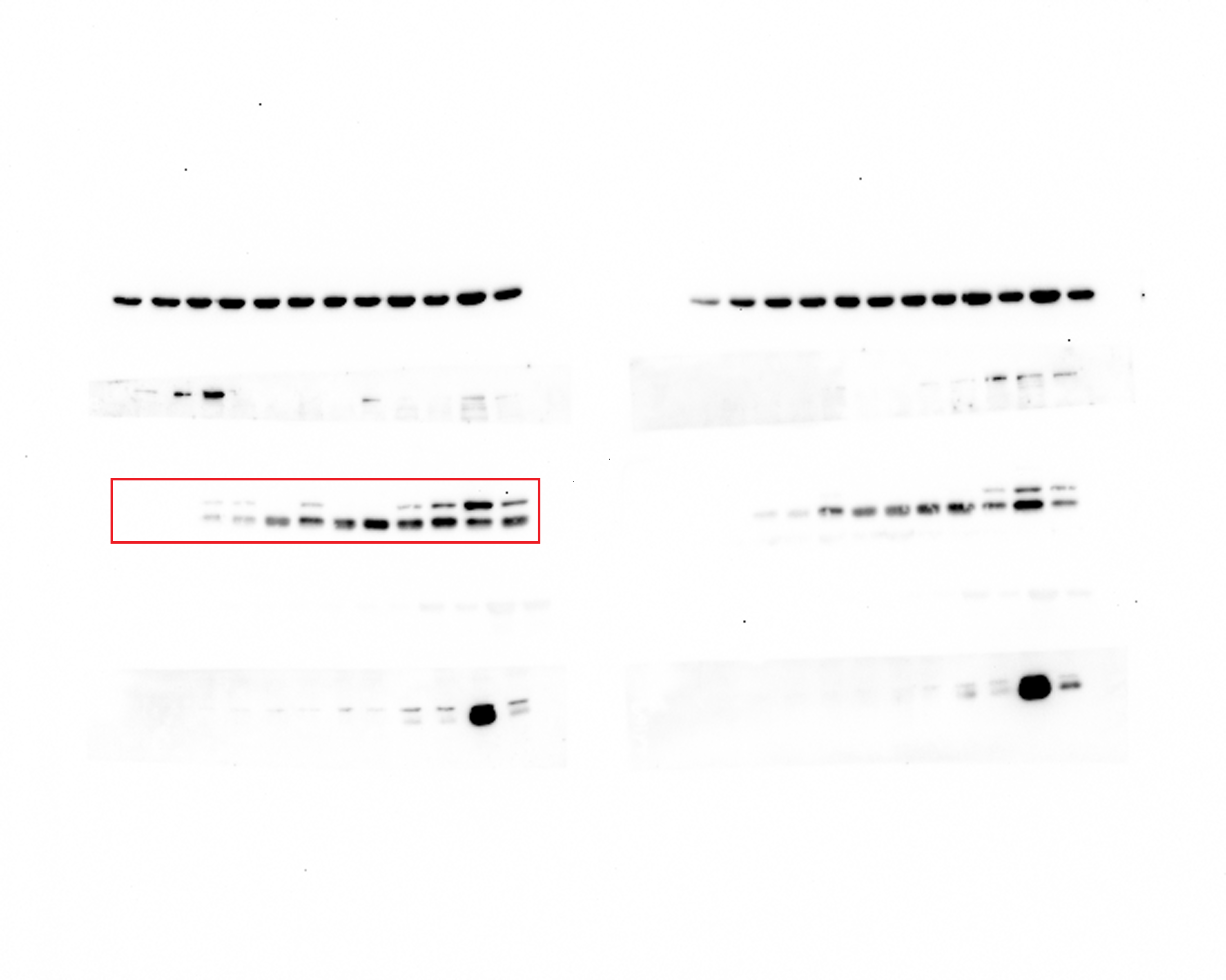

Supplement: Supplementary file 10 — Source Data for Figure 2 [file EMBR-24-e56327-s006.zip › Fig 2/Fig 2c/IE12.tif]

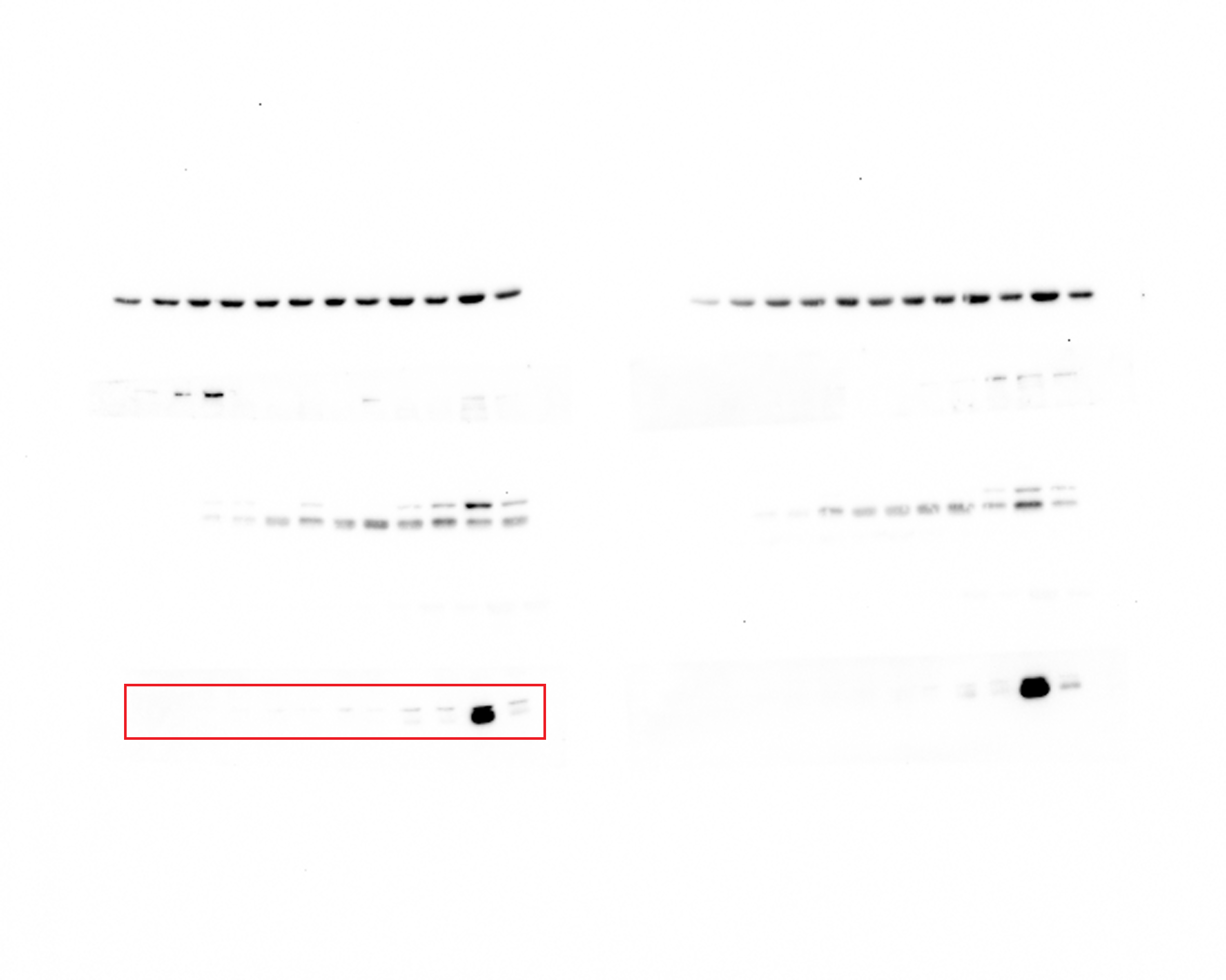

Supplement: Supplementary file 10 — Source Data for Figure 2 [file EMBR-24-e56327-s006.zip › Fig 2/Fig 2c/pp28.tif]

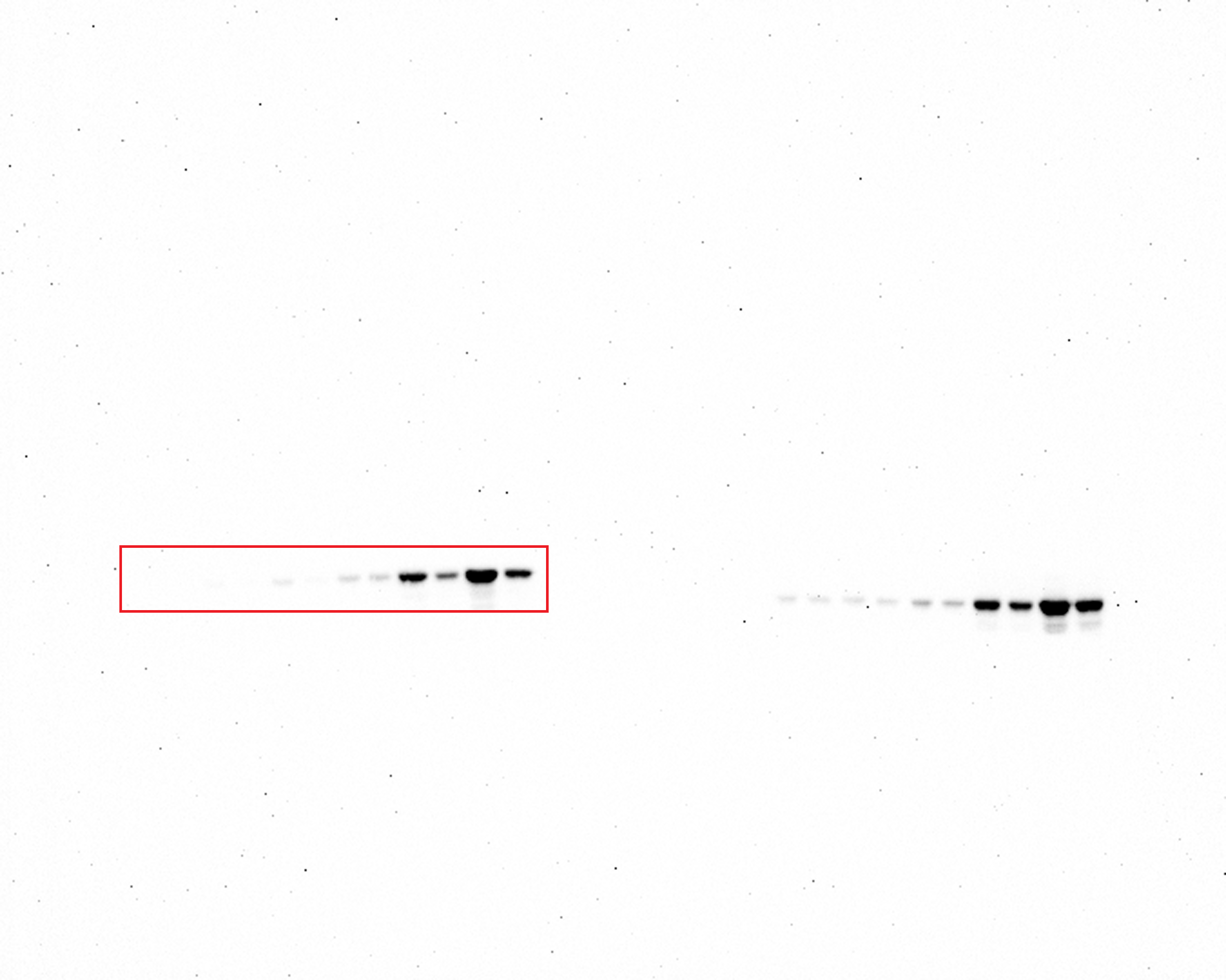

Supplement: Supplementary file 10 — Source Data for Figure 2 [file EMBR-24-e56327-s006.zip › Fig 2/Fig 2c/UL44.tif]

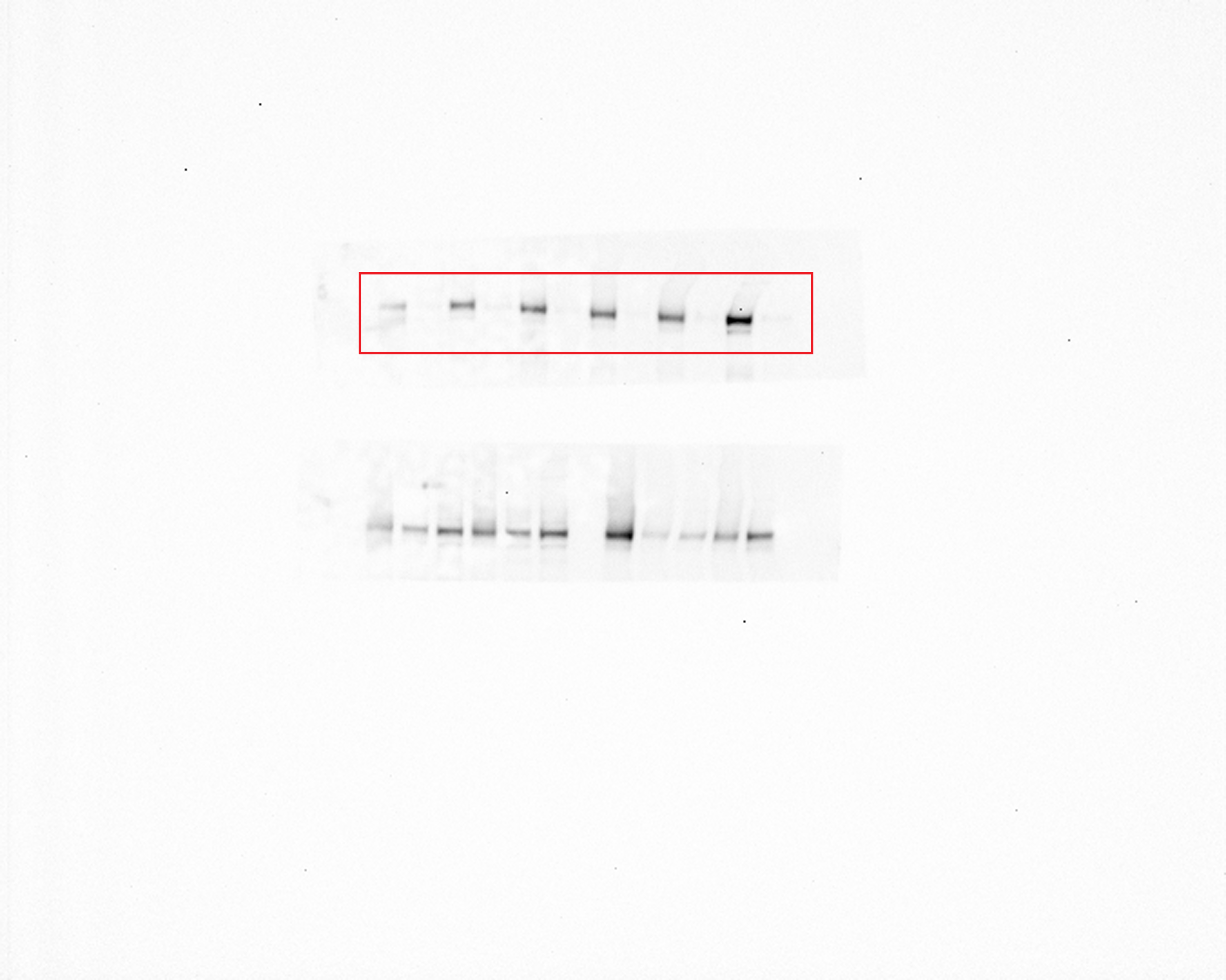

Supplement: Supplementary file 10 — Source Data for Figure 2 [file EMBR-24-e56327-s006.zip › Fig 2/Fig 2d/CNOT1.tif]

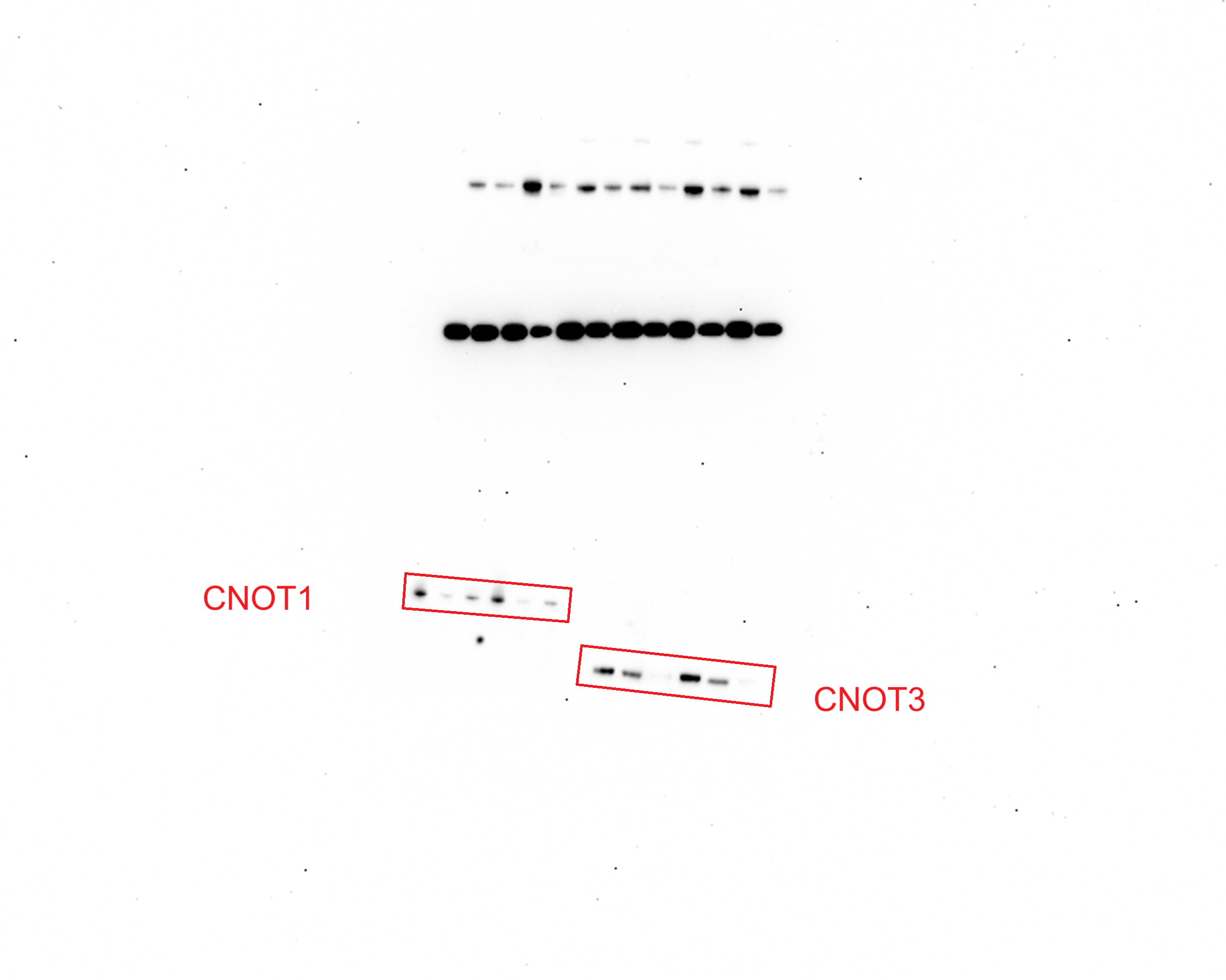

Supplement: Supplementary file 11 — Source Data for Figure 3 [file EMBR-24-e56327-s002.zip › Fig 3/Fig 3a/CNOT1 CNOT3.tif]

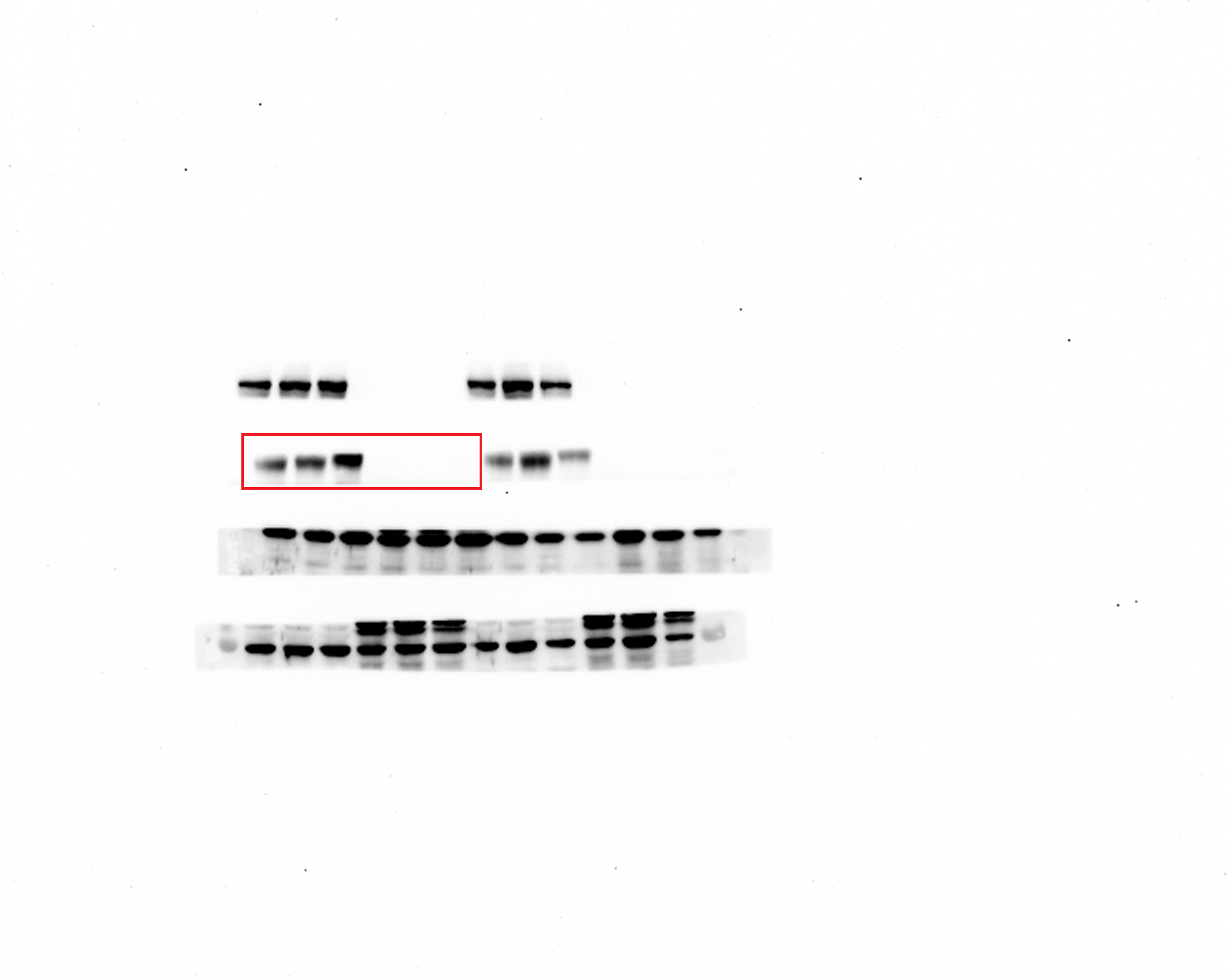

Supplement: Supplementary file 11 — Source Data for Figure 3 [file EMBR-24-e56327-s002.zip › Fig 3/Fig 3a/gC.tif]

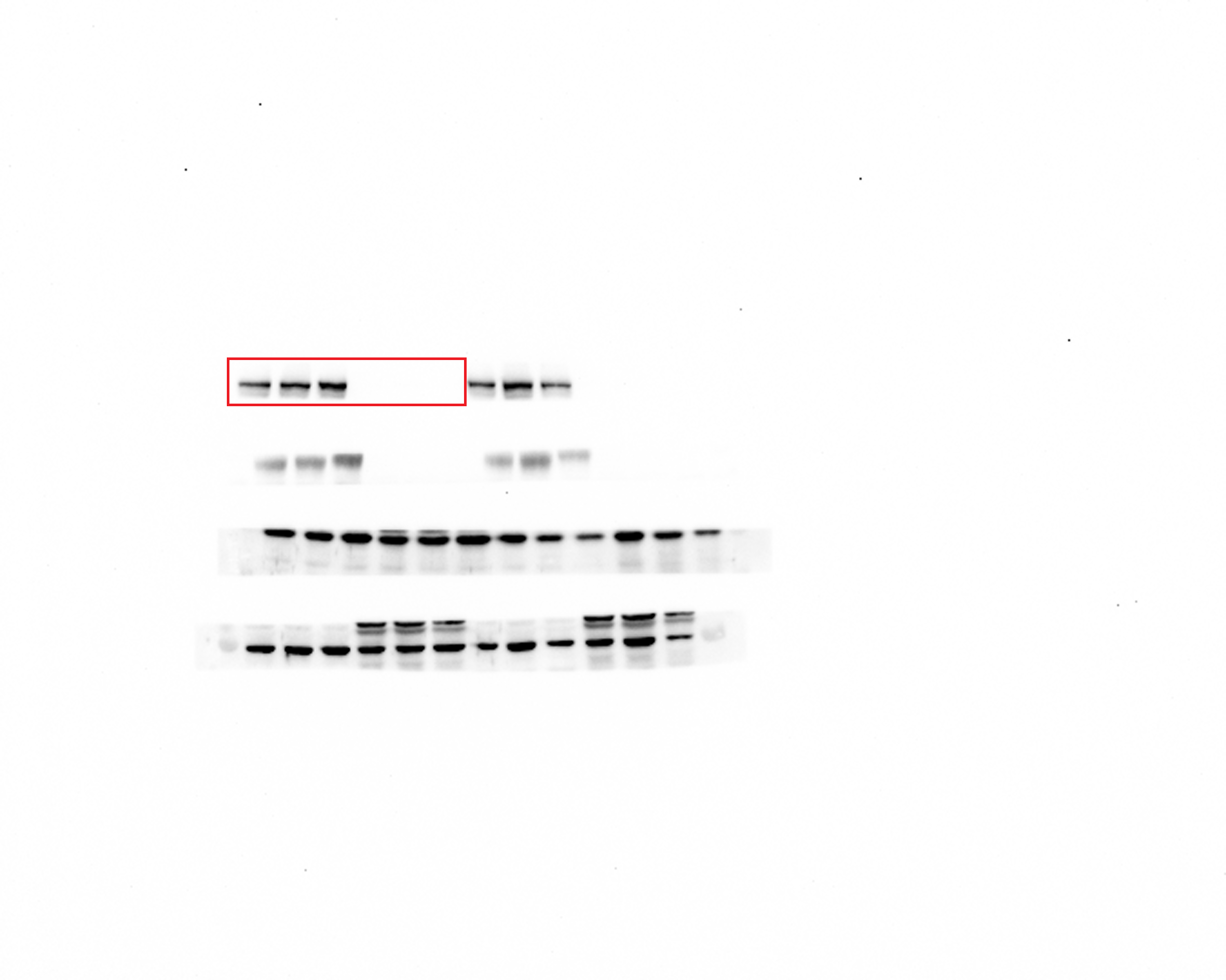

Supplement: Supplementary file 11 — Source Data for Figure 3 [file EMBR-24-e56327-s002.zip › Fig 3/Fig 3a/ICP4.tif]

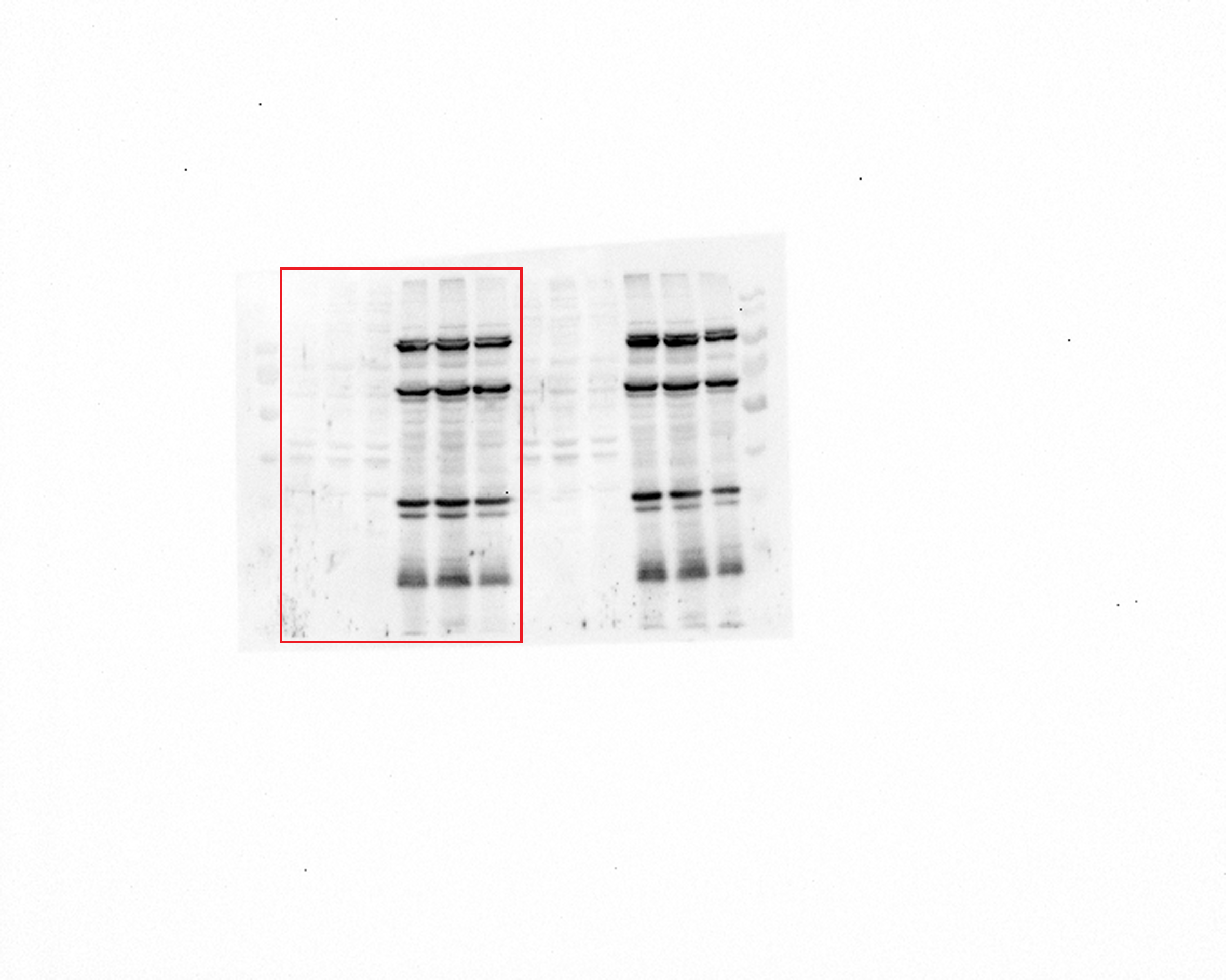

Supplement: Supplementary file 11 — Source Data for Figure 3 [file EMBR-24-e56327-s002.zip › Fig 3/Fig 3a/pan-VACV.tif]

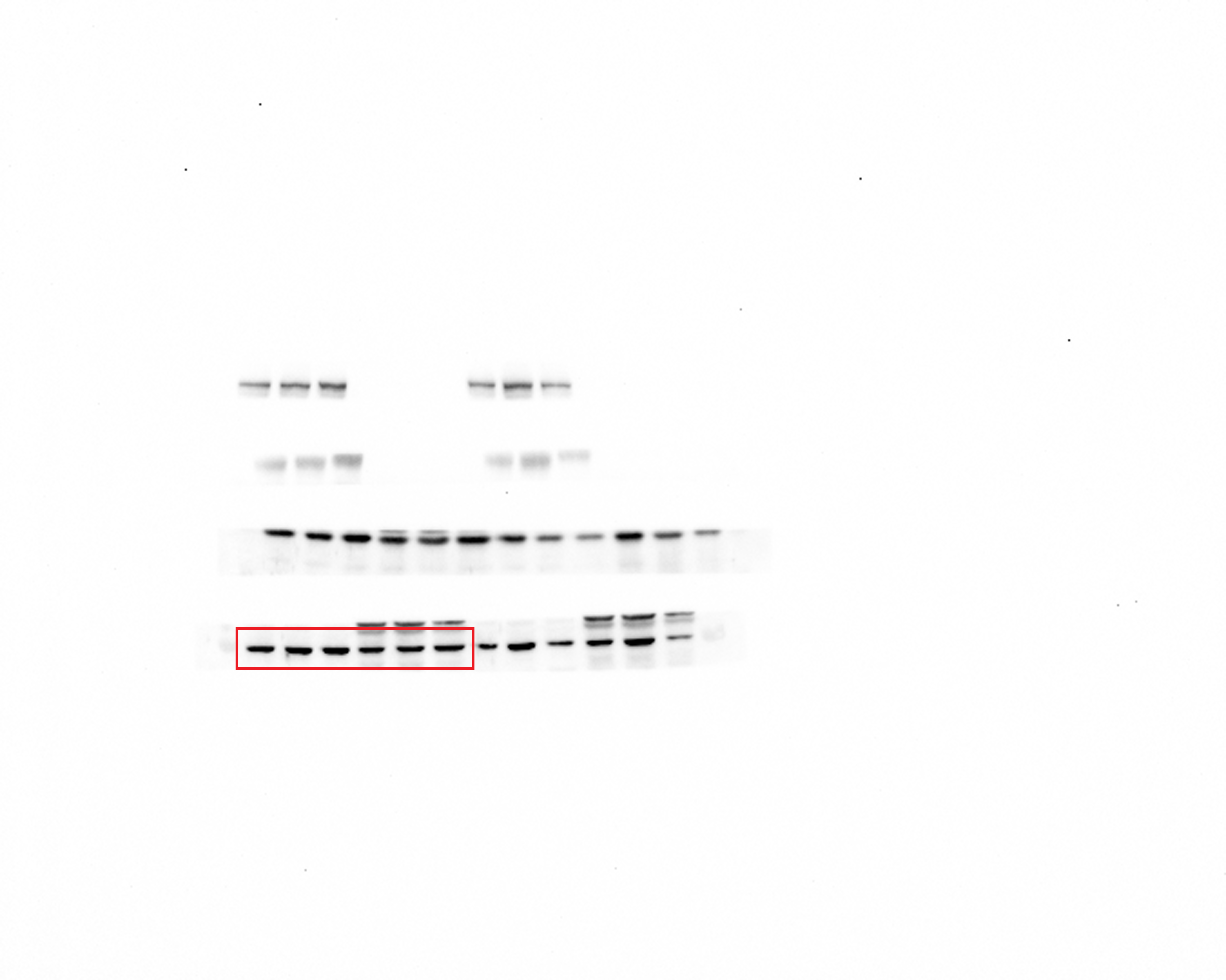

Supplement: Supplementary file 11 — Source Data for Figure 3 [file EMBR-24-e56327-s002.zip › Fig 3/Fig 3a/tubulin.tif]

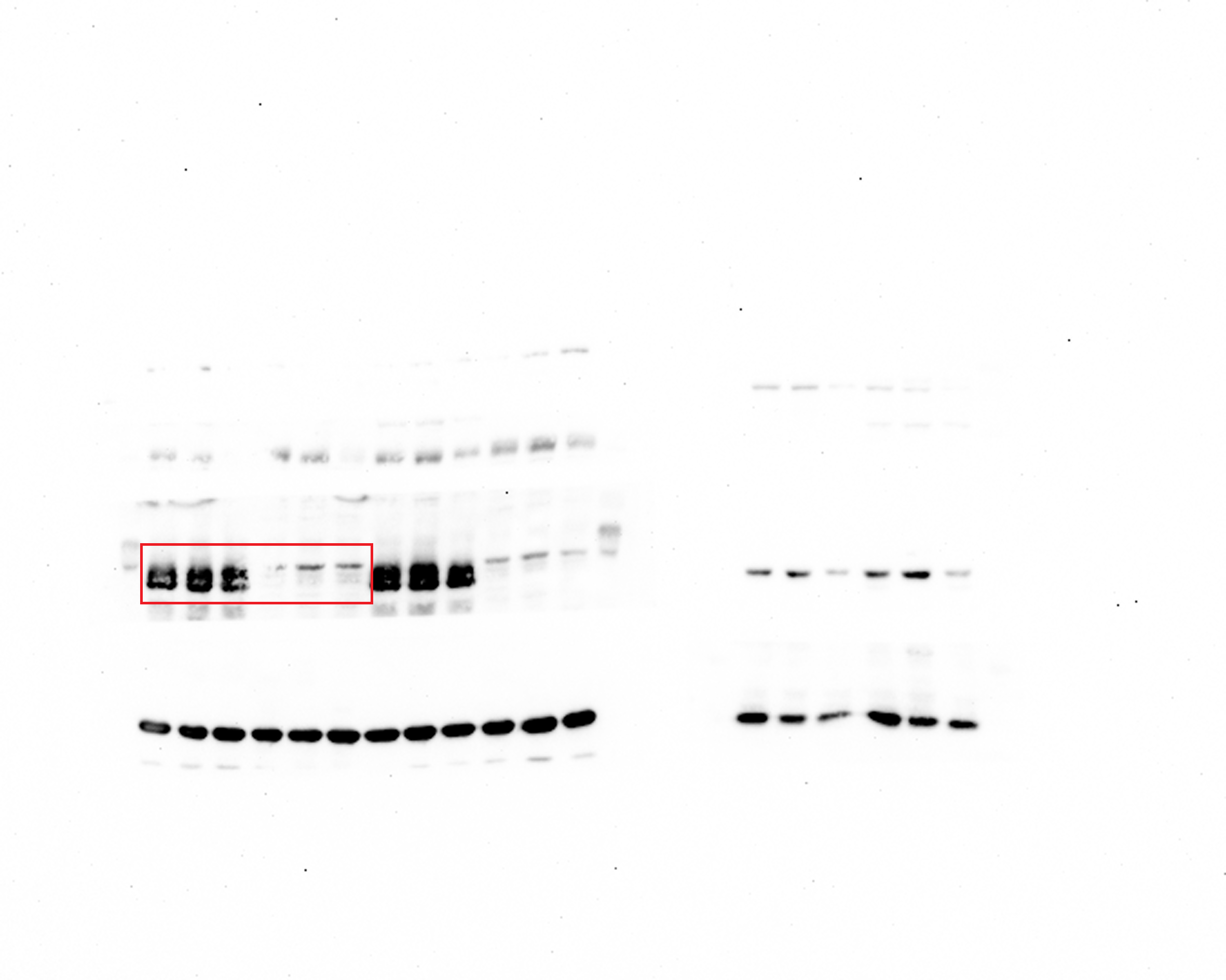

Supplement: Supplementary file 11 — Source Data for Figure 3 [file EMBR-24-e56327-s002.zip › Fig 3/Fig 3a/US3.tif]

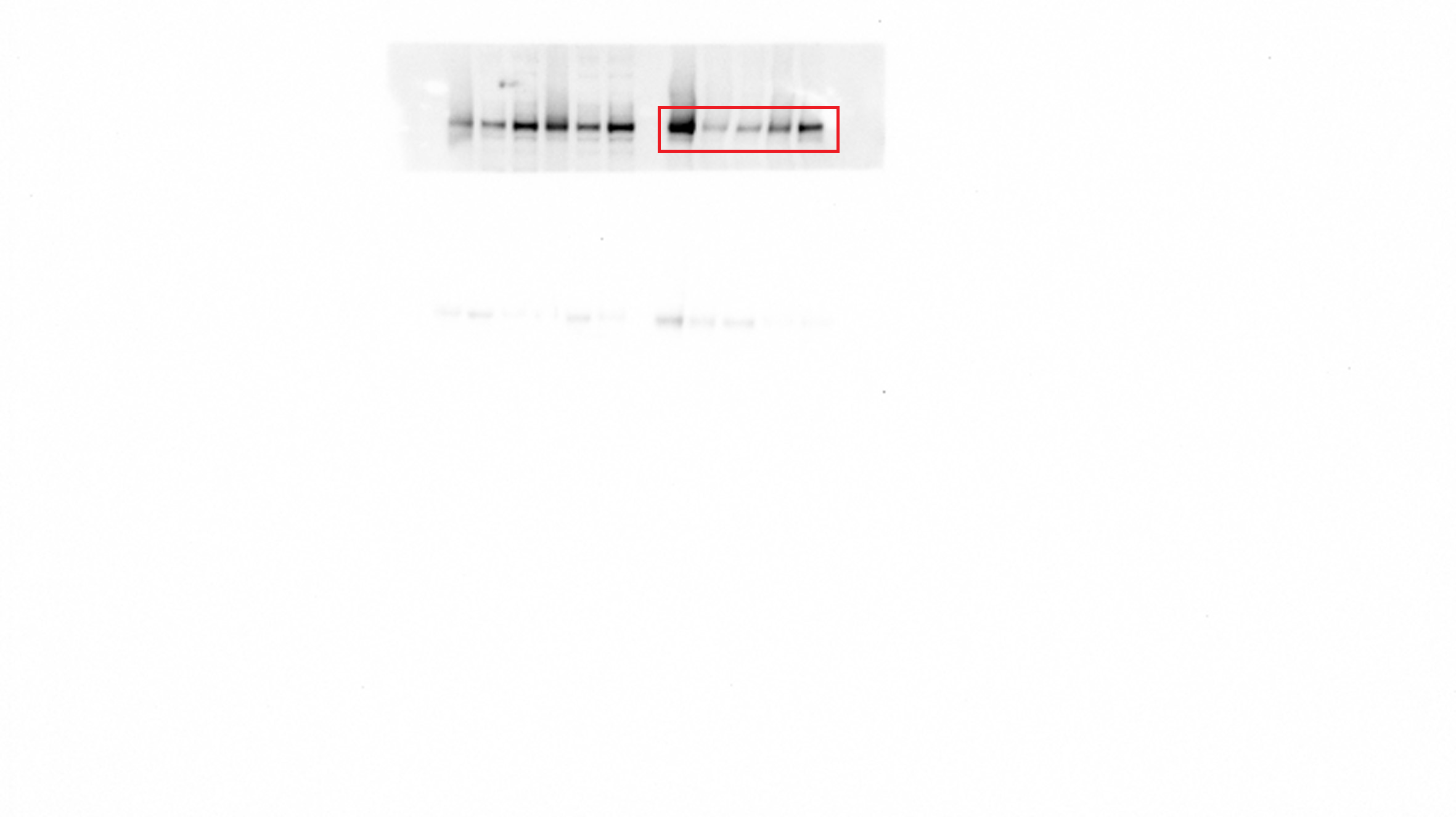

Supplement: Supplementary file 11 — Source Data for Figure 3 [file EMBR-24-e56327-s002.zip › Fig 3/Fig 3b/CNOT1.tif]

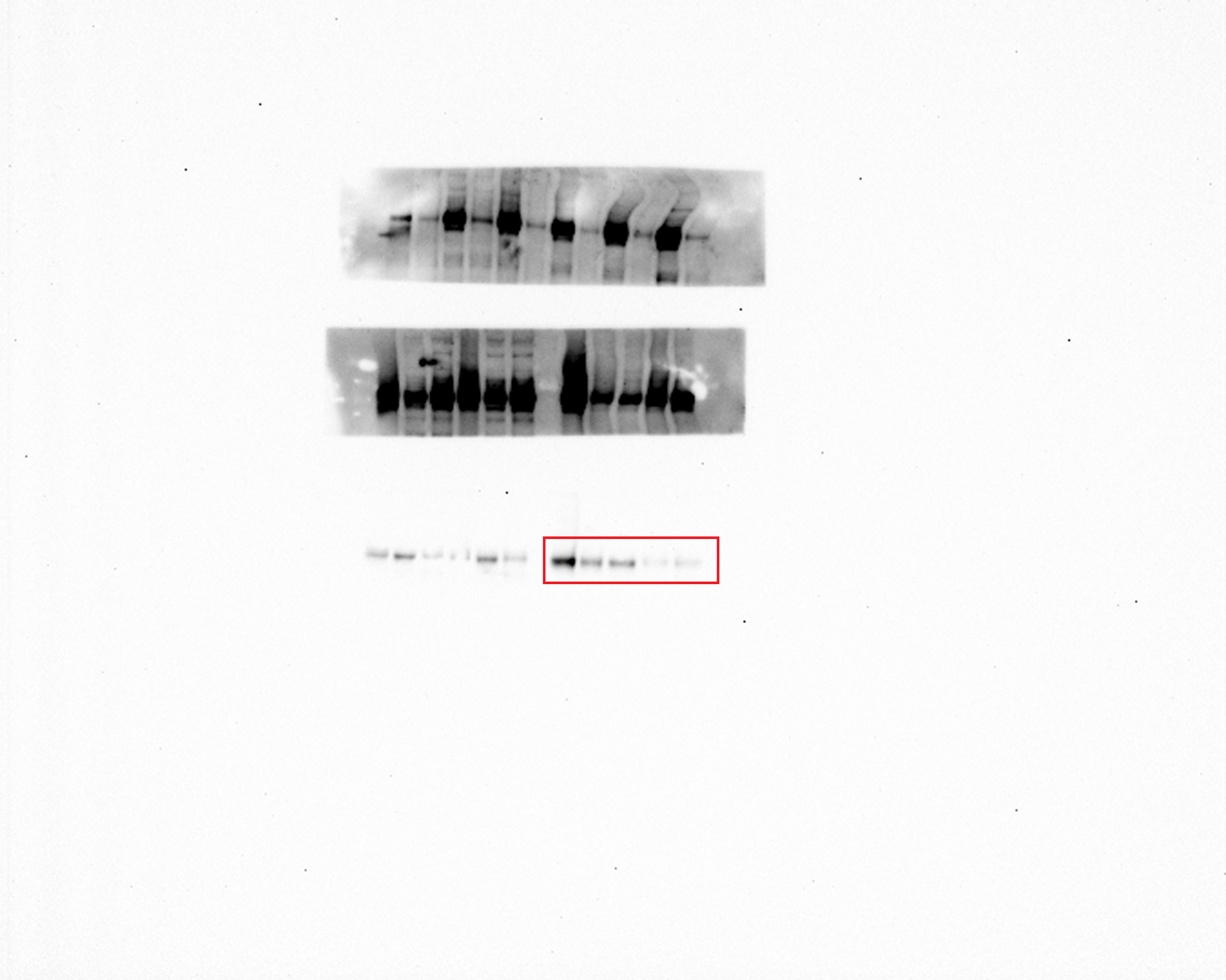

Supplement: Supplementary file 11 — Source Data for Figure 3 [file EMBR-24-e56327-s002.zip › Fig 3/Fig 3b/CNOT3.tif]

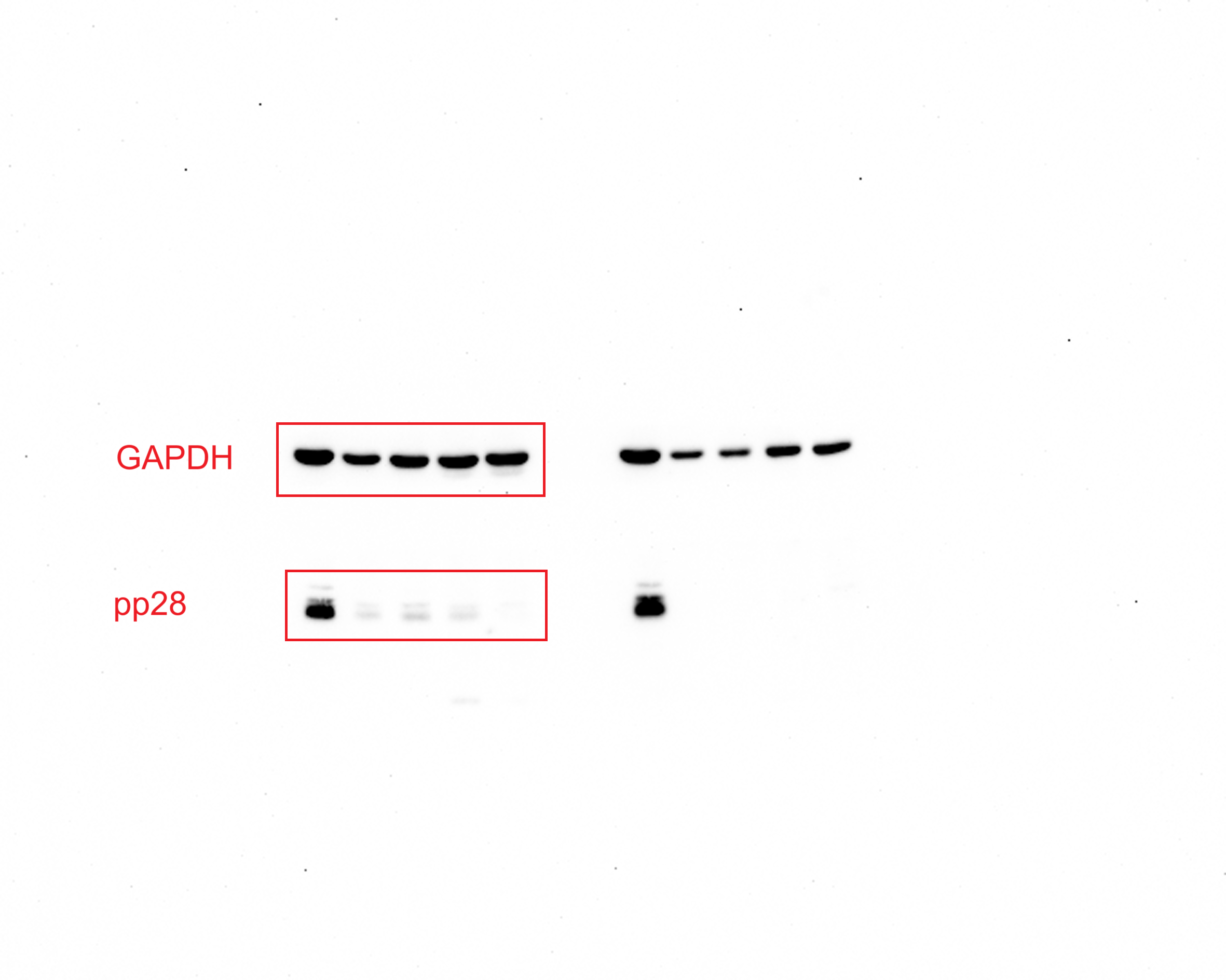

Supplement: Supplementary file 11 — Source Data for Figure 3 [file EMBR-24-e56327-s002.zip › Fig 3/Fig 3b/GAPDH pp28.tif]

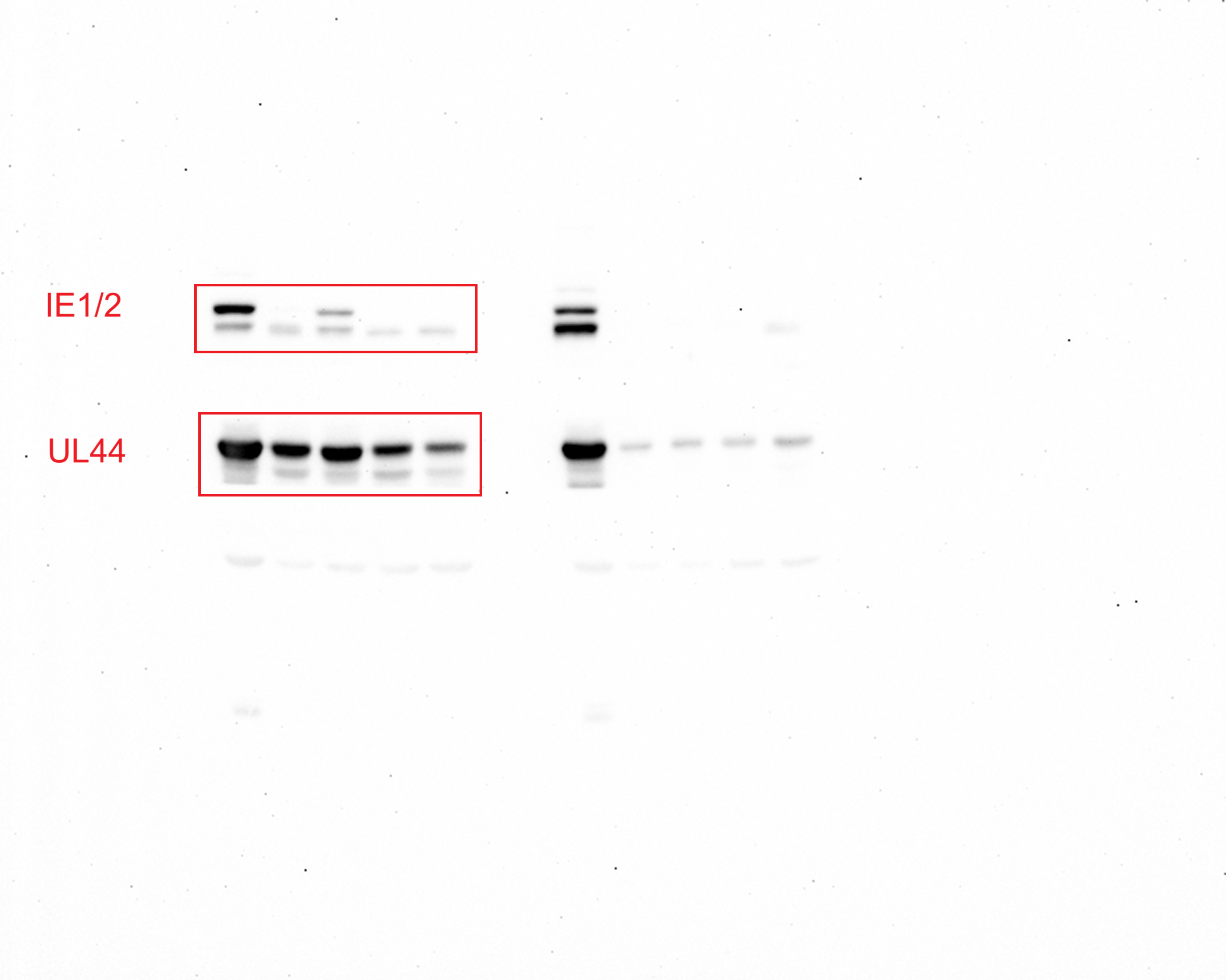

Supplement: Supplementary file 11 — Source Data for Figure 3 [file EMBR-24-e56327-s002.zip › Fig 3/Fig 3b/IE12 UL44.tif]

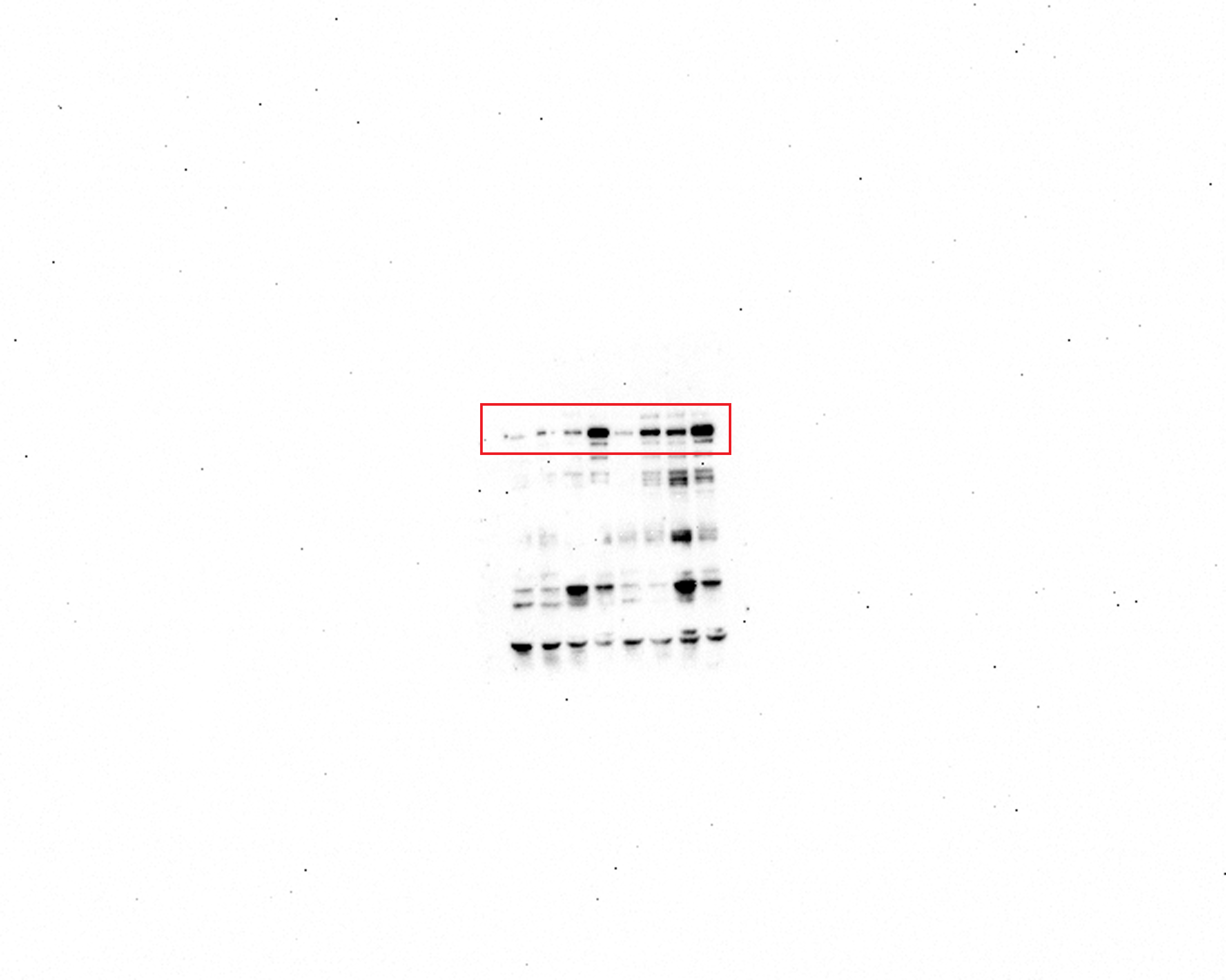

Supplement: Supplementary file 12 — Source Data for Figure 4 [file EMBR-24-e56327-s004.zip › Fig 4/Fig 4a/CNOT1.tif]

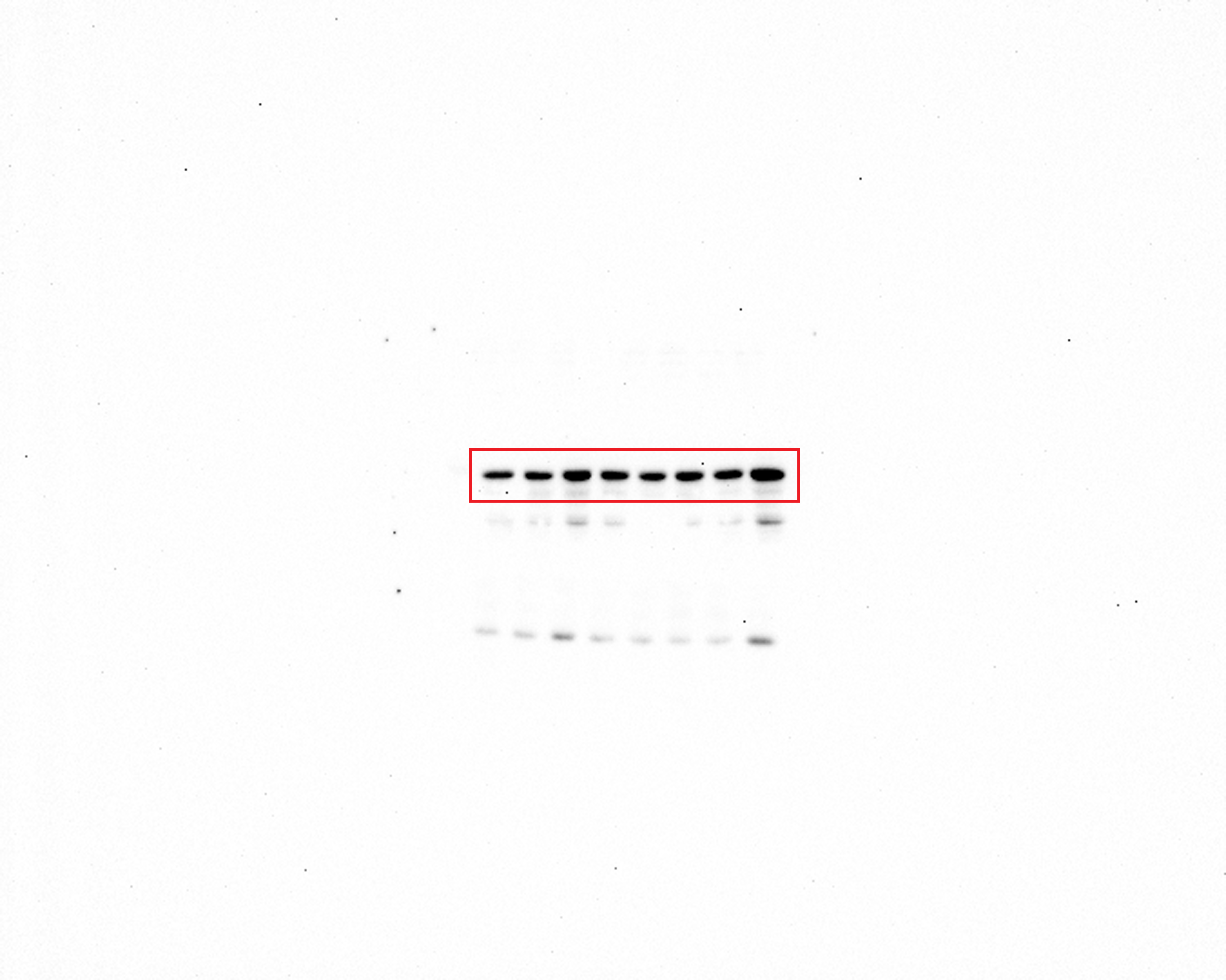

Supplement: Supplementary file 12 — Source Data for Figure 4 [file EMBR-24-e56327-s004.zip › Fig 4/Fig 4a/CNOT2.tif]

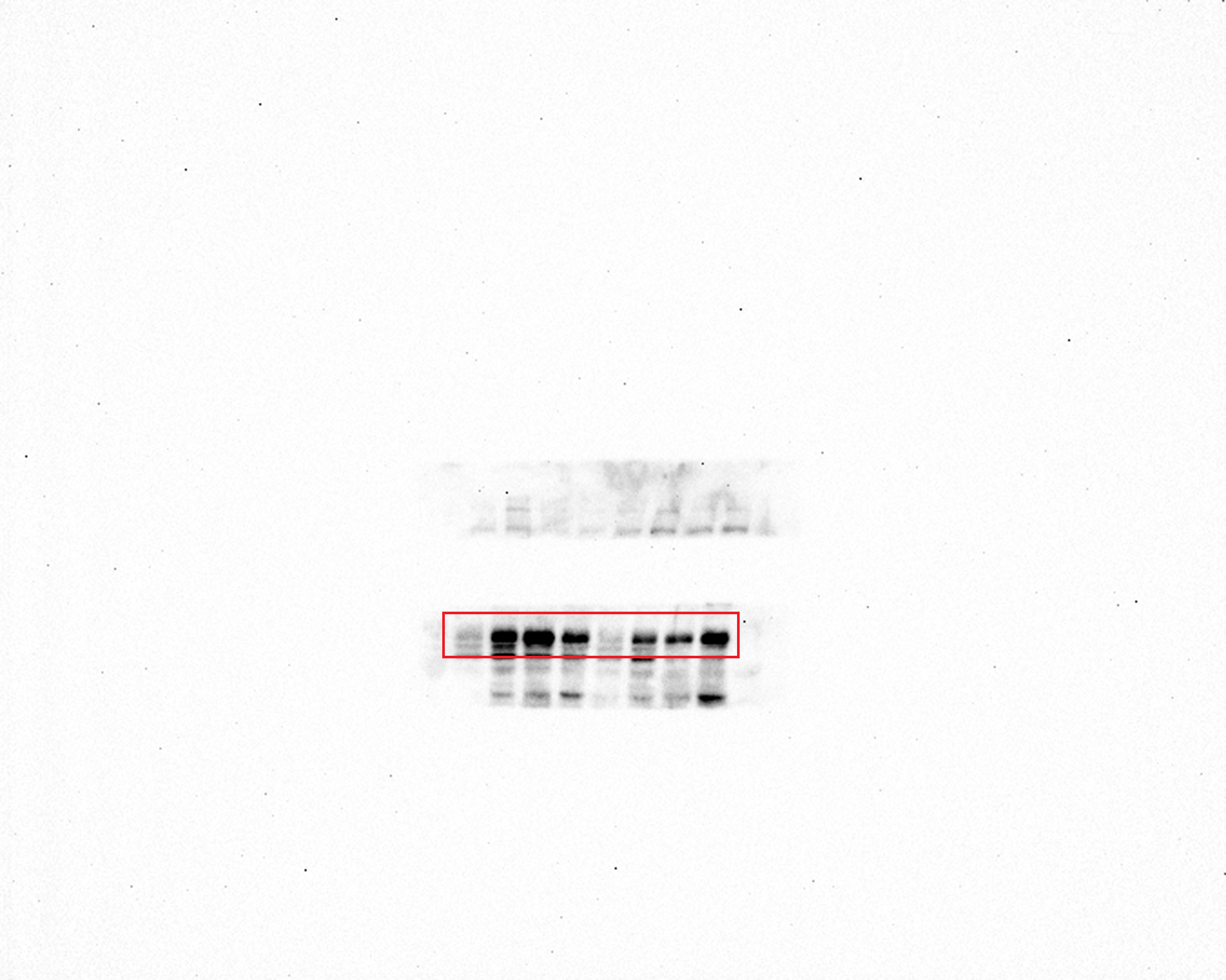

Supplement: Supplementary file 12 — Source Data for Figure 4 [file EMBR-24-e56327-s004.zip › Fig 4/Fig 4a/CNOT3.tif]

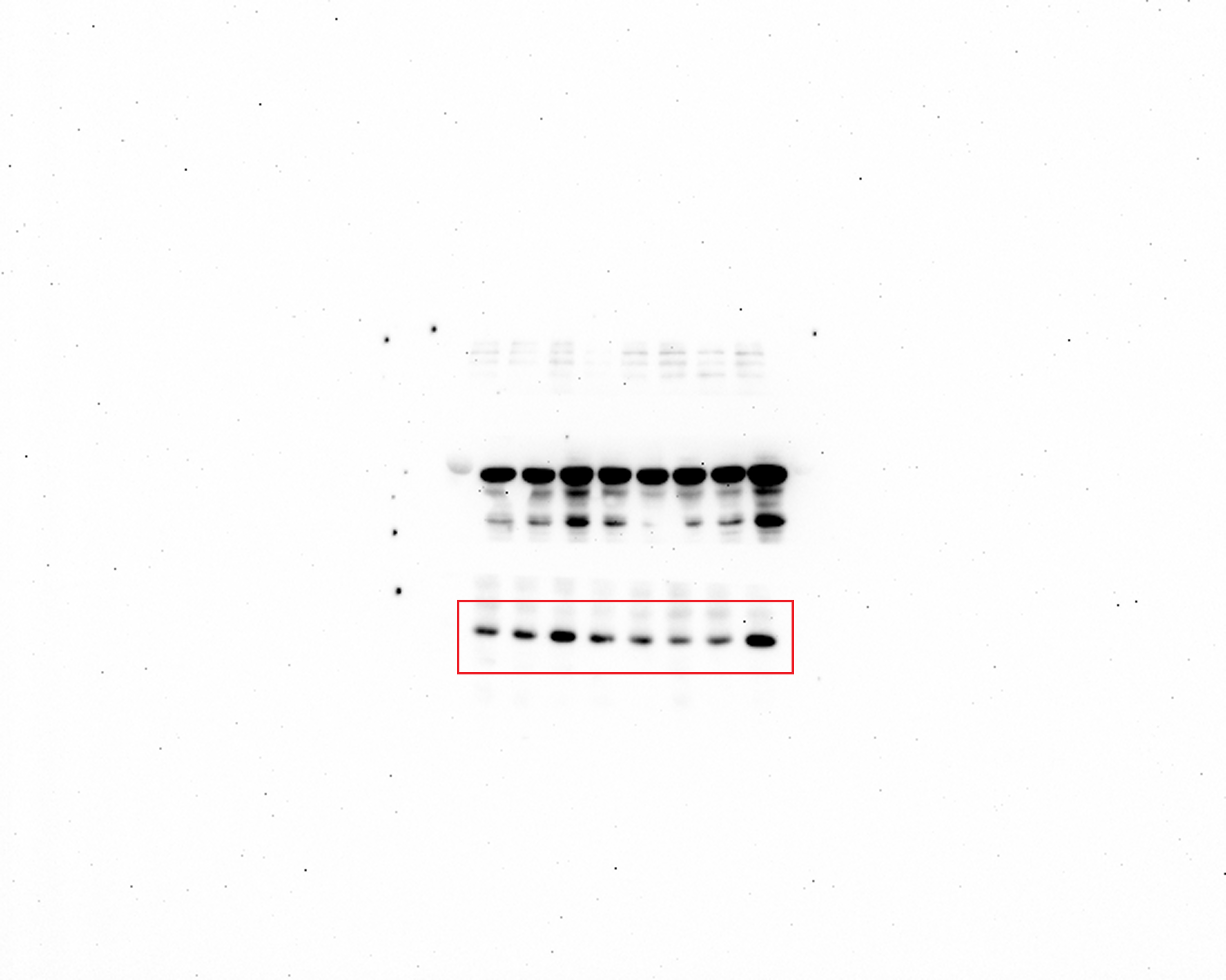

Supplement: Supplementary file 12 — Source Data for Figure 4 [file EMBR-24-e56327-s004.zip › Fig 4/Fig 4a/CNOT7.tif]

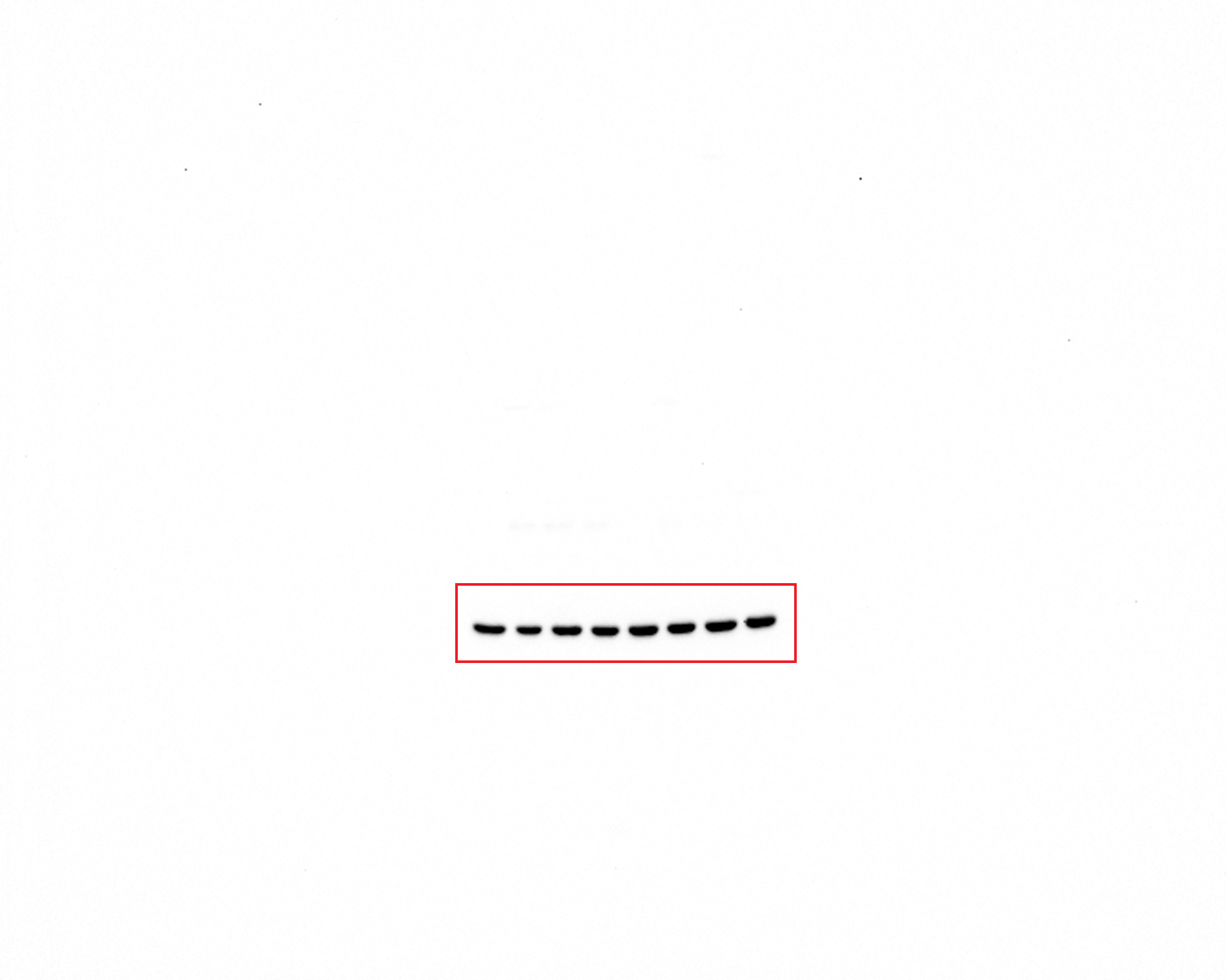

Supplement: Supplementary file 12 — Source Data for Figure 4 [file EMBR-24-e56327-s004.zip › Fig 4/Fig 4a/GAPDH.tif]

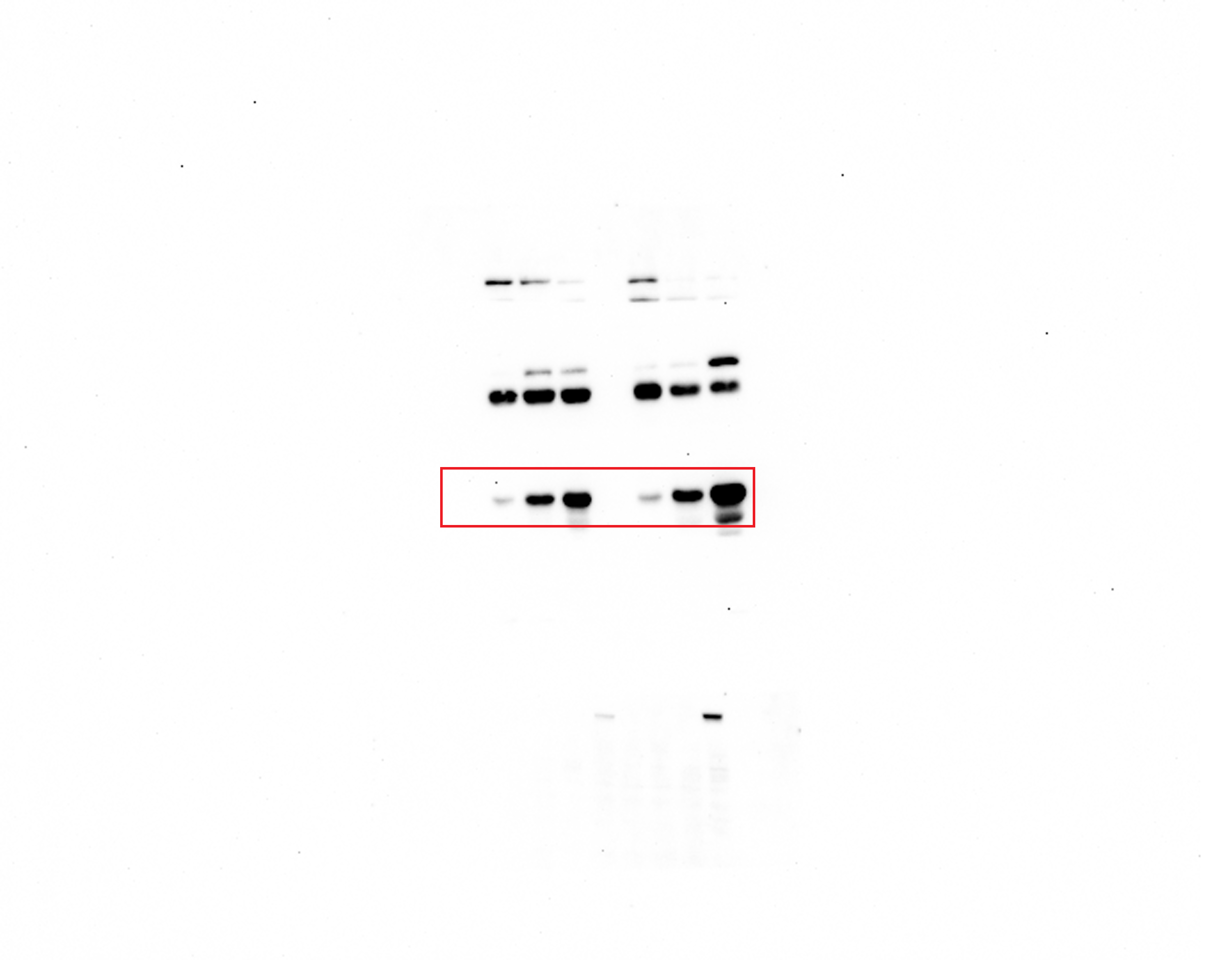

Supplement: Supplementary file 12 — Source Data for Figure 4 [file EMBR-24-e56327-s004.zip › Fig 4/Fig 4a/UL44.tif]
