# Supplementary material for: CCR4‐NOT differentially controls host versus virus poly(a)‐tail length and regulates HCMV infection
Source: EMBO Rep. 2023 Oct 17;24(12):e56327. doi: 10.15252/embr.202256327 (PMC10702830; doi:10.15252/embr.202256327)
Supplement: Supplementary file 15 [file EMBR-24-e56327-s010.pdf]

EMBO reports

CCR4-NOT differentially controls host versus virus poly(A)-tail length and regulates HCMV infection

Rebecca Grande, Sofia Riccio, Ikshitaa Dinesh, Sebastiaan Winkler, Daniel Depledge, Ian Mohr and Hannah Burgess

DOI: 10.15252/embr.202256327

Corresponding author(s): Hannah Burgess (h.burgess@surrey.ac.uk)

Review Timeline:

Submission Date:	18th Oct 22
Editorial Decision:	14th Nov 22
Revision Received:	15th Aug 23
Editorial Decision:	14th Sep 23
Revision Received:	25th Sep 23
Accepted:	28th Sep 23

Editor: Achim Breiling

Transaction Report:

(Note: With the exception of the correction of typographical or spelling errors that could be a source of ambiguity, letters and reports are not edited. Depending on transfer agreements, referee reports obtained elsewhere may or may not be included in this compilation. Referee reports are anonymous unless the Referee chooses to sign their reports.)

1st Editorial Decision 14th Nov 2022

Dear Dr. Burgess,

Thank you for the submission of your research manuscript to EMBO reports. I have now received the reports from the three referees that were asked to evaluate your study, which can be found at the end of this email.

As you will see, the referees think that these findings are of interest. However, they have several comments, concerns, and suggestions, indicating that a major revision of the manuscript is necessary to allow publication of the study in EMBO reports. As the reports are below, and all the referee concerns need to be addressed, I will not detail them here.

Given the constructive referee comments, we would like to invite you to revise your manuscript with the understanding that all referee concerns must be addressed in the revised manuscript and in a detailed point-by-point response. Acceptance of your manuscript will depend on a positive outcome of a second round of review. It is EMBO reports policy to allow a single round of revision only and acceptance of the manuscript will therefore depend on the completeness of your responses included in the next, final version of the manuscript.

Revised manuscripts should be submitted within three months of a request for revision. Please contact me to discuss the revision (also by video chat) if you have questions or comments regarding the revision, or should you need additional time.

When submitting your revised manuscript, please also carefully review the instructions that follow below.

PLEASE NOTE THAT upon resubmission revised manuscripts are subjected to an initial quality control prior to exposition to re-review. Upon failure in the initial quality control, the manuscripts are sent back to the authors, which may lead to delays. Frequent reasons for such a failure are the lack of the data availability section (please see below) and the presence of statistics based on n=2 (the authors are then asked to present scatter plots or provide more data points).

When submitting your revised manuscript, we will require:

1) a .docx formatted version of the final manuscript text (including legends for main figures, EV figures and tables), but without the figures included. Figure legends should be compiled at the end of the manuscript text.

2) individual production quality figure files as .eps, .tif, .jpg (one file per figure), of main figures (up to 8) and EV figures. Please upload these as separate, individual files upon re-submission.

The Expanded View format, which will be displayed in the main HTML of the paper in a collapsible format, has replaced the Supplementary information. You can submit up to 5 images as Expanded View. Please follow the nomenclature Figure EV1, Figure EV2 etc. The figure legend for these should be included in the main manuscript document file in a section called Expanded View Figure Legends after the main Figure Legends section. Additional Supplementary material should be supplied as a single pdf file labeled Appendix. The Appendix should have page numbers and needs to include a table of content on the first page (with page numbers) and legends for all content. Please follow the nomenclature Appendix Figure Sx, Appendix Table Sx etc. throughout the text, and also label the figures and tables according to this nomenclature.

For more details, please refer to our guide to authors:
http://www.embopress.org/page/journal/14693178/authorguide#manuscriptpreparation

Please consult our guide for figure preparation:
http://wol-prod-cdn.literatumonline.com/pb-assets/embo-site/EMBOPress_Figure_Guidelines_061115-1561436025777.pdf

See also the guidelines for figure legend preparation:
https://www.embopress.org/page/journal/14693178/authorguide#figureformat

3) a complete author checklist, which you can download from our author guidelines (https://www.embopress.org/page/journal/14693178/authorguide). Please insert page numbers in the checklist to indicate where the requested information can be found in the manuscript. The completed author checklist will also be part of the RPF.

Please also follow our guidelines for the use of living organisms, and the respective reporting guidelines:
http://www.embopress.org/page/journal/14693178/authorguide#livingorganisms

4) that primary datasets produced in this study (e.g. RNA-seq, ChIP-seq, structural and array data) are deposited in an appropriate public database. If no primary datasets have been deposited, please also state this in a dedicated section (e.g. 'No primary datasets have been generated and deposited'), see below.

See also: http://embor.embopress.org/authorguide#datadeposition

Please remember to provide a reviewer password if the datasets are not yet public.

The accession numbers and database should be listed in a formal "Data Availability" section (placed after Materials & Methods) that follows the model below. This is now mandatory (like the COI statement). Please note that the Data Availability Section is restricted to new primary data that are part of this study. This section is mandatory. As indicated above, if no primary datasets have been deposited, please state this in this section

Data availability

The datasets produced in this study are available in the following databases:

- RNA-Seq data: Gene Expression Omnibus GSE46843 (https://www.ncbi.nlm.nih.gov/geo/query/acc.cgi?acc=GSE46843)
- [data type]: [name of the resource] [accession number/identifier/doi] ([URL or identifiers.org/DATABASE:ACCESSION])

*** Note - All links should resolve to a page where the data can be accessed. ***

Moreover, I have these editorial requests:

6) We now request the publication of original source data with the aim of making primary data more accessible and transparent to the reader. Our source data coordinator will contact you to discuss which figure panels we would need source data for and will also provide you with helpful tips on how to upload and organize the files.

7) Our journal encourages inclusion of *data citations in the reference list* to directly cite datasets that were re-used and obtained from public databases. Data citations in the article text are distinct from normal bibliographical citations and should directly link to the database records from which the data can be accessed. In the main text, data citations are formatted as follows: "Data ref: Smith et al, 2001" or "Data ref: NCBI Sequence Read Archive PRJNA342805, 2017". In the Reference list, data citations must be labeled with "[DATASET]". A data reference must provide the database name, accession number/identifiers and a resolvable link to the landing page from which the data can be accessed at the end of the reference. Further instructions are available at: http://www.embopress.org/page/journal/14693178/authorguide#referencesformat

8) Regarding data quantification and statistics, please make sure that the number "n" for how many independent experiments were performed, their nature (biological versus technical replicates), the bars and error bars (e.g. SEM, SD) and the test used to calculate p-values is indicated in the respective figure legends (also for potential EV figures and all those in the final Appendix). Please also check that all the p-values are explained in the legend, and that these fit to those shown in the figure. Please provide statistical testing where applicable. Please avoid the phrase 'independent experiment', but clearly state if these were biological or technical replicates. Please also indicate (e.g. with n.s.) if testing was performed, but the differences are not significant. In case n=2, please show the data as separate datapoints without error bars and statistics. See also: http://www.embopress.org/page/journal/14693178/authorguide#statisticalanalysis

If n<5, please show single datapoints for diagrams.

9) Please also note our reference format:
http://www.embopress.org/page/journal/14693178/authorguide#referencesformat

10) We updated our journal's competing interests policy in January 2022 and request authors to consider both actual and perceived competing interests. Please review the policy https://www.embopress.org/competing-interests and update your competing interests if necessary. Please name this section 'Disclosure and Competing Interests Statement' and put it after the Acknowledgements section.

11) We now use CRediT to specify the contributions of each author in the journal submission system. CRediT replaces the author contribution section. Please use the free text box to provide more detailed descriptions. See also guide to authors: https://www.embopress.org/page/journal/14693178/authorguide#authorshipguidelines

12) Please order the manuscript sections like this, using these names:
Title page - Abstract - Keywords - Introduction - Results - Discussion - Materials and Methods - Data availability section - Acknowledgements (including funding information) - Disclosure and Competing Interests Statement - References - Figure legends - Expanded View Figure legends

I look forward to seeing a revised version of your manuscript when it is ready. Please let me know if you have questions or comments regarding the revision.

Yours sincerely,

Achim Breiling
Senior Editor
EMBO Reports

Referee #1:

The manuscript "CCR4-NOT differentially controls host vs. virus poly(A)-tail length and regulates HCMV infection" by Burgess et al. reports HCMV-induced CCR4-NOT complex is required for HCMV lytic replication. Authors revealed that CCR4-NOT complex regulates global transcriptome in infected cells by RNA-seq and nanopore DRS. It is interesting that CNOT1 and CNOT3, the subunits of CCR4-NOT complex, are novel regulators during HCMV life cycle. However, the mechanism of how these proteins control gene expression landscape remains unclear and overall data is premature to support it. Some critical issues should be addressed for publication.

Major comments:

1. Authors are encouraged to investigate whether both of the two catalytic subunits of CCR4-NOT complex, CCR4 and CAF1, are required for HCMV infection: In Figure 6, the data for CCR as well as CAF1 need to be included. Authors mentioned that CCR4 (CNOT6/6L) and CAF1 (CNOT7/8) were not identified as candidates in RNAi screens due to the low knockdown efficiency (line 319). However, this is an inappropriate explanation because each protein was sufficiently depleted by siRNA (Figure S1). Also, the explanation that CCR4 and Caf1 have redundancy with each other is also inappropriate (line 317). This contradicts their own argument that inhibition of CAF1 alone by drug could inhibit HCMV infection.

2. The authors argued that CNOT1 and CNOT3 were important for HCMV infection. However, the authors rarely present data on both CNOT1 and CNOT3 and only show data on CNOT1 (figure 2, S2 and 7).

3. It is interesting that CCR4-NOT complex proteins including CNOT1 and CNOT3 are upregulated in HCMV infected cells (Figure 4a). Does HCMV actively upregulate the expression of the CNOT complex? Can CNOT subunits be upregulated by other stimuli such as interferon treatment? Experiment could be performed using UV-inactivated HCMV or neutralizing antibodies against IFN-receptor.

4. In RNA-seq and DRS data, the relationship between differential gene expression and deadenylase activity of CCR4-NOT is still elusive. Because CCR4-NOT complex globally regulates RNA homeostasis, it is not surprising that global host mRNA level is changed by CNOT1 depletion. Therefore, it is critical to find a direct target whose poly(A) length is regulated by CNOT1. Although overall poly(A) length of host mRNA is increased in CNOT1-depleted cells (Figure 7c), there is no correlation between poly(A) length and expression changes (Figure S3). What is the implication of this result? Authors mentioned that only SPARC and ANXA shows increased gene expression and poly(A) tail in CNOT1-depleted cells. Then, examine poly(A) length or stabilities of these two transcripts and whether they are essential for HCMV lytic replication.

5. Authors claim that CNOT1 selectively shortens poly(A) of host mRNAs but not that of viral mRNAs. However, the poly(A) length distribution of total mRNA presented in the Figure 7c alone is not sufficient to support this hypothesis. Present the poly(A) length distribution of multiple representative host and viral mRNAs in DRS data. Moreover, authors should experimentally validate the stabilities or poly(A) length of these transcripts.

Minor comments:

1. In line 148-158, The authors have a slightly exaggerated interpretation of siRNA screening data The effect of these proteins on viral infection seems minimal, and the effect seems to be different depending on the siRNA sequence (Figure 1b). Describe only statistically significant proteins, or focus on CNOT1 and CNOT3.

2. In Figure 2, authors need to check whether virus entry was not affected by CNOT1 or CNOT3 knockdown at immediate early time point.

3. In line 195-197, authors mentioned "viral DNA synthesis is dependent on CCR4-NOT function", but this sentence may confuse readers that CCR4-NOT directly participate in viral DNA replication

4. In Figure 4b, the authors can delete data on TNKS1BP1. Its expression was not increased like other complex proteins and the authors also did not analyze the physiological relevance of TNKS1BP1 in HCMV infection.

5. Table S3 is missing. Line 383 : Table S3 might be corrected to Table S4.

Referee #2:

In this manuscript, Burgges et al., dissect the role of the protein complex CCR4-NOT during HCMV infection, and found a major role for the proteins CNOT-1 and CNOT-3 as HCMV dependency factors. Interestingly, the importance of CNOT1/3 was unique to HCMV and CNOT1/3 KD did not affect HSV-1 or VACV propagation. While CNOT1/3 mainly affects host transcripts and shortens its poly-A tail, it had only a small effect on the viral transcript, indicating HCMV depends on CCR4-NOT complex through effects on cellular mRNA deadenylation.

Those findings shed light on a new potential mechanism in which HCMV specifically reduce cellular mRNA stabilization and by that, potentially increase viral protein production.

The paper is clearly written and the work constitutes a solid ground for further research on how CCR4-NOT selectively deadenylates host transcripts over viral transcripts.

Main concerns:
- Although CCR4-NOT complex was shown to have a major contribution to HCMV replication, the mechanism remains vague and whether the effect is indeed mediated by deadenylation of cellular genes is not fully established. Given the effect on infection seems to be early and the siRNA treatment was done prior to infection it will be important to verify there is no major changes in the initial entry of CMV (one possible explanation to the specificity to HCMV is that pDGFRa or other critical entry components are downregulated by CCR4-NOT KD).

Minor concerns:
1. In the RNA-seq (Fig. 5d) it is hard to tell if the effect on viral genes is indeed global. Separating the transcripts into the temporal classes will help to demonstrate that the effect is indeed general.
2. Statistic in Fig. 4b is missing.
3. Line 278 should refer to Fig. 5e (not 5c)

Referee #3:

In contrast to other herpesviruses, HCMV lacks a virus-encoded RNA decay factor and thus manipulates host mRNA decay proteins. This study describes that several members of the host CCR4-NOT deadenylase complex are essential for efficient HCMV reproduction. The authors provide some additional evidence showing that HCMV transcripts have markedly longer poly(A)-tails to cellular mRNAs and as such are less sensitive to CCR4-NOT disruption, although the functional significance seems to be limited to a few RNAs.

A key concern is that the data implying deadenylase activity is impacted through CNOT1 depletion is a strong conclusion to draw from general changes in abundance/extended poly(A)-tails. The link between CNOT1/CNOT3, the CCR4-NOT complex and its deadenylation activity affecting HCMV is not shown experimentally. Increased deadenylation of host mRNA transcripts resulting in increased stability/ribosomal access also requires experimental validation to draw such conclusions.

Overall the conclusions are overly strong compared to the experimental evidence, with little linking CNOT1/3 KD to changes in tail length and its implications for gene expression.

Specific points:
1. Figure 2a/b - levels of RNA and protein do not seem to correlate for UL44 and UL99. Why only a 50% reduction in RNA levels but a dramatic effect on protein levels. Would be useful to show RNA levels in timecourse and how they correspond to protein.
2. Fig S2. Figure S2. Why do siRNA for CNOT1 and CNOT3 result in respective upregulation of each others RNA. Significant values? Significant upregulation of CNO1 RNA by siRNA CNO3 - CNOT1 RNA increases when CNOT3 kd, CNOT3 RNA increases when CNOT1 kd however this is not reflected in the westerns - as CNOT1 protein is reduced with both CNOT1 and CNOT3 kd.
3. Fig 3. Lines 224 seems rather over-interpreted saying it is HCMV selective, based on screen with only two other viruses. Is there an explanation for the accumulation of gC in HSV-1 CNOT3 KD?
4. Fig 4. Although selective regulation of certain CCR4-NOT complex was shown, it adds little to the understanding of how CRR4-NOT complex functions and is regulated in HCMV infection. Why is there a reduction of CNOT3 protein at 72 hours in AD169 and upregulation in TB40/E, neither of which is reflected in the RNA levels?
5. Fig. 5. As stated in the text lines 263-267. Further work is required to explain the observation targeting of mRNA decay machinery results in a similar proportion of positive and negative gene
expression changes. What are the secondary indirect effects of dysregulated host or viral genes which influence host gene expression in CNOT1/3 knockdown cells.
6. Fig 5d. is the reduction in viral reads upon CNO1/CNOT3-depletion evenly distributed across genome as stated on line 271. There only seems certain parts of the genome from Fig 5d.

7. Fig 5e. Functional significance of the cellular pathways associated with the set of genes differentially expressed by either CNOT1 or CNOT3 depletion seems a little vague - eg lines 293-295 and lines 312-33. Further evidence is required to support significance.
8. Fig 7. Provides interesting results that CNOT depletion affected the overall tail length of cellular mRNAs in contrast to viral mRNAs. However no overall correlation in expression levels, apart from 2 mRNAs, was observed which lessens the impact of this finding. For dysregulated genes in the KDs, how much can be attributed to the KD directly and how much is linked to the reduction in HCMV replication observed? Stability changes need experimental validation of SPARC and ANXA increased poly(A)-tail length.

Additional comments:
Line 122: DAPI counts unusual way to measure cell viability, more quantitative methods available.
Line 137 - Why mention the "modest but consistent" reduction in CNOT6L, where other components such as XRN1 may trend lower. Also implies significance, which is not the case.
Line 180 - Upregulation of CNOT1/3 appears strong. At what time point does this occur? Does it occur at protein + RNA?
Line 189 - What is the effect of CNOT3 depleted cells? Why only CNOT1 western of 0-72 hpi?
Line 218 - Why are IE1/2 largely effected in TB40IE sig3b but not S2c?
Line 222- Text refers to AD169 strain, but figure is labelled TB40/E.
Line 237 - CNOT3 upregulation is much slower in TB40 than 169 but opposite for CNOT1. Different functions?
Line 260 - Many do not overlap - suggesting different roles for CNOT1 and CNOT3, correlation analysis only on overlapping gene.
Line 349 - if stability is affected by longer tails - actinomycin D assay +/-s KDs of viral genes can confirm this.

RESPONSE TO REVIEWERS - EMBOR-2022-56327V2

We thank the reviewers for their thoughtful comments on our manuscript. A revised version that includes new data has been uploaded where text revisions are indicated in red font.

Note our supplementary figures and tables are now renamed as Extended View (EV) figures, tables and datasets per EMBO reports guidelines for revised manuscripts.

Our point-by-point response (red text) to the reviewer's comments follows:

Referee #1:

The manuscript "CCR4-NOT differentially controls host vs. virus poly(A)-tail length and regulates HCMV infection" by Burgess et al. reports HCMV-induced CCR4-NOT complex is required for HCMV lytic replication. Authors revealed that CCR4-NOT complex regulates global transcriptome in infected cells by RNA-seq and nanopore DRS. It is interesting that CNOT1 and CNOT3, the subunits of CCR4-NOT complex, are novel regulators during HCMV life cycle. However, the mechanism of how these proteins control gene expression landscape remains unclear and overall data is premature to support it. Some critical issues should be addressed for publication.

Major comments:

1. Authors are encouraged to investigate whether both of the two catalytic subunits of CCR4-NOT complex, CCR4 and CAF1, are required for HCMV infection: In Figure 6, the data for CCR as well as CAF1 need to be included.

Authors mentioned that CCR4 (CNOT6/6L) and CAF1 (CNOT7/8) were not identified as candidates in RNAi screens due to the low knockdown efficiency (line 319). However, this is an inappropriate explanation because each protein was sufficiently depleted by siRNA (Figure S1).

Also, the explanation that CCR4 and Caf1 have redundancy with each other is also inappropriate (line 317). This contradicts their own argument that inhibition of CAF1 alone by drug could inhibit HCMV infection.

We thank the reviewer for highlighting the lack of clarity in our description of the nucleases as redundant and have taken the opportunity to amend the text interpreting our results for Fig1b and Fig 6 and supply an additional experiment addressing this point. We now highlight that, as this reviewer notes, the targets of our siRNAs were each significantly depleted (Fig EV1a) but that even residual levels of nucleases may be sufficient to supply CCR4-NOT with deadenylase activity. Of note, while is unknown to what excess the CCR4 and CAF1 enzymatic nucleases are present in the cell compared to CNOT1 scaffold proteins, it was recently shown that depletion of CNOT7 stabilizes CNOT8 protein and increases its incorporation into CCR4-NOT (PMID: 35248544). We now include data showing that even co-depletion of CNOT6/6L/7/8 together does not interfere with a low multiplicity HCMV infection (Fig. EV1d, shown below).

We wholeheartedly agree with this reviewer however that establishing whether both nucleases are essential for HCMV is an important question and that contrasting our CAF1 (CNOT7/8) inhibitor results with a CCR4 (CNOT6/6L) inhibitor would be very valuable. There is, however, to our knowledge no drug available that inhibits both paralogs of this nuclease module. A recent publication describing a CNOT6L selective inhibitor did not disclose the compound's identity (PMID: 35385705), and neither did the authors when asked.

EV1d:

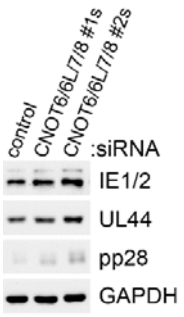

2. The authors argued that CNOT1 and CNOT3 were important for HCMV infection. However, the authors rarely present data on both CNOT1 and CNOT3 and only show data on CNOT1 (figure 2, S2 and 7).

We thank the reviewer for their interest in the specific role of CNOT3 in HCMV infection. We do show in Figure 2 the impact of both CNOT1 and CNOT3 knockdown on viral protein (Fig 2a) and RNA (fig 2b) accumulation, each showing a similar effect. Figure 3 also shows the effect of both CNOT1 and CNOT3 depletions on viral protein production during infection with the HCMV clinical strain TB40E and other viruses (VACV, HSV-1). Figure S2D (now renamed EV2f) shows the effect of both CNOT1 and CNOT3 knockdowns on clinical strain viral reproduction. However, in response to both this reviewer's and reviewer 3's interest, we have now further explored CNOT3's role specifically by including a time course of infection in CNOT3-depleted cells vs control cells (Fig EV 2d; and below), to allow the comparison with CNOT1 knockdown data (Fig 2C). New data exploring the potential for CCR4-NOT disruption to influence viral entry (Fig EV2c and below) also includes both CNOT1 and CNOT3 depletion.

Figs EV2c,d:

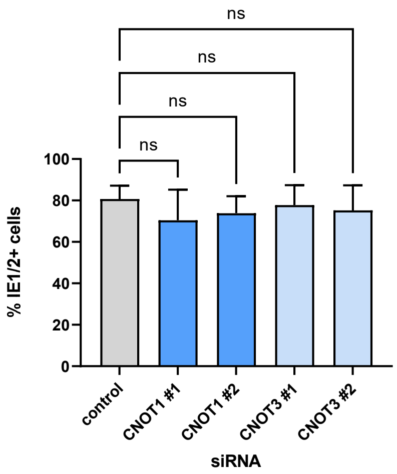

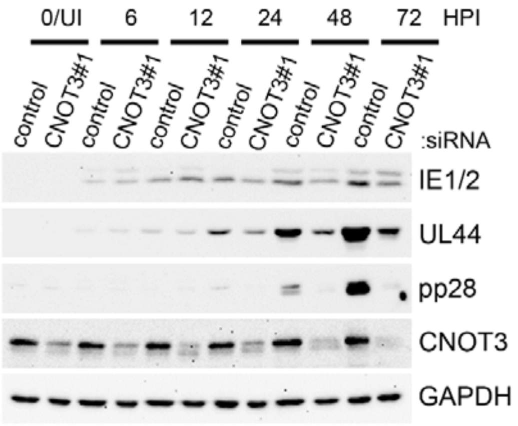

3. It is interesting that CCR4-NOT complex proteins including CNOT1 and CNOT3 are upregulated in HCMV infected cells (Figure 4a). Does HCMV actively upregulate the expression of the CNOT complex? Can CNOT subunits be upregulated by other stimuli such as interferon treatment? Experiment could be performed using UV-inactivated HCMV or neutralizing antibodies against IFN-receptor.

We agree that virus-induced changes to CNOT-expression are interesting and suggestive of viral manipulation of this complex upon which it is dependent for replication. How HCMV achieves this upregulation will be a focus of our future work. Fasting/feeding has also been reported to influence CNOT3 protein levels in select mouse tissues through an undefined translational control mechanism (PMID: 21897366) and we have now cited this work (p9) to make clear that the notion of regulation of CCR4-NOT complex components in response to physiological stimuli has precedence.

4. In RNA-seq and DRS data, the relationship between differential gene expression and deadenylase activity of CCR4-NOT is still elusive. Because CCR4-NOT complex globally regulates RNA homeostasis, it is not surprising that global host mRNA level is changed by CNOT1 depletion. Therefore, it is critical to find a direct target whose poly(A) length is regulated by CNOT1. Although overall poly(A) length of host mRNA is increased in CNOT1-depleted cells (Figure 7c), there is no correlation between poly(A) length and expression changes (Figure S3). What is the implication of this result? Authors mentioned that only SPARC and ANXA shows increased gene expression and poly(A) tail in CNOT1-depleted cells. Then, examine poly(A) length or stabilities of these two transcripts and whether they are essential for HCMV lytic replication.

We have now further experimentally investigated the link between overall mRNA abundance changes detected by RNAseq and RNA stability changes induced by CNOT protein knockdown, linking CCR4-NOT function to the dysregulation of host genes. Our new data figure EV4b (replacing Fig 5f) examines 15 of the 25 host mRNAs strongly and significantly upregulated in infected CNOT1 depleted cells by RT-qPCR, validating their upregulation in this condition and showing they are also increased in uninfected knockdown cells. This shows a direct role for CNOT1 in their regulation, rather than this being a consequence of altered viral gene expression. Further, our additional new data figure (Fig 5f, and below) shows that mRNAs upregulated in CNOT1/3 kd cells also experience increased stability, explaining their abundance changes.

The impact of an extended poly(A)-tail and concomitant extended half-life on the overall abundance of an mRNA will be affected by the size of the existing pool of that mRNA and any transcriptional changes that occur. ie a stabilization of a low abundance message may change its total levels by several fold where a lesser impact may be observed for an mRNA of high abundance. A limitation of DRS is its low sampling depth, biasing our poly(A)-tail dataset to high abundance messages for which stability changes are less likely to produce an impact on their total pool. It is therefore unsurprising that mRNA abundances (RNA-Seq) do not directly correlate with poly(A)-tail length changes measured by DRS (Fig EV5b). Moreover, though we were able to measure the poly(A) tails on only a small subset of mRNAs upregulated upon CNOT1-knockdown (SPARC, ANXA1), this does not imply that other upregulated mRNAs do not also have poly(A)-tail length changes. We have now revised our results section text to better explain this (p15).

As requested, we specifically investigated the stability of SPARC and ANXA1 mRNAs and found ANXA1 to be stabilized by CNOT1/3 depletion. SPARC mRNA was extremely stable ($t_{1/2}$ > 8h), obviating testing the effect of CNOT1/3 knockdown on its half-life. We also tested whether these genes are essential for HCMV lytic replication and found that knockdown of these two, or LRRC15, the upregulated host gene with the highest predicted antiviral activity (Hein & Weissman 2021, PMID: 23180859) did not measurably affect viral replication (new Fig 5g, EV4c).

Together our new data show that CCR4-NOT disruption leads to the dysregulation of many host genes directly through changes to their transcript stability, but the contribution of host factor dysregulation to the CNOT1/3 phenotype is likely to be multigenic.

Fig 5f

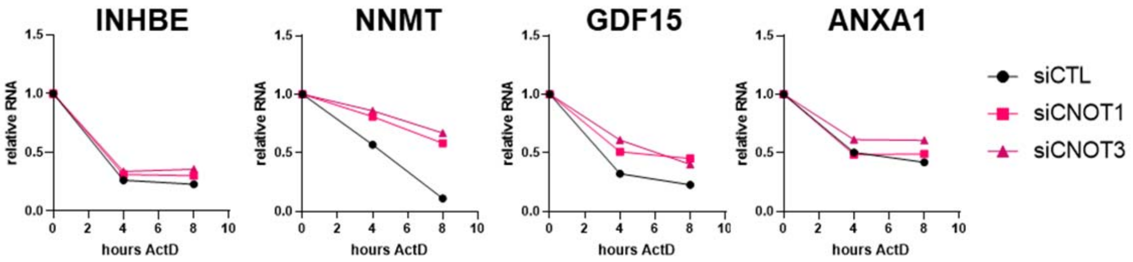

Fig 5g:

EV4C:

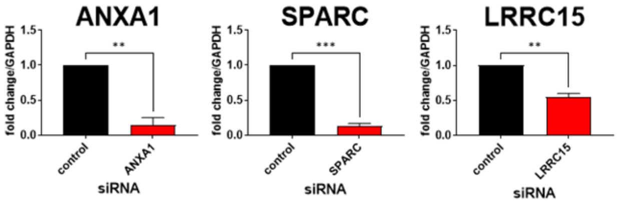

5. Authors claim that CNOT1 selectively shortens poly(A) of host mRNAs but not that of viral mRNAs. However, the poly(A) length distribution of total mRNA presented in the Figure 7c alone is not sufficient to support this hypothesis. Present the poly(A) length distribution of multiple representative host and viral mRNAs in DRS data. Moreover, authors should experimentally validate the stabilities or poly(A) length of these transcripts.

We have addressed this point with two new figures. In new figure 7d we now show the effect on the median tail length of all measured host and viral poly(A) tails, allowing comparison of multiple host and viral mRNAs (p14). This figure now better visually shows the effect on each population of mRNAs, and supports our conclusion that CNOT1 has a greater impact on host compared to viral poly(A)-tails. Note, we do not claim that viral poly(A)-tails are not shortened by CCR4-NOT.

As described in response to point 4, we have now experimentally examined the stability of several host transcripts to determine the effect of CNOT1/3 depletion, finding evidence of CCR4-NOT-dependent stabilization. We were unable to optimize standard Actinomycin D transcriptional inhibition assay to measure mRNA decay in infected cells and maintain cell survival/attachment. Our goal for future work is to interrogate mRNA decay rates globally in infected cells using metabolic sequencing (e.g. SLAM-seq).

Fig 7d:

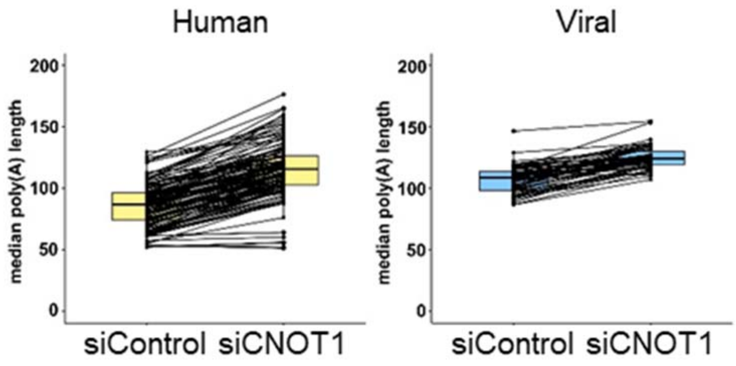

Minor comments:

1. In line 148-158, The authors have a slightly exaggerated interpretation of siRNA screening data The effect of these proteins on viral infection seems minimal, and the effect seems to be different depending on the siRNA sequence (Figure 1b). Describe only statistically significant proteins, or focus on CNOT1 and CNOT3.

Though we believed we made clear where effects that did not reach statistical significance were described we have taken the recommendation of this reviewer and removed this, focusing only on CNOT proteins.

2. In Figure 2, authors need to check whether virus entry was not affected by CNOT1 or CNOT3 knockdown at immediate early time point.

This was also a concern for Reviewer 2. Equivalent expression of IE1/2 at 6HPI in the CNOT1-knockdown time course indicated that virus entry was not affected, however, we have now added an immunofluorescence experiment that additionally finds a similar % of IE1/2-positive cells in control, siCNOT and siCNOT3 cells (new Fig. EV2c, p7). Equivalent IE1/2 protein expression is also observed by western blot in CNOT3-depleted cells in the new Fig. EV2d. At the suggestion of Reviewer 2 we also checked expression of known HCMV entry receptor PDGFRA and do not detect its downregulation in our RNA-seq data, indeed it is slightly upregulated in CNOT1/3 knockdown cells (Dataset EV1). We believe that together these data indicate that CNOT1/3 knockdowns do not affect HCMV entry.

3. In line 195-197, authors mentioned "viral DNA synthesis is dependent on CCR4-NOT function", but this sentence may confuse readers that CCR4-NOT directly participate in viral DNA replication

We agree and have amended and clarified this statement as requested. It now reads, “Indeed, viral DNA levels were reduced by more than 80% by each CNOT1-specific siRNA (Fig. 2d). This is consistent with the notion that CCR4-NOT function is required for viral DNA synthesis to be successfully completed, possibly by impacting expression of virus or host genes needed for DNA replication“. (p8)

4. In Figure 4b, the authors can delete data on TNKS1BP1. Its expression was not increased like other complex proteins and the authors also did not analyze the physiological relevance of TNKS1BP1 in HCMV infection.

We have now removed this data.

5. Table S3 is missing. Line 383 : Table S3 might be corrected to Table S4.

Table S3 (Genes strongly upregulated (>2 fold) in both CNOT1- or CNOT3-depleted HCMV-infected cells relative to control -infected cells) was uploaded as supplemental data excel file in our initial submission and is included for this resubmission as Table EV2 in accordance with EMBO reports conventions.

Referee #2:

In this manuscript, Burgges et al., dissect the role of the protein complex CCR4-NOT during HCMV infection, and found a major role for the proteins CNOT-1 and CNOT-3 as HCMV dependency factors. Interestingly, the importance of CNOT1/3 was unique to HCMV and CNOT1/3 KD did not affect HSV-1 or VACV propagation. While CNOT1/3 mainly affects host transcripts and shortens its poly-A tail, it had only a small effect on the viral transcript, indicating HCMV depends on CCR4-NOT complex through effects on cellular mRNA deadenylation.

Those findings shed light on a new potential mechanism in which HCMV specifically reduce cellular mRNA stabilization and by that, potentially increase viral protein production.

The paper is clearly written and the work constitutes a solid ground for further research on how CCR4-NOT selectively deadenylates host transcripts over viral transcripts.

Main concerns:
- Although CCR4-NOT complex was shown to have a major contribution to HCMV replication, the mechanism remains vague and whether the effect is indeed mediated by deadenylation of cellular genes is not fully established.

We are grateful this reviewer recognizes we have identified a host RNA decay complex with a major role in HCMV replication. In our revised manuscript we further investigate the mechanism by which CCR4-NOT regulates host gene expression, and the contribution of this to HCMV replication. We have now directly assessed the stability of mRNAs dysregulated by CCR4-NOT disruption (new Fig 5f) and their regulation in uninfected cells (new EV4b), finding evidence for their CCR4-NOT-dependent stabilization, independent from changes to viral gene expression. We investigated the interleukins implicated by pathway analysis of siCNOT1/3-differentially expressed genes (DEGs) and found these did not significantly impact HCMV replication in primary fibroblasts (EV4a). We also tested the ability of the DEGs most strongly implicated in a genome-wide host factor screen (Hein & Weissman 2021, PMID: 23180859) to regulate HCMV and individually explain the CNOT1/3-phenotype and found each was unable to do so (new Fig 5g). Together our new data show that CCR4-NOT disruption leads to the dysregulation of many host genes directly through changes to their transcript stability, but the contribution of host factor dysregulation to the CNOT1/3 phenotype is likely to be multigenic and potentially multi-factorial.

The possibility remains open, and exciting, that global alterations to the poly(A)-tail (i.e. PABPC1 substrate) pool alter HCMV mRNAs' ability to recruit PABP and effectively compete for ribosome recruitment, as explained in our Discussion (p16/17). Distinguishing between the importance of transcriptome changes to impact production of many host-encoded

HCMV-regulatory factors versus dysregulation of the availability of RNA-binding proteins (such as, but not limited to, PABPC1) for HCMV mRNA stability and translation regulation will be complex, and beyond the scope of our present study.

-Given the effect on infection seems to be early and the siRNA treatment was done prior to infection it will be important to verify there is no major changes in the initial entry of CMV (one possible explanation to the specificity to HCMV is that pDGFRa or other critical entry components are downregulated by CCR4-NOT KD).

In response to this concern and that of reviewer 1 we have now performed additional experiments to ascertain if there is an effect on viral entry in CNOT1 or CNOT3 knockdown cells. Our original timecourse experiment demonstrated equivalent expression of IE1/2 proteins at 6HPI in the CNOT1-depleted cells, indicating that virus entry is not affected, and we have now made this conclusion clear in the accompanying text (p7). Equivalent IE1/2 protein expression is also observed by western blot in CNOT3-depleted cells in the new Fig. EV2d, shown below. A new immunofluorescence experiment additionally finds a similar % of IE1/2-positive cells in control, siCNOT and siCNOT3 cells (new Fig. EV2c, shown below). Though PDGFRA was among the host genes differentially expressed in CNOT1- and CNOT3 knockdown cells (Dataset EV1), it was upregulated 0.62 log2fold (ie 1.5 fold) and 0.87 log2fold (ie 1.8 fold) in these respective conditions. Together these data argue against CNOT1 and 3-depleted cells being intrinsically less permissive to HCMV entry.

Figs EV2c,d:

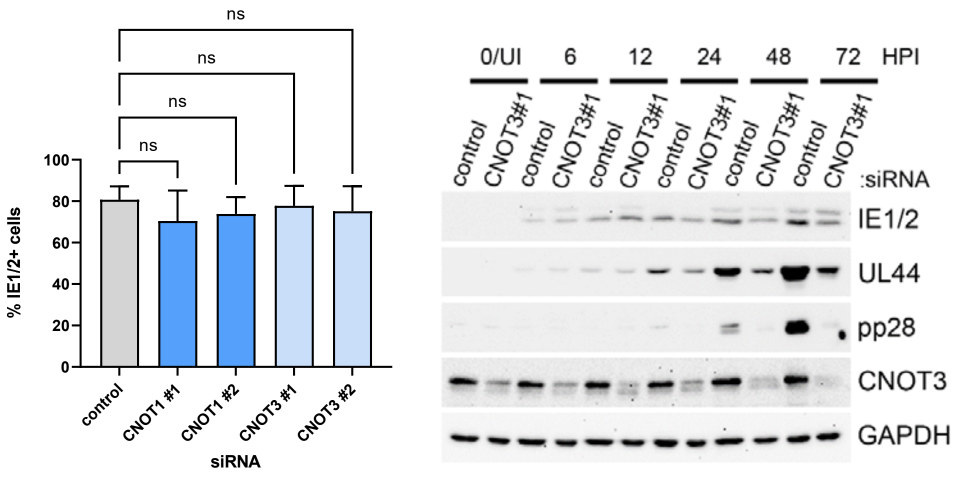

Minor concerns:

1. In the RNA-seq (Fig. 5d) it is hard to tell if the effect on viral genes is indeed global. Separating the transcripts into the temporal classes will help to demonstrate that the effect is indeed general.

The analysis of the RNAseq data in Fig 5d has been clarified and the section title revised to more accurately describe our findings (p10). In response to this reviewer’s concern that the effect in Fig 5d is hard to discern, we have added an additional figure (EV3) directly comparing the distribution of viral reads between control and CNOT1- and CNOT3-depleted cells. This more obviously shows a largely consistent pattern of distribution of reads across the genome in knockdown cells compared to control.

The short-read sequencing undertaken to fully characterize changes to the host transcriptome for this experiment was not conducted using strand-specific library preparations, precluding assignment of viral reads to individual transcripts (and thus their temporal class) in regions where multiple genes overlap on the same strand and/or are expressed from opposing viral genome strands. We can however identify specific gene loci where transcripts are detected with reduced abundance in knockdown cells, which we have provided examples of.

2. Statistic in Fig. 4b is missing.

We have now corrected this, updating the figure and legend.

3. Line 278 should refer to Fig. 5e (not 5c)

We have now corrected this and thank the reviewer for bringing these oversights to our attention.

Referee #3:

In contrast to other herpesviruses, HCMV lacks a virus-encoded RNA decay factor and thus manipulates host mRNA decay proteins. This study describes that several members of the host CCR4-NOT deadenylase complex are essential for efficient HCMV reproduction. The authors provide some additional evidence showing that HCMV transcripts have markedly longer poly(A)-tails to cellular mRNAs and as such are less sensitive to CCR4-NOT disruption, although the functional significance seems to be limited to a few RNAs.

A key concern is that the data implying deadenylase activity is impacted through CNOT1 depletion is a strong conclusion to draw from general changes in abundance/extended poly(A)-tails. The link between CNOT1/CNOT3, the CCR4-NOT complex and its deadenylation activity affecting HCMV is not shown experimentally. Increased deadenylation of host mRNA transcripts resulting in increased stability/ribosomal access also requires experimental validation to draw such conclusions.

Overall the conclusions are overly strong compared to the experimental evidence, with little linking CNOT1/3 KD to changes in tail length and its implications for gene expression.

This reviewer states "HCMV lacks a virus-encoded RNA decay factor and thus manipulates host mRNA decay proteins." as existing knowledge. Rather, we are the first to demonstrate a dependence on and manipulation of a core host mRNA decay pathway by HCMV.

The central role in cellular mRNA deadenylation and destabilization of the CCR4-NOT complex is widely established (review article PMID: 34594027 references key papers from the early 2000s). Here, we also directly demonstrate a remarkable lengthening of host poly(A)-tails using direct RNA sequencing when CNOT1 is depleted (Fig 7) and contend this is *strong evidence* for disruption of CCR4-NOT's deadenylase activity. We directly target deadenylase activity using a specific chemical inhibitor of a nuclease module of the complex (Fig 6), demonstrating an effect on viral replication by this activity of CCR4-NOT. We now show that select dysregulated host genes are stabilized by CNOT1-depletion (new Fig 5f), consistent with the widespread lengthening of poly(A) tails observed in Fig 7 and the well-established connection between poly(A) tail length and RNA stability. We now also further narrow our mechanistic investigation of the role of CNOT1 and -3 in HCMV infection by excluding host pathways and single dysregulated genes through functional interrogation of their importance for viral replication (new Fig EV4a, Fig 5d).

Specific points:
1. Figure 2a/b - levels of RNA and protein do not seem to correlate for UL44 and UL99. Why only a 50% reduction in RNA levels but a dramatic effect on protein levels. Would be useful to show RNA levels in timecourse and how they correspond to protein.

Indeed, a more drastic effect is observed on viral protein levels compared to RNA levels in CNOT1/3 knockdown cells (Fig 2a,b), data supported by RNA levels over the CNOT1-knockdown timecourse included as Fig S2b in the original submission, now renamed EV2b. This implies while there is impaired viral transcription, viral mRNAs are also failing to be translated, consistent with our model that viral mRNAs may fail to compete for ribosome access in the absence of a significant poly(A)-tail length advantage compared to host mRNAs, contributing to impaired viral protein synthesis and replication. We thank the reviewer for highlighting this omission and have revised the discussion section text to acknowledge it (p16/17).

2. Fig S2. Figure S2. Why do siRNA for CNOT1 and CNOT3 result in respective upregulation of each others RNA. Significant values? Significant upregulation of CNO1 RNA by siRNA CNO3 - CNOT1 RNA increases when CNOT3 kd, CNOT3 RNA increases when CNOT1 kd however this is not reflected in the westerns - as CNOT1 protein is reduced with both CNOT1 and CNOT3 kd.

We have now added statistical analyses to figure S2 (now named Fig EV2a, shown below), which shows significant reduction of each cognate siRNA target, and upregulation of CNOT3 in CNOT1 knockdown that reaches significance for siRNA #1. While this has not been reported elsewhere to our knowledge, it seems likely to us that a homeostatic feedback circuit mediates compensatory transcriptional upregulation between CNOT1 and CNOT3 when the other is depleted, or even as a response to impaired cytoplasmic RNA turnover (see PMID: 31656086). As to why this is not reflected at the protein level, we could speculate that without integration into a CNOT1-scaffolded CCR4-NOT complex, CNOT3 protein (and perhaps other CNOT proteins) may be more rapidly turned over. Co-regulation of CNOT7 and CNOT8 was recently shown to occur through protein stabilization (PMID: 35248544) and cooperative protein stability of components of large cellular complexes to maintain appropriate subunit stoichiometry is thought to be a widespread mechanism (see PMID: 35729235). We have now added this potential explanation and references to the text (p7).

Amended EV2a:

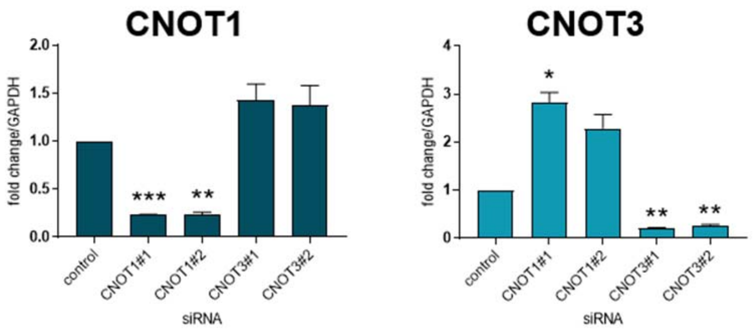

3. Fig 3. Lines 224 seems rather over-interpreted saying it is HCMV selective, based on screen with only two other viruses. Is there an explanation for the accumulation of gC in HSV-1 CNOT3 KD?

We have now edited this to state the requirement for CCR4-NOT is “not universally shared among DNA or herpes viruses” (p9). We agree that increased HSV-1 gC protein production upon CNOT3 is intriguing, raising the possibility that CNOT3/CCR4-NOT suppresses late gene expression in HSV-1 infection, and will follow this up in our future work.

4. Fig 4. Although selective regulation of certain CCR4-NOT complex was shown, it adds little to the understanding of how CRR4-NOT complex functions and is regulated in HCMV infection. Why is there a reduction of CNOT3 protein at 72 hours in AD169 and upregulation in TB40/E, neither of which is reflected in the RNA levels?

We disagree that this figure adds little to our understanding of CCR4-NOT function and regulation in infection. Figure 4 shows that the specific complex components required by the virus for efficient replication are upregulated in an infected cell. Further, it shows this is orchestrated at both the RNA and protein level, and by two different strains of the virus in which we see a CNOT1 and CNOT3-dependent phenotype. The discordance between mRNA and protein levels for CNOT1 and CNOT3 during infection timecourses is consistent with regulation at the level of translation or protein stability, as noted in the results section text of our original submission. There is precedence for the translational regulation of CNOT3 expression which we have now referenced (p9). The differences in the timing of CNOT1 and 3 protein upregulation may reflect differential viral protein expression profiles between these strains. We have now commented on the observed strain differences to CNOT1/3 regulation in the results section text and referenced a review describing gene expression differences between HCMV strains (p9).

5. Fig. 5. As stated in the text lines 263-267. Further work is required to explain the observation targeting of mRNA decay machinery results in a similar proportion of positive and negative gene expression changes. What are the secondary indirect effects of dysregulated host or viral genes which influence host gene expression in CNOT1/3 knockdown cells.

We apologise for the confusion arising from our word choice, which we have now revised, removing the term ‘secondary’. We now address the possibility of altered viral gene expression leading to host gene dysregulation by expanding our RT-qPCR analysis of differentially expressed genes (DEGs) to 15 of the 25 that were strongly and significantly upregulated in CNOT1/3-depleted infected cells (Fig. EV4b). This revealed all 15 were upregulated in uninfected CNOT1-depleted cells, implying these expression changes were not dependent on altered viral gene expression. We now also show that several of these mRNAs are stabilized by CNOT1/3-depletion, ie are directly regulated at the level of stability (Fig 5f).

6. Fig 5d. is the reduction in viral reads upon CNOT1/CNOT3-depletion evenly distributed across genome as stated on line 271. There only seems certain parts of the genome from Fig 5d.

The analysis of the RNAseq data in Fig 5d has been clarified and revised to more accurately describe our findings (p10). In response to reviewer 2’s concern that the effect in Fig 5d is hard to discern, we have now added an additional figure (EV3) directly comparing the distribution of viral reads between control and CNOT1- and CNOT3-depleted cells. This more obviously shows a largely consistent pattern of distribution of reads across the genome in knockdown cells compared to control.

7. Fig 5e. Functional significance of the cellular pathways associated with the set of genes differentially expressed by either CNOT1 or CNOT3 depletion seems a little vague - eg lines 293-295 and lines 312-313. Further evidence is required to support significance.

In our original text we expressed scepticism regarding the potential functional role of these pathways in cultured fibroblasts, we have now directly tested the ability of IL-4 and IL-12 to regulate HCMV replication in these cells, observing no significant impact (Fig. EV4a, and shown below). We have amended our text accordingly and indicated that these pathways may have a role in human infection (p11).

New Fig. EV4a:

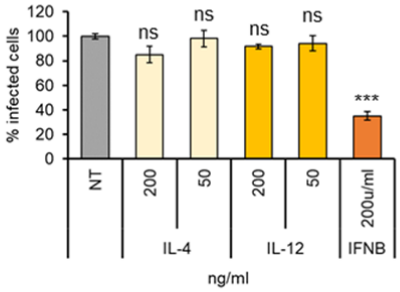

8. Fig 7. Provides interesting results that CNOT depletion affected the overall tail length of cellular mRNAs in contrast to viral mRNAs. However no overall correlation in expression levels, apart from 2 mRNAs, was observed which lessens the impact of this finding. For dysregulated genes in the KDs, how much can be attributed to the KD directly and how much is linked to the reduction in HCMV replication observed? Stability changes need experimental validation of SPARC and ANXA increased poly(A)-tail length.

Though powerful to demonstrate the global effect on cellular mRNA poly(a) tails of CNOT1 depletion, a limitation of DRS approaches on the ONT MinION is its capture of only high abundance messages (> 50 reads) with only 178-439 unique host transcripts detected in our HCMV-infected control siRNA runs. Conversely, mRNA stability changes are more likely have a greater proportionate impact on the overall pool of low abundance mRNAs though can also be obscured by transcriptional changes. It is therefore unsurprising that overall abundance of mRNAs does not directly correlate with measured poly(A)-tail lengths (Fig EV5b). We have now revised our results section text to better explain this (p15).

It is an interesting point that mRNA dysregulation we observe in CNOT1 and -3 knockdown cells could be directly due to knockdowns or secondary to reduced viral gene expression or indeed directly dysregulated host genes. To investigate this, as described above (point 5), we broadened our RT-qPCR assessment of CNOT1/3 strongly differentially expressed genes (DEGs) identified by RNA-Seq finding that all tested mRNAs (15/25) were also upregulated in uninfected CNOT1-depleted cells (now Fig. EV4b), implying these expression changes were not dependent on altered viral gene expression. As requested, we also directly examined the stability of several dysregulated host genes and found evidence of stabilization upon CNOT1- and/or CNOT3 -depletion of INHBE, NNMT, GDF15 and ANXA1 (new fig 5f). SPARC mRNA was extremely stable ($t_{1/2}$ > 8h), obviating testing the effect of CNOT1 knockdown on its half-life.

Overall, we now directly link disruption of the CCR4-NOT cellular deadenylase complex to changes in host mRNA poy(A)-tail length, stability, and overall abundance in uninfected and infected cells.

Additional comments:
Line 122: DAPI counts unusual way to measure cell viability, more quantitative methods available.

Quantifying cells with intact nuclei that conform to size thresholds permits measurements in parallel with and in an identical experimental set up to our infection assay. As cell viability is a measure of living cell numbers in a culture (in contrast to dead cell % measured by cytotoxicity assays), at 3 days post siRNA-transfection this measure will reflect any impairments to cell proliferation or cell death in the knockdown cultures that could affect our measurements of viral replication.

Line 137 - Why mention the "modest but consistent" reduction in CNOT6L, where other components such as XRN1 may trend lower. Also implies significance, which is not the case.

In response to this reviewer and Reviewer 1’s concern we have removed this statement.

Line 180 - Upregulation of CNOT1/3 appears strong. At what time point does this occur? Does it occur at protein + RNA?

We agree the upregulation of CNOT1 and CNOT3 in infection is intriguing and the timing of this is explored fully in Fig. 4a and b. Though the mRNA is upregulated for both genes, the protein is upregulated to a far greater extent hinting at a translational upregulation or protein stabilization, as described above (point 4). We plan to pursue the mechanism of HCMV-induced CNOT upregulation in our future work.

Line 189 - What is the effect of CNOT3 depleted cells? Why only CNOT1 western of 0-72 hpi?

In response to both this reviewer’s and reviewer 1’s comment we have now further explored CNOT3’s role specifically by including a time course of infection in CNOT3-depleted cells vs control cells (Fig EV 2d; and below), to allow the comparison with CNOT1 knockdown data (Fig 2C). We find a similar timing of reduced viral protein accumulation upon CNOT3 knockdown, consistent with the similar effects on viral RNA accumulation (Fig 2b). Likewise, similar to CNOT1 depletion we observe IE protein production (new Fig EV 2d) and the proportion of cells positively staining for IE1/2 expression at 6HPI (new Fig EV 2c) are not significantly affected by CNOT3 depletion.

EV 2d:

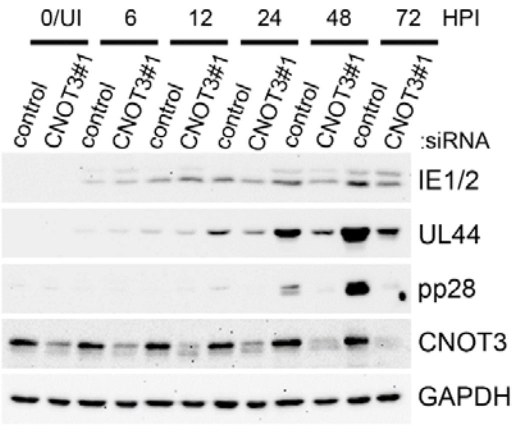

Line 218 - Why are IE1/2 largely effected in TB40/E sig3b but not S2c?

In both figures IE1/2 levels are reduced by CNOT1 knockdown in TB40/E infection at 72hpi, however a longer exposure of the time course blot in fig S2c (now EV2e) so that IE1/2 levels early in infection were detected, revealed the residual IE1/2 that is produced in siCNOT1 cells at 72hpi.

Line 222- Text refers to AD169 strain, but figure is labelled TB40/E.

This text compares the effect of CNOT1 knockdown in TB40/E infection (Fig. S2c, now EV2e) with that of AD169 infection (Fig 2c). We have now rearranged the figure reference in the sentence to make this clear (p8).

Line 237 - CNOT3 upregulation is much slower in TB40 than 169 but opposite for CNOT1. Different functions?

As described above (point 4), the differences in the timing of CNOT1 and 3 protein upregulation may reflect differential viral protein expression profiles between these strains. The AD169 lab strain harbours a 15kb deletion and multiple other mutations of genes present in clinical strains such as TB40/E. We have now commented on the observed strain differences to CNOT1/3 regulation in the results section text and referenced a review describing these gene expression differences (p9).

Line 260 - Many do not overlap - suggesting different roles for CNOT1 and CNOT3, correlation analysis only on overlapping gene.

We have now acknowledged this in the text (p10).

Line 349 - if stability is affected by longer tails - actinomycin D assay +/-s KDs of viral genes can confirm this.

We were able to characterize the effect of CNOT1/3-depletion on cellular mRNA decay using this assay (new Fig 5f), but unfortunately were unable to optimize successfully applying prolonged actinomycin treatment to infected cells without compromising cell survival/attachment to examine viral mRNA decay within the revision deadline. We are exploring metabolic labelling + RNAseq approaches to globally interrogate viral RNA decay dependence on CCR4-NOT which we hope to apply in our future work.

Dear Dr. Burgess,

Thank you for the submission of your revised manuscript to our editorial offices. I have now received the reports from three referees that I asked to re-evaluate your study, you will find below. As you will see, the referees are mostly satisfied by the revision and support publication of the manuscript in EMBO reports. Nevertheless, referees #1 and #3 have remaining points I ask you to address in a final revised manuscript. I think to include a schematic diagram that elucidates the operational mechanism of CNOT1 and to extent the discussion part would improve the manuscript

Moreover, I have these editorial requests I also ask you to address:

- We need individual production quality figure files as .eps, .tif, .jpg (one file per figure), of main and EV figures. Please upload these as separate, individual files upon re-submission.

- Regarding data quantification and statistics, please check again all panels make sure that the number "n" for how many independent experiments were performed, their nature (biological versus technical replicates), the bars and error bars (e.g. SEM, SD) and the test used to calculate p-values is indicated in the respective figure legends (main and EV figures). Please also check that all the p-values are explained in the legend, and that these fit to those shown in the figure. Please provide statistical testing where applicable. Please avoid the phrase 'independent experiment', but clearly state if these were biological or technical replicates. Please also indicate (e.g. with n.s.) if testing was performed, but the differences are not significant. In case n=2 please show the data as separate datapoints or bars without error bars and statistics. See also:
http://www.embopress.org/page/journal/14693178/authorguide#statisticalanalysis

If n<5, please show single datapoints for diagrams. It seem the above information is (partly) missing for 4B, 4G, 6B and EV1A. Please check.

- Please make sure that Datasets and Tables are called out correctly. Presently, e.g. Dataset EV1 is called out as 'Dataset S1'. Please check.

- Dataset EV2 is missing a legend in the excel file. Dataset EV4 needs correction of the name and the legend in the excel file. Please check.

- Please remove the 'Author Summary' from the manuscript text file.

- Please provide the fully completed source data checklist and provide the final source data as discussed with Hannah Sonntag. Please make sure all the requested source data is provided.

- Finally, during our standard image analysis, we detected potential aberrations in the figure set, and we would like to clarify the issue. We kindly invite you to check the composition of Figure EV1D - IE1/2 - in between the first blot and the second blot (see the attached screenshot). Was the image spliced? If yes, please indicate this in the figure with a black line. If not, could you please send us the related source data?

In addition, I would need from you:
- a short, two-sentence summary of the manuscript (not more than 35 words).
- two to four short (!) bullet points highlighting the key findings of your study (not more than two lines each, 1 sentence).
- a schematic summary figure as separate file that provides a sketch of the major findings (not a data image) in jpeg or tiff format (with the exact width of 550 pixels and a height of not more than 400 pixels) that can be used as a visual synopsis on our website.

I look forward to seeing the final revised version of your manuscript when it is ready. Please let me know if you have questions regarding the revision.

Best,

Achim Breiling
Senior Editor
EMBO Reports

Referee #1:

This paper introduces a novel aspect of HCMV's behavior by highlighting its utilization of the host mRNA decay pathway. Although the revelation of this mechanism is significant, its correlation with the established functions of the CCR4-NOT Deadenylase complex somewhat tempers its level of surprise, given the complex's recognized involvement in various cellular metabolic processes. In response to my comments, the authors have diligently incorporated revisions, including additional experimental work and textual enhancements. However, despite these efforts, the manuscript still lacks a comprehensive elucidation of the functional targets of CNOT1 and their biological relevance. This critical point, emphasized not only by myself but also by Reviewer 3, remains insufficiently addressed.

Unfortunately, the functional validation experiment pertaining to ANXA1, SPARC, and LRRC15, which were the targets initially pinpointed by the authors (as illustrated in Fig 5g), did not yield definitive positive results. This outcome is rather disappointing.

The paper's composition displays a level of complexity that at times hinders its readability and ease of understanding. In order to make the content more accessible, I recommend incorporating a schematic diagram that elucidates the operational mechanism of CNOT1. This visual aid would significantly enhance the overall reader-friendliness of the material.

Referee #2:

The authors have addressed my concerns

Referee #3:

Key concerns from all 3 reviewers were the lack of a potential mechanism of how the CCR4-NOT complex regulated gene expression during HCMV infection. Although overall poly(A) length of host mRNA was increased in CNOT1-depleted cells, no correlation between poly(A) length and expression changes were shown, questioning the implication of this result.

The manuscript has been enhanced by additional data specifically :
(i) Figure EV1d which now clearly shows the mechanism is specific to CNOT1 and 3 and CCNO6/6A/7/8 are not involved. Additional data is also added to enhance the evidence for CNOT3 (Fig 2c).
(ii) Importantly, new data has been incorporated (Figure EV4b and Fig5F) which supports a role for CNOT1 in upregulation of host mRNA levels and increased stability. Fig 7d also shows a greater impact of CNOT1 on host compared to viral polyA tails

Therefore, the authors have added additional experimental evidence and amended text changes addressing the majority of reviewers concerns, as such the manuscript now merits publication in EMBO reports.

Minor points but not essential prior to publication - I would have liked to see a little more discussion about how CNOT expression is upregulated during HCMV infection. In addition, text changes on pg 16 (lines 462-464), could be expanded and strengthen to discuss potential role of mRNA targets.

'The authors have addressed the remaining points of the referees by expanding the discussion section and by clarifying the proposed mechanism (including a model schematic - new fig 7) and also addressed all minor editorial requests.

2nd Revision - Editorial Decision **28th Sep 2023**

Dr. Hannah Burgess
University of Surrey
United Kingdom

Dear Dr. Burgess,

I am very pleased to accept your manuscript for publication in the next available issue of EMBO reports. Thank you for your contribution to our journal.

At the end of this email I include important information about how to proceed. Please ensure that you take the time to read the information and complete and return the necessary forms to allow us to publish your manuscript as quickly as possible.

As part of the EMBO publication's Transparent Editorial Process, EMBO reports publishes online a Review Process File to accompany accepted manuscripts. As you are aware, this File will be published in conjunction with your paper and will include the referee reports, your point-by-point response and all pertinent correspondence relating to the manuscript.

If you do NOT want this File to be published, please inform the editorial office within 2 days, if you have not done so already, otherwise the File will be published by default [contact: emboreports@embo.org]. If you do opt out, the Review Process File link will point to the following statement: "No Review Process File is available with this article, as the authors have chosen not to make the review process public in this case."

Thank you again for your contribution to EMBO reports and congratulations on a successful publication. Please consider us again in the future for your most exciting work.

Yours sincerely,

Achim Breiling
Senior Editor
EMBO Reports

****

THINGS TO DO NOW:

Please note that you will be contacted by Wiley Author Services to complete licensing and payment information. The required 'Page Charges Authorization Form' is available here: https://www.embopress.org/pb-assets/embo-site/er_apc.pdf - please download and complete the form and return to embopressproduction@wiley.com

EMBO Press participates in many Publish and Read agreements that allow authors to publish Open Access with reduced/no publication charges. Check your eligibility: https://authorservices.wiley.com/author-resources/Journal-Authors/open-access/affiliation-policies-payments/index.html

You will receive proofs by e-mail approximately 2-3 weeks after all relevant files have been sent to our Production Office; you should return your corrections within 2 days of receiving the proofs.

Please inform us if there is likely to be any difficulty in reaching you at the above address at that time. Failure to meet our deadlines may result in a delay of publication, or publication without your corrections.

All further communications concerning your paper should quote reference number EMBOR-2022-56327V3 and be addressed to emboreports@wiley.com.

Should you be planning a Press Release on your article, please get in contact with emboreports@wiley.com as early as possible, in order to coordinate publication and release dates.

EMBO Press Author Checklist

Corresponding Author Name: Hannah Burgess
Journal Submitted to: EMBO reports
Manuscript Number: EMBOR-2022-56327V2

USEFUL LINKS FOR COMPLETING THIS FORM
The EMBO Journal - Author Guidelines
EMBO Reports - Author Guidelines
Molecular Systems Biology - Author Guidelines
EMBO Molecular Medicine - Author Guidelines

Reporting Checklist for Life Science Articles (updated January
This checklist is adapted from Materials Design Analysis Reporting (MDAR) Checklist for Authors. MDAR establishes a minimum set of requirements in transparent reporting in the life sciences (see Statement of Task: 10.31222/osf.io/9sm4x). Please follow the journal's guidelines in preparing your
Please note that a copy of this checklist will be published alongside your article.

Abridged guidelines for figures
1. Data
The data shown in figures should satisfy the following conditions:
- the data were obtained and processed according to the field's best practice and are presented to reflect the results of the experiments in an accurate and unbiased manner.
- ideally, figure panels should include only measurements that are directly comparable to each other and obtained with the same assay.
- plots include clearly labeled error bars for independent experiments and sample sizes. Unless justified, error bars should not be shown for technical
- if n<5, the individual data points from each experiment should be plotted. Any statistical test employed should be justified.
- Source Data should be included to report the data underlying figures according to the guidelines set out in the authorship guidelines on Data

2. Captions
Each figure caption should contain the following information, for each panel where they are relevant:
- a specification of the experimental system investigated (eg cell line, species name).
- the assay(s) and method(s) used to carry out the reported observations and measurements.
- an explicit mention of the biological and chemical entity(ies) that are being measured.
- an explicit mention of the biological and chemical entity(ies) that are altered/varied/perturbed in a controlled manner.
- the exact sample size (n) for each experimental group/condition, given as a number, not a range;
- a description of the sample collection allowing the reader to understand whether the samples represent technical or biological replicates (including how many animals, litters, cultures, etc.).
- a statement of how many times the experiment shown was independently replicated in the laboratory.
- definitions of statistical methods and measures:
  - common tests, such as t-test (please specify whether paired vs. unpaired), simple χ2 tests, Wilcoxon and Mann-Whitney tests, can be unambiguously identified by name only, but more complex techniques should be described in the methods section;
  - are tests one-sided or two-sided?
  - are there adjustments for multiple comparisons?
  - exact statistical test results, e.g., P values = x but not P values < x;
  - definition of 'center values' as median or average;
  - definition of error bars as s.d. or s.e.m.

Please complete ALL of the questions below.
Select "Not Applicable" only when the requested information is not relevant for your study.

Materials

Newly Created Materials	Information included in the manuscript?	In which section is the information available? (Reagents and Tools Table, Materials and Methods, Figures, Data Availability Section)
New materials and reagents need to be available; do any restrictions apply?	Not Applicable	

Antibodies	Information included in the manuscript?	In which section is the information available? (Reagents and Tools Table, Materials and Methods, Figures, Data Availability Section)
For **antibodies** provide the following information:<br>- Commercial antibodies: RRID (if possible) or supplier name, catalogue number and or/clone number<br>- Non-commercial: RRID or citation	Yes	Table 3

DNA and RNA sequences	Information included in the manuscript?	In which section is the information available? (Reagents and Tools Table, Materials and Methods, Figures, Data Availability Section)
**Short novel DNA or RNA including primers, probes**: provide the sequences.	Yes	Table 3

Cell materials	Information included in the manuscript?	In which section is the information available? (Reagents and Tools Table, Materials and Methods, Figures, Data Availability Section)
**Cell lines:** Provide species information, strain. Provide accession number in repository **OR** supplier name, catalog number, clone number, and/**OR** RRID.	Yes	Materials and Methods
**Primary cultures:** Provide species, strain, sex of origin, genetic modification status.	Not Applicable	
Report if the cell lines were recently **authenticated** (e.g., by STR profiling) and tested for mycoplasma contamination.	Yes	Materials and Methods

Experimental animals	Information included in the manuscript?	In which section is the information available? (Reagents and Tools Table, Materials and Methods, Figures, Data Availability Section)
**Laboratory animals or Model organisms:** Provide species, strain, sex, age, genetic modification status. Provide accession number in repository **OR** supplier name, catalog number, clone number, **OR** RRID.	Not Applicable	
**Animal observed in or captured from the field:** Provide species, sex, and age where possible.	Not Applicable	
Please detail **housing and husbandry conditions**.	Not Applicable	

Plants and microbes	Information included in the manuscript?	In which section is the information available? (Reagents and Tools Table, Materials and Methods, Figures, Data Availability Section)
**Plants:** provide species and strain, ecotype and cultivar where relevant, unique accession number if available, and source (including location for collected wild specimens).	Not Applicable	
**Microbes:** provide species and strain, unique accession number if available, and source.	Yes	Materials and Methods

Human research participants	Information included in the manuscript?	In which section is the information available? (Reagents and Tools Table, Materials and Methods, Figures, Data Availability Section)
If collected and within the bounds of privacy constraints report on age, sex and gender or ethnicity for all study participants.	Not Applicable	

Core facilities	Information included in the manuscript?	In which section is the information available? (Reagents and Tools Table, Materials and Methods, Figures, Data Availability Section)
If your work benefited from core facilities, was their service mentioned in the acknowledgments section?	Not Applicable	

Design

Study protocol	Information included in the manuscript?	In which section is the information available? (Reagents and Tools Table, Materials and Methods, Figures, Data Availability Section)

If study protocol has been **pre-registered, provide DOI in the manuscript**. For clinical trials, provide the trial registration number **OR** cite DOI.	Not Applicable	
Report the **clinical trial registration number** (at ClinicalTrials.gov or equivalent), where applicable.	Not Applicable	

Laboratory protocol	Information included in the manuscript?	In which section is the information available? (Reagents and Tools Table, Materials and Methods, Figures, Data Availability Section)
Provide DOI OR other citation details if **external detailed step-by-step protocols** are available.	Not Applicable	

Experimental study design and statistics	Information included in the manuscript?	In which section is the information available? (Reagents and Tools Table, Materials and Methods, Figures, Data Availability Section)
Include a statement about **sample size** estimate even if no statistical methods were used.	Not Applicable	
Were any steps taken to minimize the effects of subjective bias when allocating animals/samples to treatment (e.g. **randomization procedure**)? If yes, have they been described?	Not Applicable	
Include a statement about **blinding** even if no blinding was done.	Not Applicable	
Describe **inclusion/exclusion criteria** if samples or animals were excluded from the analysis. Were the criteria pre-established?<br><br>If sample or data points were omitted from analysis, report if this was due to attrition or intentional exclusion and provide justification.	Not Applicable	
For every figure, are **statistical tests** justified as appropriate? Do the data meet the assumptions of the tests (e.g., normal distribution)? Describe any methods used to assess it. Is there an estimate of variation within each group of data? Is the variance similar between the groups that are being statistically compared?	Yes	Figure Legends

Sample definition and in-laboratory replication	Information included in the manuscript?	In which section is the information available? (Reagents and Tools Table, Materials and Methods, Figures, Data Availability Section)
In the figure legends: state number of times the experiment was **replicated** in laboratory.	Yes	Figure Legends
In the figure legends: define whether data describe **technical or biological replicates.**	Yes	Figure Legends

Ethics

Ethics	Information included in the manuscript?	In which section is the information available? (Reagents and Tools Table, Materials and Methods, Figures, Data Availability Section)
Studies involving **human participants**: State details of **authority granting ethics approval** (IRB or equivalent committee(s), provide reference number for approval.	Not Applicable	
Studies involving **human participants**: Include a statement confirming that **informed consent** was obtained from all subjects and that the experiments conformed to the principles set out in the WMA Declaration of Helsinki and the Department of Health and Human Services Belmont Report.	Not Applicable	
Studies involving **human participants:** For publication of **patient photos**, include a statement confirming that consent to publish was obtained.	Not Applicable	
Studies involving experimental **animals**: State details of **authority granting ethics approval** (IRB or equivalent committee(s), provide reference number for approval. Include a statement of compliance with ethical regulations.	Not Applicable	
Studies involving **specimen and field samples:** State if relevant **permits** obtained, provide details of authority approving study; if none were required, explain why.	Not Applicable	

Dual Use Research of Concern (DURC)	Information included in the manuscript?	In which section is the information available? (Reagents and Tools Table, Materials and Methods, Figures, Data Availability Section)
Could your study fall under dual use research restrictions? Please check biosecurity documents and list of **select agents and toxins** (CDC): https://www.selectagents.gov/sat/list.htm	Not Applicable	
If you used a select agent, is the security level of the lab appropriate and reported in the manuscript?	Not Applicable	
If a study is subject to dual use research of concern regulations, is the name of the **authority granting approval and reference number** for the regulatory approval provided in the manuscript?	Not Applicable	

Reporting

The MDAR framework recommends adoption of discipline-specific guidelines, established and endorsed through community initiatives. Journals have their own policy about requiring specific guidelines and recommendations to complement MDAR.

Adherence to community standards	Information included in the manuscript?	In which section is the information available? (Reagents and Tools Table, Materials and Methods, Figures, Data Availability Section)
State if relevant guidelines or checklists (e.g., **ICMJE, MIBBI, ARRIVE, PRISMA**) have been followed or provided.	Not Applicable	
For **tumor marker prognostic studies,** we recommend that you follow the **REMARK** reporting guidelines (see link list at top right). See author guidelines, under 'Reporting Guidelines'. Please confirm you have followed these guidelines.	Not Applicable	
For **phase II and III randomized controlled trials**, please refer to the **CONSORT** flow diagram (see link list at top right) and submit the CONSORT checklist (see link list at top right) with your submission. See author guidelines, under 'Reporting Guidelines'. Please confirm you have submitted this list.	Not Applicable	

Data Availability

Data availability	Information included in the manuscript?	In which section is the information available? (Reagents and Tools Table, Materials and Methods, Figures, Data Availability Section)
Have **primary datasets** been deposited according to the journal's guidelines (see 'Data Deposition' section) and the respective accession numbers provided in the Data Availability Section?	Yes	Materials and Methods
Were **human clinical and genomic datasets** deposited in a public access-controlled repository in accordance to ethical obligations to the patients and to the applicable consent agreement?	Not Applicable	
Are **computational models** that are central and integral to a study available without restrictions in a machine-readable form? Were the relevant accession numbers or links provided?	Not Applicable	
If publicly available data were reused, provide the respective **data citations in the reference list**.	Not Applicable	
